# Ring opening of photogenerated azetidinols as a strategy for the synthesis of aminodioxolanes

**DOI:** 10.3762/bjoc.20.148

**Published:** 2024-07-19

**Authors:** Henning Maag, Daniel J Lemcke, Johannes M Wahl

**Affiliations:** 1 Department Chemie, Johannes Gutenberg-Universität, Duesbergweg 10–14, 55128 Mainz, Germany

**Keywords:** azetidine, Norrish–Yang cyclization, ring-opening reaction, ring strain

## Abstract

α-Aminoacetophenones are identified as promising building blocks for the synthesis of highly substituted dioxolanes. The presented strategy is founded on the build and release of molecular strain and achieves a formal transposition of a methyl group. During light irradiation, 3-phenylazetidinols are forged as reaction intermediates, which readily undergo ring opening upon the addition of electron-deficient ketones or boronic acids. Key to the successful development of this two-step process is the identification of a benzhydryl-protecting group, which orchestrates the photochemical Norrish–Yang cyclization and facilitates the subsequent ring opening.

## Introduction

**Scheme 1:**
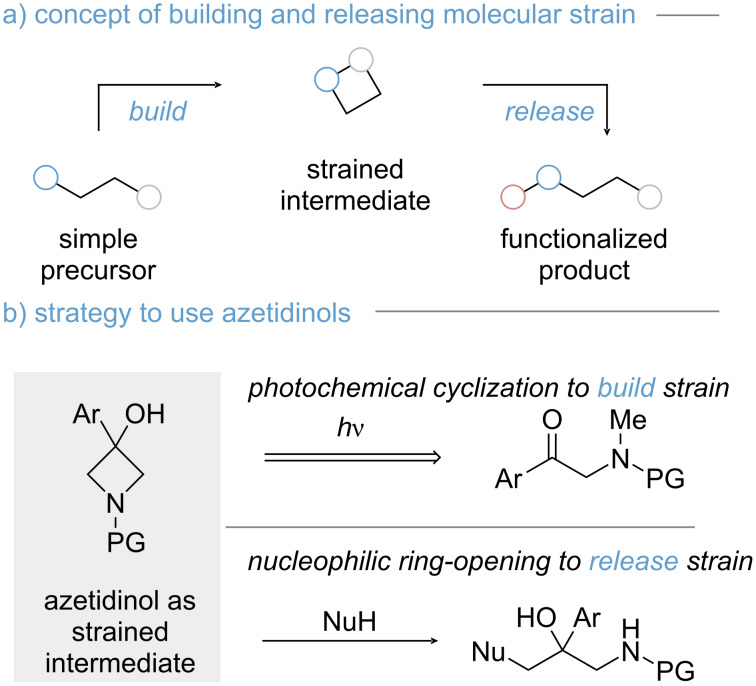
Build and release approach for the functionalization of simple precursors. a) General overview. b) Embedding azetidines into the general synthetic strategy.

Identifying efficient methods for the preparation of densely functionalized molecules is one of the central goals of modern organic chemistry. In this context, application of strained molecules has garnered increasing attention due to their intrinsic reactivity [1–2]. However, accessing strained molecules continues to pose a synthetic challenge because many reported methods require harsh conditions for the preparation. An attractive alternative to traditional synthesis can be found in photochemical methods, bypassing energetic constraints by the utilization of photon energy. Thus, endergonic transformations can be realized [3]. A promising strategy for the synthesis of complex products lies in the combination of photochemical cyclization and strain-release reaction (Scheme 1a) [4]. In this ‘build and release approach’, a simple precursor is cyclized upon irradiation. Subsequently, a functionalization step such as a ring-opening event is implemented, facilitated by the pre-installed strain energy of the four-membered ring [5–7].

The implementation of a build and release strategy, as depicted in Scheme 1a, necessitates the full compatibility of both individual reaction steps, thus placing stringent demands on the selection and assessment of substrates. In our quest to identify a suitable system, we were attracted by the use of azetidines as potential reaction intermediates (Scheme 1b) [8–10]. Azetidines were chosen because of existing literature precedent for their photochemical assembly by Norrish–Yang cyclization, which employs easily accessible α-aminoacetophenones as starting materials [11–15]. Furthermore, ring-opening reactions of azetidines have been recently achieved using sulfur and oxygen nucleophiles [16–25]. However, methods that target a combination of photochemical ring closure and subsequent functionalization are still underdeveloped [4–7]. Within this work, we describe our endeavours in identifying a suitable substrate to meet the photochemical requirements of the Norrish–Yang cyclization and allow for subsequent ring-opening reactions. In this regard, we uncovered a novel entry to dioxolanes by intramolecular ring opening of azetidines using ketones and boronic acids.

## Results and Discussion

### Photocyclization

**Scheme 2:**
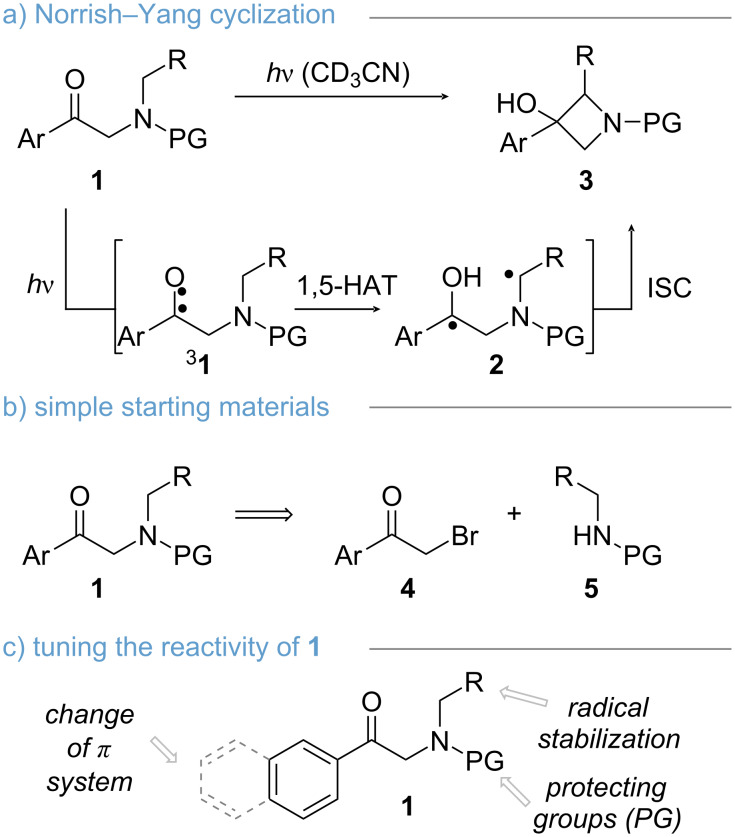
Modularity of the Norrish–Yang cyclization for the synthesis of azetidines.

We initiated our study by a systematic investigating of the Norrish–Yang cyclization for the synthesis of azetidinols. Mechanistically, the Norrish–Yang cyclization involves a 1,5-hydrogen abstraction (HAT) step followed by ring closure to forge the azetidine scaffold (Scheme 2a, **1** → **3**, via 1,4-biradical **2**) [26]. The respective α-aminoacetophenones **1** were synthesized using a modular approach starting from α-bromoacetophenones **4** and disubstituted amines **5** (Scheme 2b). Three different criteria were chosen to evaluate the photochemical cyclization (Scheme 2c). We hypothesized that changes in the π-system of the aromatic ring would have an impact on the efficiency of triplet generation (**1** → **^3^1**). The corresponding protecting group (PG) was thought to control the conformation of the 1,4-biradical **2**, which is known to be important for efficient Norrish–Yang cyclizations [27–28]. Furthermore, the PG was deemed crucial for the development of further functionalizations of the azetidinols (vide infra). Thirdly, we wanted to assess substitution α to the nitrogen (R), as this was thought to impact the stability of the intermediary 1,4-biradical **2** [29–30]. To obtain comparable results, all reactions were performed in a LuzChem photoreactor under identical conditions. Deuterated acetonitrile was chosen as a preferred solvent because it is known to facilitate Norrish–Yang-cyclizations and allows simple analysis by nuclear magnetic resonance (NMR) spectroscopy [10].

Our results are summarized in Table 1. When *p*-toluenesulfonyl (Ts) was used as a PG at nitrogen, azetidine **3a** was obtained in 81% yield along with 14% acetophenone (**6**) as the major side product (Table 1, entry 1). Acetophenone (**6**) is thought to be generated by a Norrish type II cleavage of biradical **2a**. Changes at the aromatic core, as demonstrated by acetonaphthone **1b**, resulted in low conversions (Table 1, entry 2). When extending the reaction time to reach full conversion, significant amounts of Norrish type II cleavage were obtained and product **3b** was isolated in only 16% yield. This substrate was therefore abandoned. Interestingly, ethyl (**1c**) and benzyl (**1d**) substitution was detrimental to the cyclization, indicated by the 54% and 63% yield, respectively (Table 1, entries 3 and 4). We observed an increase in Norrish II fragmentation in these cases. The observed diastereoselectivity was poor, supporting the radical character of the ring-closing event. A similar trend was observed when studying the methanesulfonyl (Ms) protecting group (entries 5–7 in Table 1). When changing to phenyl substitution at the nitrogen, azetidinol **3h** was formed in 12% yield with a substantial amount of unselective side reactions (Table 1, entry 8). By introducing an ethyl group (**1i**), only 27% conversion were achieved, with 2% product formation (Table 1, entry 9). The notable influence of steric and electronic parameters of the PG prompted us to investigate a range of sterically diverse alkyl substituents. Interestingly, while only fragmentation products were obtained for methyl and benzyl groups (Table 1, entries 10 and 11), the sterically demanding benzhydryl (Bzh) group showed progress towards azetidinol **3l** with a yield of 10% (Table 1, entry 12).

**Table 1:** Comparison of the effect of structural variations on the photoreaction.

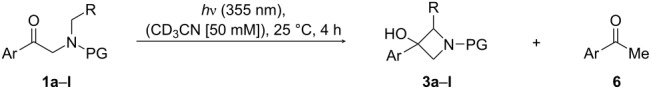						

Entry	Substrates **1**			Conversion (%)	Yield **3** (%)	Yield **6** (%)

1	Ar = Ph	PG = Ts	R = H (**1a**)	>99	81	14
2	Ar = Nph	PG = Ts	R = H (**1b**)	23	11	4

3	Ar = Ph	PG = Ts	R = CH_3_ (**1c**)	>99	54 (dr 54:46)	26
4	Ar = Ph	PG = Ts	R = Ph (**1d**)	>99	63 (dr 60:40)	20

5	Ar = Ph	PG = Ms	R = H (**1e**)	>99	67	14
6	Ar = Ph	PG = Ms	R = CH_3_ (**1f**)	>99	44 (dr 51:49)	42
7	Ar = Ph	PG = Ms	R = Ph (**1g**)	>99	37 (dr 52:48)	22

8	Ar = Ph	PG = Ph	R = H (**1h**)	90	12	3
9	Ar = Ph	PG = Ph	R = CH_3_ (**1i**)	27	2 (dr 52:48)	6

10	Ar = Ph	PG = Me	R = H (**1j**)	87	<1	53
11	Ar = Ph	PG = Bn	R = H (**1k**)	>99	<1	65
12	Ar = Ph	PG = Bzh	R = H (**1l**)	94	10	74

^a^Conversions and yields were determined by ^1^H NMR spectroscopy using mesitylene as an internal standard. ^b^Diastereomeric ratios were determined by ^1^H NMR spectroscopy. Reactions were run on 25 µM scale. Ts = *p*-toluenesulfonyl. Nph = 2-naphthyl. Ms = methanesulfonyl. Bzh = benzhydryl = CH(Ph)_2_.

As it can be extracted from our study, sulfonyl-based protecting groups hold a unique role in the Norrish–Yang cyclization of α-aminoacetophenones, which was underpinned by the good performances of substrates **1a** and **1e**. On the other hand, increasing steric demand at the α-aminocarbon has a detrimental effect on the cyclization, as indicated by the poor performances of ethyl and benzyl substitution (**1c** and **1d**). In contrast, sterically demanding groups at the nitrogen can have a positive effect on product formation, potentially by changing the substrate’s conformation.

### Ring opening

**Scheme 3:**
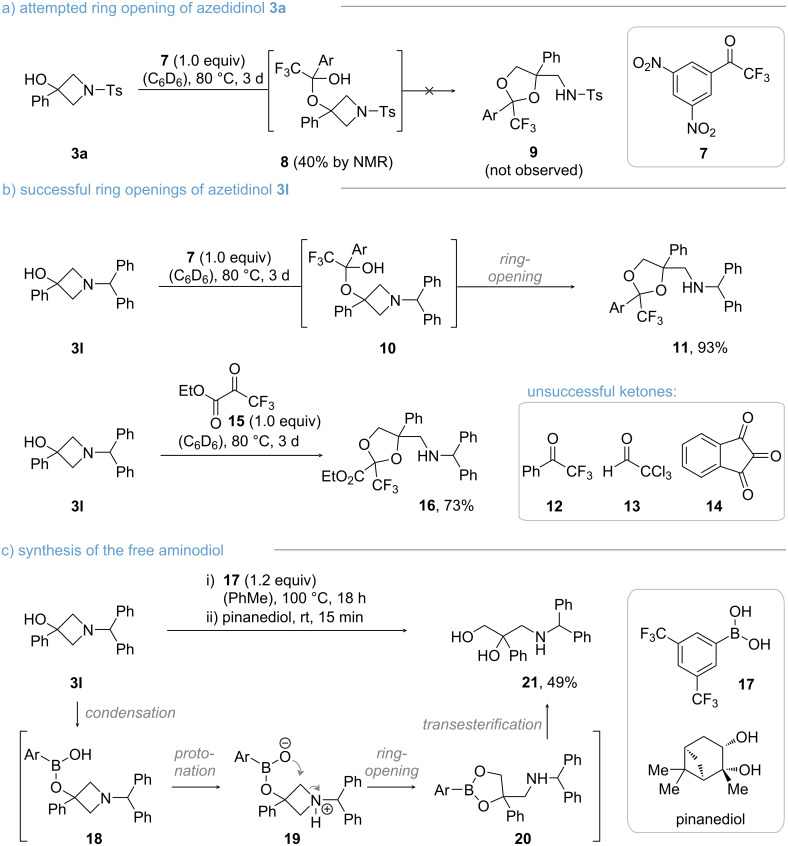
Ring-opening reactions using electron-deficient ketones and boronic acids.

Based on its outstanding performance in the photoreaction, we selected Ts-protected azetidinol **3a** as our preferred substrate to test ring-opening reactions. However, initial attempts were met with limited success. Being conscious about the challenges associated with intermolecular ring openings at four-membered heterocycles, we considered an in situ tethering approach, which has proved an attractive alternative in our recent study on oxetane-ring openings [31]. Therefore, electron-deficient ketone **7** was added to **3a** (Scheme 3a). Formation of hemiketal **8** was expected to occur, which would facilitate ring opening by a 5-*exo*-*tet* cyclization. While we did observe the formation of hemiketal **8** by NMR spectroscopy, we were unable to detect any ring-opened products **9**, even when adding Lewis acids or Brønsted acids to the reaction mixture (see Supporting Information File 1 for further details). Finally, we decided to evaluate the electronic effects of the PG on the ring opening, which is why we opted to try the benzhydryl-protected substrate **3l** in our envisioned transformation. Several recent publications have indicated the unique role of the benzhydryl group for azetidinol reactivity [32–34]. To our delight, **3l** cleanly underwent ring opening via hemiketal **10** to deliver dioxolane **11** as a single diastereomer in 93% yield (Scheme 3b). With this result at hand, we tested several other carbonyls known to form stable hemiacetals [35–40]. While we were unable to isolate ring-opened products from trifluoroacetophenone (**12**), chloral (**13**), and indane-1,2,3-trione (**14**), ethyl trifluoropyruvate (**15**) furnished the desired dioxolane **16**, which was isolated as a mixture of diastereomers in 73% yield. Mechanistically, we believe that the hemiketal protonates the azetidine before a nucleophilic attack can occur. Therefore, the ring opening is critical to the acidity of the transient hemiacetals and to the basicity of the respective azetidines, explaining the unsuccessful attempts with **3a**.

Dioxolanes **11** and **16** are inherently stable and we were unable to cleave the ketal after reaction. Thus, we investigated the feasibility to use boronic acids in the ring-opening reaction (Scheme 3c). Phenylboronic acid **17** is known to exhibit a similar acidity as the corresponding hemiketals providing a promising starting point for pursuing this ring-opening attempt [41]. We hypothesized that this approach would allow the synthesis of 3-amino-1,2-diols, a motif commonly found in pharmaceuticals such as, e.g., β-blockers [42]. Our envisioned one-pot protocol features protonation of the azetidine from boronic monoester (**18** → **19**) followed by ring opening, in accordance to the hemiketal reactions. We decided to directly cleave dioxaborolane **20** after the reaction has stalled to facilitate separation. After a brief optimization of the reaction conditions (details are provided in Supporting Information File 1), we successfully conducted the desired sequence by raising the temperature to 100 °C to initiate ring opening, and employing a mild transesterification method for diol release [43–45]. Thus, we were able to isolate 3-amino-1,2-diol **21** in 49% yield.

## Conclusion

Within this work, we demonstrated that the build and release of strain energy can be combined in a simple reaction sequence when appropriately tuning the reaction parameters. Our system relies on a photochemical Norrish–Yang cyclization of α-aminoacetophenones, which provides a sustainable entry to highly strained azetidinols. It was shown that subsequent ring opening can be triggered by the addition of electron-deficient ketones or boronic acids, which resembles a novel strategy for azetidinol desymmetrization. The nature of the protecting group was found to be critical for the two-step process to be effective, which resulted in the synthesis of highly functionalized dioxolanes and 3-amino-1,2-diols. We believe that the concept of building and releasing strain energy, particularly when paired with photochemical means, is of general interest and its structural simplicity should allow for an expansion to other substrates.

## Supporting Information

File 1 Experimental procedures, characterization data and copies of NMR spectra.

## Data Availability

All data that supports the findings of this study is available in the published article and/or the supporting information to this article.
