# Supplementary material for: Ring opening of photogenerated azetidinols as a strategy for the synthesis of aminodioxolanes
Source: Beilstein J Org Chem. 2024 Jul 19;20:1671–6. doi: 10.3762/bjoc.20.148 (PMC11285075; doi:10.3762/bjoc.20.148)

BEILSTEIN JOURNAL OF ORGANIC CHEMISTRY

Supporting Information

for

Ring opening of photogenerated azetidinols as a strategy for the synthesis of aminodioxolanes

Henning Maag, Daniel J. Lemcke and Johannes M. Wahl

Beilstein J. Org. Chem. **2024,** *20,* 1671–1676. doi:10.3762/bjoc.20.148

Experimental procedures, characterization data and copies of NMR spectra

License and Terms: This is a supporting information file under the terms of the Creative Commons Attribution License (https://creativecommons.org/licenses/by/4.0). Please note that the reuse, redistribution and reproduction in particular requires that the author(s) and source are credited and that individual graphics may be subject to special legal provisions.
The license is subject to the *Beilstein Journal of Organic Chemistry* terms and conditions: (https://www.beilstein-journals.org/bjoc/terms)

Table of contents

1. General S1
2. Synthesis of starting materials S2
3. Substrate scope photocyclization S14
4. Ring opening S22
5. References S28
6. UV Spectra S29
7. NMR Spectra S35

1. General

Reaction setup: Chemicals were purchased from Alfa Aesar, Acros Organics, Sigma Aldrich, BLDpharm, FluoroChem, Carbolution or ABCR and were (unless otherwise stated) used as received. All reactions involving air or moisture-sensitive reagents were carried out in oven (125 °C) and flame-dried glassware under nitrogen atmosphere using standard Schlenk techniques. Dry solvents were collected from an MBraun MB SPS-800 (Et_2O: MB-KOL-A and MB-KOL MT2-250, THF: 2 × MB-KOL MT2-150 °C, CH_2Cl_2: 2 × MB-KOL-A). A positive argon pressure was used to pass the solvents through the columns. Unless otherwise noted, all work-up and purification procedures were carried out with pre-distilled technical grade solvents. Purification was performed either with standard column chromatography techniques using Geduran® Si 60 silica gel (0.063–0.200 mm, Merck), on an automated flash chromatography (FC) system Biotage Isolera One utilizing Biotage Sfär Silica D-Duo 60 µm columns (5 g, 25 g, 100 g) or on an automated flash chromatography system Teledyne Isco with Biotage Sfär Silica C18-Duo 100 Å 30 µm columns (12 g) (RP-MPLC). Glass silica gel plates 60 F254 (Merck) were used for analytic thin layer chromatography applying either UV light (254/366 nm), $KMnO_4$ (1.5 g $KMnO_4$, 5 g $NaHCO_3$ and 5 mL NaOH 10% in 200 mL H_2O), CAM (0.5g $Ce(NH_4)_2(NO_3)_6$ and 24.0 g of $(NH_4)_6Mo_7O_{24}\cdot 4H_2O$, 28 mL H_2SO_4 in 200 mL H_2O) for detection. Photochemical reactions were performed in a Luzchem LZC-ORG photoreactor with 10 × 8 Watt Luzchem LZC-355 mercury lamps.

Analytical methods: Melting points (M.P.) were measured on a *Büchi* B-540 melting-point apparatus and are reported uncorrected. Infrared (IR) spectra were obtained on a Tensor 27 spectrometer (*Bruker*) using a diamond ATR unit and are reported in wavenumbers (cm^{-1}). Bands are characterized as broad (br), strong (s), medium (m), and weak (w). Nuclear magnetic resonance (NMR) spectra were recorded by the analytical department of the Department Chemie at Johannes Gutenberg-Universität Mainz. The following spectrometers were used: Avance III HD 300 (Bruker), Avance II 400 (Bruker), Avance III HD 400 (Bruker), and Avance III 600 equipped with a cryo-probe head (Bruker). Spectra were recorded at 22 °C (unless otherwise noted). Chemical shifts are reported in ppm with the solvent resonance as the internal standard (^1H NMR $CHCl_3$: δ = 7.26 ppm, C_6HD_5: δ = 7.16 ppm, $(CHD_2)(CD_3)SO$: δ = 2.50 ppm; CHD_2CN: δ = 1.94 ppm, ^{13}C NMR $CDCl_3$: δ = 77.16 ppm, C_6D_6: δ = 128.06 ppm, $(CD_3)_2SO$: δ = 39.5 ppm, CD_3CN: δ = 118.26 ppm). Chemical shifts of ^{19}F NMR are referenced to internal or external standards according to Togni and coworkers [1]. The data is reported as follows: chemical shift, multiplicity (s = singlet, d = doublet, t = triplet, q = quartet, p = pentet, br = broad, m = multiplet or combinations of these), coupling constants (Hz) and integration. Apparent multiplicity, which occurs as a result of accidental equality of coupling constants to magnetically non-equivalent protons, is marked as *app.* High resolution mass spectrometry (HRMS) was performed by the analytical department of the Department Chemie at Johannes Gutenberg-Universität Mainz. Spectra were recorded on a Thermo-Fisher Scientific DFS (GC–MS, ionization via electron ionization (EI) or chemical ionization (CI)) or on an Agilent 6545 Q-ToF (LC–MS, ionization via electron spray ionization (ESI), atmospheric-pressure chemical ionization (APCI)). Signals are reported as mass to charge ratio *m/z.* Absorption spectra (UV) were recorded using a Perkin Elmer LAMBDA 365 instrument.

2. Synthesis of starting materials

General procedure **A** for the preparation of α-aminoacetophenones from α-bromoacetophenones:

α-Aminoacetophenones were prepared following a literature known protocol with minor alterations [2]. A secondary amine (1.00 equiv) was dissolved in acetonitrile (1 M) and an α-bromoacetophenone (1.00 equiv) and K_2CO_3 (2.00 equiv) were added. The mixture was stirred at rt for 16 h, the solids were filtered-off and washed with acetonitrile. The solvent was removed under reduced pressure and the desired product was isolated after flash chromatography (FC), automated FC, reversed-phase medium pressure liquid chromatography (RP-MPLC) or recrystallization.

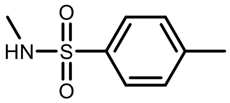

***N*-Methyl-*p*-toluenesulfonamide (S1)**

N-Methyl-*p*-toluenesulfonamide was prepared following a literature known protocol [2]. *p*-Toluenesulfonyl chloride (6.08 g, 31.87 mmol, 1.00 equiv) was dissolved in Et_2O (30 mL) and an aqueous solution of methylamine (40 wt %, 6.34 mL, 73.87 mmol, 2.32 equiv) was added at 0 °C. The mixture was stirred at rt for 16 h and water (30 mL) was added. The phases were separated, and the aqueous layer was extracted with EtOAc (3 × 40 mL). The combined organic fractions were washed with brine (40 mL) and were dried over $MgSO_4$. The solvent was removed under reduced pressure and the crude product was purified via automated FC (CyH/EtOAc 100:0 to 60:40) and was obtained as a colourless solid (5.68 g, 30.7 mmol, 96%).

^{1}H NMR (400 MHz, $CDCl_3$): δ = 7.77 – 7.72 (m, 2H)), 7.35 – 7.29 (m, 2H), 4.26 (s, 1H), 2.65 (d, J = 4.5 Hz, 3H), 2.43 (s, 3H). **^{13}C NMR (101 MHz, $CDCl_3$)**: δ = 143.7, 135.9, 129.9, 127.4, 29.5, 21.7. Spectroscopic data was in agreement to those previously reported. [3]

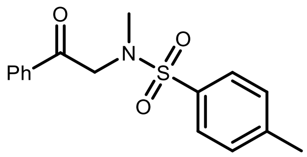

***N*,4-Dimethyl-*N*-(2-oxo-2-phenylethyl)benzenesulfonamide (1a)**

Following general procedure **A** with minor alterations, using *N*-methyl-*p*-toluenesulfonamide (**S1**, 1.85 g, 10.0 mmol, 1.00 equiv), 2-bromoacetophenone (2.00 g, 10 mmol, 1.00 equiv) and K_2CO_3 (1.30 g, 10 mmol, 1.00 equiv) in acetonitrile

(20 mL). The desired product was obtained after automated FC (CyH/EtOAc 100:0 to 90:10) as a colourless solid (1.90 g, 6.24 mmol, 62%).

^{1}H NMR (400 MHz, $CDCl_3$): δ = 8.01 – 7.94 (m, 2H), 7.76 – 7.69 (m, 2H), 7.65 – 7.57 (m, 1H), 7.52 – 7.44 (m, 2H), 7.36 – 7.31 (m, 2H), 4.57 (s, 2H), 2.83 (s, 3H), 2.45 (s, 3H). **^{13}C NMR (101 MHz, $CDCl_3$)**: δ = 193.9, 143.8, 134.9, 134.0, 129.8, 129.0, 128.4, 127.7, 56.2, 35.7, 21.7.[a] Spectroscopic data was in agreement to those previously reported.[2]

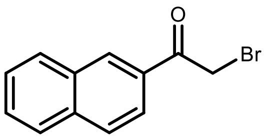

2-Bromo-1-(naphthalen-2-yl)ethan-1-one (S2)

2-Bromo-1-(naphthalen-2-yl)ethan-1-one was prepared following a literature known protocol.[4] 1-Naphthalen-2-ylethanone (1.70 g, 10.0 mmol, 1.00 equiv) and silica gel (170 mg, 2.83 mmol, 0.283 equiv, 10 wt %) were dissolved in methanol (20.0 mL) in a 250 mL three-necked flask under inert atmosphere. The mixture was heated to reflux and *N*-bromosuccinimide (2.14 g, 12.0 mmol, 1.20 equiv) was added in 5 portions over 30 min. After additional 30 min the mixture was allowed to cool to rt and was filtered. The solvent was removed under reduced pressure and the crude product was purified via FC (pentane/toluene 50:50 to 25:75). The product was obtained as an off-white solid (1.863 g, 7.48 mmol, 75%).

M.P.: 80 – 83 °C. **IR (neat)**: $\tilde{\nu}$ = 3059 (w), 2944 (w), 1676 (s), 1626 (m), 1595 (w), 1577 (w), 1507 (w), 1469 (m), 1435 (w), 1390 (w), 1355 (w), 1291 (m), 1234 (w), 1181 (m), 1157 (w), 1124 (w), 1105 (w), 1027 (w), 992 (w), 943 (w), 917 (w), 855 (w), 821 (m), 775 (w), 761 (w), 682 (w), 625 (w), 588 (w), 564 (w), 531 (w), 476 (m), 422 (w). **^{1}H NMR (600 MHz, $CDCl_3$)**: δ = 8.53 – 8.51 (m, 1H), 8.03 (dd, J = 8.6, 1.8 Hz, 1H), 7.99 (dd, J = 8.2, 1.3 Hz, 1H), 7.93 (d, J = 8.6 Hz, 1H), 7.90 (dd, J = 8.2, 1.2 Hz, 1H), 7.64 (ddd, J = 8.2, 6.8, 1.3 Hz, 1H), 7.58 (ddd, J = 8.1, 6.8, 1.3 Hz, 1H), 4.59 (s, 2H). **^{13}C NMR (151 MHz, $CDCl_3$)**: δ = 191.5, 136.0, 132.5, 131.4, 131.1, 129.8, 129.2, 129.0, 128.0, 127.2, 124.3, 31.1. **HRMS (ESI)**: Calculated for $C_{12}H_{10}BrO$ [M+H]$^+$: 248.9910, found: 248.9904. Spectroscopic data was in agreement to those previously reported.[5]

---

[a] Signals missing due to signal overlapping.

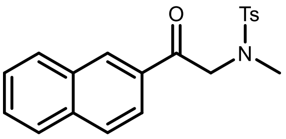

_N_,4-Dimethyl-_N_-(2-(naphthalen-2-yl)-2-oxoethyl)benzenesulfonamide (1b)

Following general procedure **A**, using 2-bromo-2'-acetonaphthone (**S2**, 498 mg, 2.00 mmol, 1.00 equiv), *N*-methyl-*p*-toluenesulfonamide (**S1**, 370 mg, 2.00 mmol, 1.00 equiv) and K_2CO_3 (415 mg, 3.00 mmol, 1.50 equiv). The desired product was obtained after automated FC (CyH/EtOAc 100:00 to 80:20) as an off-white solid (537 mg, 1.52 mmol, 76%).

M.P.: 135 – 141 °C. **IR (neat)**: $\tilde{\nu}$ = 3060 (w), 2915 (w), 1696 (m), 1627 (w), 1597 (w), 1470 (w), 1336 (m), 1306 (w), 1277 (w), 1258 (w), 1216 (w), 1187 (m), 1162 (s), 1123 (w), 1089 (m), 1019 (w), 997 (w), 980 (w), 932 (m), 862 (w), 817 (m), 749 (w), 728 (m), 654 (m), 549 (m), 476 (w). **^{1}H NMR (400 MHz, $CDCl_3$)**: δ = 8.56 (d, *J* = 1.8 Hz, 1H), 8.05 – 7.97 (m, 2H), 7.95 – 7.86 (m, 2H), 7.81 – 7.73 (m, 2H), 7.60 (dddd, *J* = 22.0, 8.2, 6.9, 1.3 Hz, 2H), 7.40 – 7.31 (m, 2H), 4.70 (s, 2H), 2.87 (s, 3H), 2.45 (s, 3H). **^{13}C NMR (101 MHz, $CDCl_3$)**: δ = 193.9, 143.8, 136.0, 134.9, 132.5, 132.2, 130.5, 129.9, 129.9, 129.1, 128.9, 128.0, 127.8, 127.2, 123.8, 56.3, 35.8, 21.7. **HRMS (ESI)**: Calculated for $C_{20}H_{20}NO_3S$ $[M+H]^+$: 354.1158, found: 354.1151.

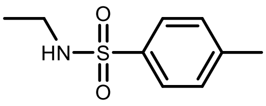

_N_-Ethyl-4-methylbenzenesulfonamide (S3)

p-Toluenesulfonyl chloride (381 mg, 2.00 mmol, 1.00 equiv) and ethylamine (69%, 303 mg, 374 µL, 4.64 mmol, 2.32 equiv) were dissolved in Et_2O (5.00 mL) and the mixture was stirred for 18 h. Water was added (5 mL) and the aqueous phase was extracted with EtOAc (3 × 15 mL). The combined organic fractions were dried over $MgSO_4$, and the solvent was removed under reduced pressure. The crude product was used without further purification.

^{1}H NMR (400 MHz, $CDCl_3$): δ = 7.79 – 7.71 (m, 2H), 7.35 – 7.28 (m, 2H), 4.29 (s, 1H), 3.00 (qd, *J* = 7.2, 6.0 Hz, 2H), 2.43 (s, 3H), 1.10 (t, *J* = 7.2 Hz, 3H). **^{13}C NMR (101 MHz, $CDCl_3$)**: δ =143.5, 137.1, 129.8, 127.3, 38.4, 21.7, 15.2. Spectroscopic data was in agreement to those previously reported.[6]

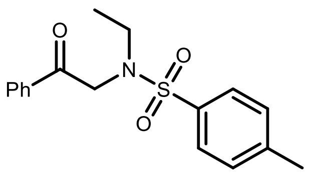

***N*-Ethyl-4-methyl-*N*-(2-oxo-2-phenylethyl)benzenesulfonamide (1c)**

Following general procedure **A**, using the crude product **S3** without further purification, 2-bromoacetophenone (398 mg, 2.00 mmol, 1.00 equiv) and K_2CO_3 (553 mg, 4.00 mmol, 2.00 equiv) in acetonitrile (2 mL). The desired product was obtained after automated FC (CyH/EtOAc 100:0 to 90:10) as a colourless solid (437 mg, 1.38 mmol, 69%).

M.P.: 95 – 98 °C. **IR (neat)**: $\tilde{\nu}$ = 3064 (w), 2977 (w), 2930 (w), 2876 (w), 1701 (m), 1597 (w), 1580 (w), 1494 (w), 1449 (w), 1415 (w), 1383 (w), 1332 (m), 1306 (w), 1289 (w), 1225 (m), 1209 (m), 1183 (w), 1155 (s), 1121 (w), 1090 (m), 1041 (w), 1019 (w), 1001 (w), 970 (m), 932 (w), 894 (m), 815 (m), 787 (w), 754 (m), 735 (s), 690 (m), 669 (m), 642 (m), 569 (w), 548 (s), 483 (w). **^{1}H NMR (400 MHz, $CDCl_3$)**: δ = 7.99 – 7.92 (m, 2H), 7.79 – 7.72 (m, 2H), 7.64 – 7.56 (m, 1H), 7.53 – 7.43 (m, 2H), 7.34 – 7.28 (m, 2H), 4.75 (s, 2H), 3.33 (q, J = 7.2 Hz, 2H), 2.43 (s, 3H), 1.09 (t, J = 7.2 Hz, 3H). **^{13}C NMR (101 MHz, $CDCl_3$)**: δ = 194.3, 143.5, 137.0, 135.0, 133.9, 129.7, 129.0, 128.2, 127.6, 52.5, 43.2, 21.7, 13.5. **HRMS (ESI)**: Calculated for $C_{17}H_{20}NO_3S$ $[M+H]^+$: 318.1158 found 318.1155.

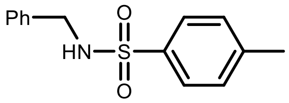

***N*-Benzyl-4-methylbenzenesulfonamide (S4)**

N-Benzyl-4-methylbenzenesulfonamide was prepared following a literature known protocol [7]. Benzylamine (107 mg, 109 µL, 1.00 mmol, 1.00 equiv) and triethylamine (152 mg, 209 µL, 1.50 mmol, 1.50 equiv) were dissolved in dichloromethane (1 mL) and *p*-toluenesulfonyl chloride (191 mg, 1.00 mmol, 1.00 equiv) was added dropwise as a solution in dichloromethane (3 mL). The reaction mixture was stirred for 3 h and water (5 mL) was added. The aqueous phase was extracted with dichloromethane (3 × 10 mL) and the combined organic fractions were dried over $MgSO_4$. The solvent was removed under reduced pressure and the mixture was purified via automated FC (CyH/EtOAc 100:0 to 40:60) and was obtained as a colourless solid (212 mg, 811 µmol, 81%).

M.P.: 115 – 118 °C. **IR (neat)**: $\tilde{\nu}$ = 3269 (s), 3051 (w), 3034 (w), 2916 (w), 1598 (w), 1496 (w), 1455 (m), 1422 (m), 1381 (w), 1364 (w), 1322 (s), 1289 (w), 1177 (m), 1164 (s), 1094 (w), 1083 (m), 1060 (m), 1029 (w), 875 (m), 812 (w), 741 (s), 703 (m), 683 (m), 601 (m), 551 (s), 541 (m), 519 (w), 478 (w), 461 (w). **^{1}H NMR (400 MHz, $CDCl_3$)**:

δ = 7.87 – 7.71 (m, 2H), 7.34 – 7.29 (m, 2H), 7.29 – 7.24 (m, 3H), 7.23 – 7.17 (m, 2H), 4.63 (t, *J* = 6.2 Hz, 1H), 4.12 (d, *J* = 6.1 Hz, 2H), 2.44 (s, 3H). **^{13}C NMR (101 MHz, $CDCl_3$)**: δ = 143.7, 137.0, 136.4, 129.9, 128.9, 128.1, 128.0, 127.3, 47.4, 21.7. **HRMS (ESI)**: Calculated for $C_{14}H_{16}NO_2S$ $[M+H]^+$: 262.0896, found: 262.0894. Spectroscopic data was in agreement to those previously reported [8].

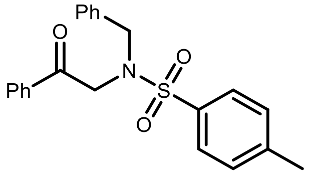

N-Benzyl-4-methyl-N-(2-oxo-2-phenylethyl)benzenesulfonamide (1d)

Following general procedure **A**, using *N*-benzyl-*p*-toluenesulfonamide (**S4**, 1.31 g, 5.00 mmol, 1.00 equiv), 2-bromoacetophenone (995 mg, 5.00 mmol, 1.00 equiv) and K_2CO_3 (1.38 g, 10.0 mmol, 2.00 equiv) in acetonitrile (10.0 mL). The desired product was obtained after FC (pentane/EtOAc 100:20) and recrystallisation from CyH/toluene 90:10 as a colourless solid (650 mg, 1.71 mmol, 34%).

M.P.: 116 – 118 °C. **IR (neat)**: $\tilde{\nu}$ = 3064 (w), 3031 (w), 2923 (w), 1699 (m), 1598 (w), 1581 (w), 1495 (w), 1449 (w), 1404 (w), 1337 (m), 1306 (w), 1289 (w), 1226 (m), 1156 (s), 1094 (m), 1058 (w), 1029 (w), 993 (w), 936 (w), 904 (w), 814 (w), 752 (m), 739 (m), 691 (m), 670 (m), 651 (w), 603 (w), 550 (m). **^{1}H NMR (400 MHz, DMSO-d_6)**: δ = 7.87 – 7.82 (m, 2H), 7.78 – 7.73 (m, 2H), 7.67 – 7.58 (m, 1H), 7.52 – 7.44 (m, 2H), 7.44 – 7.36 (m, 2H), 7.29 – 7.22 (m, 3H), 7.21 – 7.15 (m, 2H), 4.71 (s, 2H), 4.41 (s, 2H), 2.42 (s, 3H). **^{13}C NMR (101 MHz, DMSO-d_6)**: δ = 193.9, 143.1, 137.0, 135.8, 134.6, 133.6, 129.6, 128.7, 128.4, 128.3, 127.9, 127.6, 127.2, 53.1, 51.6, 21.0. **HRMS (ESI)**: Calculated for $C_{22}H_{22}NO_3S$ $[M+H]^+$ 380.1315, found: 380.1309. Spectroscopic data (^{1}H NMR) was in agreement to those previously reported [9].

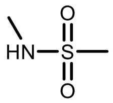

N-Methylmethanesulfonamide (S5)

Methanesulfonyl chloride (1.15 g, 774 µL, 10.0 mmol, 1.00 equiv) was dissolved in dry THF under nitrogen atmosphere and methylamine in THF (932 mg, 15.0 mL, 30.0 mmol, 2.00 M, 3.00 equiv) was added dropwise over 15 min at 0 °C. The mixture was stirred at 0 °C for 15 min and then at rt overnight. The solvent was removed under reduced pressure and the crude product was purified via bulb-to-bulb distillation (0.87 mbar, 125 °C to 140 °C) to obtain a colourless oil (1.04 g, 9.53 mmol, 95%).

IR (neat): $\tilde{\nu}$ = 3578 (w), 3295 (w), 3020 (w), 2937 (w), 2823 (w), 1633 (w), 1464 (w), 1406 (w), 1305 (s), 1148 (s), 1130 (s), 1070 (m), 971 (m), 836 (m), 754 (m), 643 (w), 521 (s), 456 (m). **^{1}H NMR (600 MHz, $CDCl_3$)**: δ = 4.23 (s, 1H), 2.95 (s, 3H), 2.84 (s, 3H). **^{13}C NMR (151 MHz, $CDCl_3$)**: δ = 39.0, 29.5. **HRMS (ESI)**: Calculated for $C_2H_6NO_2S$ [M-H]$^-$ 108.0125, found: 108.0116. Spectroscopic data was in agreement to those previously reported [10].

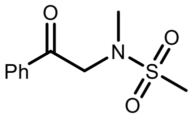

***N*-Methyl-*N*-(2-oxo-2-phenylethyl)methanesulfonamide (1e)**

Following general procedure **A**, using *N*-methylmethanesulfonamide (**S5**, 518 mg, 4.75 mmol, 1.00 equiv), 2-bromoacetophenone (945 mg, 4.75 mmol, 1.00 equiv) and K_2CO_3 (1.31 g, 9.49 mmol, 2.00 equiv) in acetonitrile (10 mL). The desired product was obtained after automated FC (CyH/EtOAc 100:0 to 50:50), and subsequent recrystallisation from hot EtOAc as a colourless solid (970 mg, 4.27 mmol, 90%).

M.P.: 104 – 105 °C. **IR (neat)**: $\tilde{\nu}$ = 3064 (w), 2933 (w), 1698 (m), 1597 (w), 1450 (w), 1410 (w), 1328 (s), 1225 (m), 1155 (s), 1024 (w), 969 (m), 946 (w), 917 (w), 817 (w), 779 (m), 757 (m), 690 (w), 647 (w), 520 (m), 485 (w). **^{1}H NMR (600 MHz, $CDCl_3$)**: δ = 7.95 – 7.90 (m, 2H), 7.63 (m, 1H), 7.53 – 7.47 (m, 2H), 4.83 (s, 2H), 3.06 (s, 3H), 3.04 (s, 3H). **^{13}C NMR (151 MHz, $CDCl_3$)**: δ = 194.8, 134.6, 134.3, 129.1, 128.0, 56.3, 38.9, 35.6. **HRMS (ESI)**: Calculated for $C_{10}H_{13}NO_3SNa$ [M+Na]$^+$: 250.0508, found 250.0508.

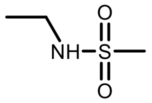

***N*-Ethylmethanesulfonamide (S6)**

Ethylamine hydrochloride (815 mg, 10.0 mmol, 2.00 equiv) and K_2CO_3 (1.52 g, 11.0 mmol, 2.20 equiv) were added to THF (5.00 mL) and methanol (0.25 mL) and stirred for 5 min, then methanesulfonyl chloride (573 mg, 387 µL, 5.00 mmol, 1.00 equiv) was added dropwise at 0 °C and the mixture was stirred at rt for 16 h. The solution was filtered, and the solvent was removed under reduced pressure. The crude product was purified via automated FC (CyH/EtOAc 100:00 to 20:80) and was obtained as a colourless oil (407 mg, 3.30 mmol, 66%).

IR (neat): $\tilde{\nu}$ = 3576 (w), 3284 (w), 2982 (w), 2937 (w), 2882 (w), 1454 (w), 1433 (w), 1412 (w), 1385 (w), 1310 (s), 1151 (s), 1063 (m), 978 (m), 939 (w), 860 (w), 785 (w), 761 (w), 648 (w), 630 (w), 617 (w), 600 (w), 521 (m), 488 (w), 476 (w), 466 (w), 453 (w). **^{1}H NMR (400 MHz, $CDCl_3$)**: δ = 4.43 (s, 1H), 3.29 – 3.11 (m, 2H), 2.96 (s, 3H), 1.23 (t, *J* = 7.2 Hz, 3H). **^{13}C NMR (101 MHz, $CDCl_3$)**: δ = 40.5, 38.4, 15.8. **HRMS (ESI)**:

Calculated for $C_3H_9NO_2SNa$ [M+H]$^+$: 146.0246, found 146.0242. Spectroscopic data was in agreement to those previously reported [11].

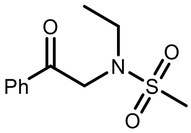

***N*-Ethyl-*N*-(2-oxo-2-phenylethyl)methanesulfonamide (1f)**

Following general procedure **A**, using 2-bromoacetophenone (651 mg, 3.27 mmol, 1.00 equiv), *N*-ethylmethanesulfonamide (**S6**, 403 mg, 3.27 mmol, 1.00 equiv) and K_2CO_3 (904 mg, 6.54 mmol, 2.00 equiv) in acetonitrile (6.50 mL). The desired product was obtained after automated FC (CyH/EtOAc 100:00 to 60:40) as a colourless solid (562 mg, 2.33 mmol, 71%).

M.P.: 80 – 82 °C. **IR (neat)**: $\tilde{\nu}$ = 2978 (w), 2935 (w), 1699 (m), 1597 (w), 1580 (w), 1450 (w), 1413 (w), 1325 (s), 1226 (m), 1210 (m), 1184 (w), 1148 (s), 1079 (w), 1044 (w), 961 (m), 897 (w), 775 (m), 755 (m), 690 (w), 648 (w), 515 (w), 498 (w). **^{1}H NMR (600 MHz, $CDCl_3$)**: δ = 7.95 – 7.92 (m, 2H), 7.65 – 7.61 (m, 1H), 7.53 – 7.48 (m, 2H), 4.85 (s, 2H), 3.41 (q, *J* = 7.2 Hz, 2H), 3.07 (s, 3H), 1.21 (t, *J* = 7.1 Hz, 3H). **^{13}C NMR (151 MHz, $CDCl_3$)**: δ = 195.2, 134.7, 134.3, 129.1, 128.0, 52.4, 42.9, 40.2, 14.1. **HRMS (ESI)**: Calculated for $C_{11}H_{16}NO_3S$ [M+H]$^+$: 242.0845, found 242.0842.

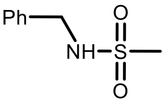

***N*-Benzylmethanesulfonamide (S7)**

N-Benzylmethanesulfonamide was prepared following a literature known protocol [12]. Benzylamine (1.18 g, 1.20 mL, 11.0 mmol, 1.10 equiv) was dissolved in dichloromethane (35.0 mL) and methanesulfonyl chloride (1.15 g, 774 µL, 10.0 mmol, 1.00 equiv) and triethylamine (2.53 g, 3.48 mL, 25.0 mmol, 2.50 equiv) were added. After 30 min, HCl (1 N, 10 mL) was added and the mixture was stirred for 10 min. The phases were separated, and the aqueous phase was extracted with dichloromethane (3 × 30 mL) and Et_2O (30 mL). The combined organic phases were washed with brine (30 mL) and water (30 mL) and were dried over $MgSO_4$. The solvent was removed under reduced pressure. The crude mixture was purified via automated FC (CyH/EtOAc 100:00 to 50:50) to obtain *N*-benzylmethanesulfonamide (1.63 g, 8.78 mmol, 88%) as a colourless solid.

IR (neat): $\tilde{\nu}$ = 3229 (m), 3034 (w), 3021 (w), 2933 (w), 2861 (w), 1494 (w), 1456 (m), 1437 (w), 1413 (w), 1379 (w), 1337 (w), 1204 (w), 1161 (m), 1133 (s), 1083 (w), 1083 (w), 1017 (w), 978 (w), 879 (w), 769 (m), 739 (m), 698 (m), 586 (w), 557 (w), 531 (m),

504 (m), 450 (w). **^{1}H NMR (400 MHz, $CDCl_3$)**: δ = 7.43 – 7.29 (m, 5H), 4.66 (s, 1H), 4.33 (d, J = 6.0 Hz, 2H), 2.87 (s, 3H). **^{13}C NMR (101 MHz, $CDCl_3$)**: δ = 136.7, 129.1, 128.3, 128.0, 47.4, 41.3. **HRMS (ESI)**: Calculated for $C_8H_{15}N_2O_2S$ $[M+NH_4]^+$: 203.0847, found 203.0849. Spectroscopic data was in agreement to those previously reported [13].

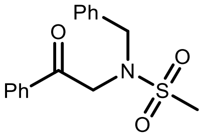

***N*-Benzyl-*N*-(2-oxo-2-phenylethyl)methanesulfonamide (1g)**

Following general procedure **A**, using *N*-benzylmethanesulfonamide (**S7**, 1.50 g, 8.12 mmol, 1.00 equiv), 2-bromoacetophenone (1.62 g, 8.12 mmol, 1.00 equiv) and K_2CO_3 (2.25 g, 16.2 mmol, 2.00 equiv) in acetonitrile (20.0 mL). The desired product was obtained after automated FC (CyH/EtOAc 90:10) as an off-white solid (1.48 g, 4.89 mmol, 60% yield).

IR (neat): $\tilde{\nu}$ = 3063 (w), 3031 (w), 1696 (m), 1597 (w), 1581 (w), 1495 (w), 1450 (w), 1409 (w), 1370 (w), 1328 (s), 1226 (m), 1146 (s), 1090 (w), 1062 (w), 1029 (w), 995 (w), 965 (w), 939 (m), 916 (w), 813 (w), 785 (m), 753 (m), 690 (m), 661 (w), 594 (w), 520 (m), 504 (m). **^{1}H NMR (400 MHz, $CDCl_3$)**: δ = 7.85 – 7.80 (m, 2H), 7.60 (ddt, J = 8.7, 7.0, 1.3 Hz, 1H), 7.50 – 7.40 (m, 2H), 7.37 – 7.28 (m, 5H), 4.66 (s, 2H), 4.56 (s, 2H), 3.17 (s, 3H). **^{13}C NMR (101 MHz, $CDCl_3$)**: δ = 195.3, 135.4, 134.7, 134.3, 129.1, 129.0, 128.7, 128.4, 128.0, 51.8, 51.1, 40.6. **HRMS (ESI)**: Calculated for $C_{16}H_{18}NO_3S$ $[M+H]^+$: 304.1002, found 304.1002. Spectroscopic data was in agreement to those previously reported.[14]

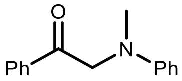

2-(Methyl(phenyl)amino)-1-phenylethan-1-one (1h)

Following general procedure **A** with minor alterations, using 2-bromoacetophenone (1.99 g, 10.0 mmol, 1.00 equiv), *N*-methylaniline (1.07 g, 1.08 mL, 10.0 mmol, 1.00 equiv) and K_2CO_3 (2.07 g, 15.0 mmol, 1.50 equiv) in acetonitrile (10 mL) at 70 °C for 14 h. The crude mixture was filtered, the solvent was removed under reduced pressure. EtOH (20 mL) was added and an off-white precipitate was formed. The solid was filtered, washed with cold EtOH (3 × 5 mL) and pentane (10 mL) and dried under reduced pressure. The product was obtained as a bright yellow solid (1.689 g, 7.50 mmol, 75%).

M.P.: 120 – 122 °C. **IR (neat)**: $\tilde{\nu}$ = 3062 (w), 2900 (w), 2168 (w), 1697 (s), 1599 (s), 1579 (w), 1506 (s), 1448 (w), 1371 (w), 1345 (w), 1254 (w), 1221 (s), 1118 (w), 987 (w), 972 (w), 943 (w), 918 (w), 748 (s), 690 (s), 666 (w), 572 (w), 542 (w), 519 (w), 494 (w), 482 (w), 447 (w), 439 (w). **^{1}H NMR (600 MHz, $CDCl_3$)**: δ = 8.02 – 7.97 (m, 2H), 7.64 – 7.58 (m, 1H), 7.53 – 7.47 (m, 2H), 7.25 – 7.18 (m, 2H), 6.73 (tt, *J* = 7.4, 1.0 Hz, 1H), 6.71 – 6.67 (m, 2H), 4.79 (s, 2H), 3.11 (s, 3H). **^{13}C NMR (151 MHz, $CDCl_3$)**: δ = 196.5, 149.2, 135.5, 133.7, 129.4, 129.0, 127.9, 117.4, 112.5, 59.1, 39.8. **HRMS (ESI)**: Calculated for $C_{15}H_{16}NO$ $[M+H]^+$: 226.1226, found: 226.1218. Spectroscopic data was in agreement to those previously reported [15].

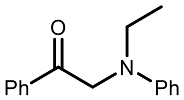

2-(Ethyl(phenyl)amino)-1-phenylethan-1-one (1i)

Following general procedure **A**, using 2-bromoacetophenone (995 mg, 5.00 mmol, 1.00 equiv), *N*-ethylaniline (606 mg, 629 µL, 5.00 mmol, 1.00 equiv) and K_2CO_3 (1.38 g, 10.0 mmol, 2.00 equiv) in acetonitrile (10.0 mL). The product was obtained after automated FC (CyH/EtOAc 100:00 to 70:30) and recrystallised from CyH/Toluene (80:20). The product was obtained as a yellow solid (651 mg, 2.72 mmol, 54%).

M.P.: 89 – 96 °C. **IR (neat)**: $\tilde{\nu}$ = 3062 (w), 2972 (w), 2927 (w), 1698 (s), 1598 (s), 1506 (s), 1449 (m), 1427 (w), 1391 (m), 1376 (w), 1353 (m), 1275 (w), 1247 (m), 1220 (s), 1195 (m), 1160 (w), 1129 (w), 1076 (w), 1041 (w), 1001 (w), 987 (w), 965 (m), 887 (w), 790 (w), 747 (s), 690 (s), 666 (w), 573 (w), 548 (w), 520 (w). **^{1}H NMR (400 MHz, $CDCl_3$)**: δ = 8.08 – 7.94 (m, 2H), 7.67 – 7.57 (m, 1H), 7.50 (ddd, *J* = 8.1, 6.6, 1.3 Hz, 2H), 7.24 – 7.15 (m, 2H), 6.74 – 6.68 (m, 1H), 6.65 (d, *J* = 8.2 Hz, 2H), 4.75 (s, 2H), 3.52 (q, *J* = 7.1 Hz, 2H), 1.23 (t, *J* = 7.1 Hz, 3H). **^{13}C NMR (101 MHz, $CDCl_3$)**: δ = 196.5, 148.0, 135.6, 133.7, 129.4, 129.0, 128.0, 117.1, 112.5, 57.0, 46.4, 12.6. **HRMS (ESI)**: Calculated for $C_{16}H_{18}NO$ $[M+H]^+$: 240.1383, found: 240.1382. Spectroscopic data was in agreement to those previously reported [15].

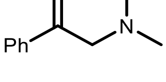

2-(Dimethylamino)-1-phenylethan-1-one (1j)

Following general procedure **A** with minor alterations, using 2-bromoacetophenone (2.00 g, 10.0 mmol, 1.00 equiv), K_2CO_3 (2.78 g, 20.1 mmol, 2.00 equiv) in acetonitrile (50.0 mL). Dimethylamine (5.60 M in ethanol, 505 mg, 2.00 mL, 11.2 mmol, 1.06 equiv) was added dropwise at 0 °C. The product was obtained after bulb-to-bulb

distillation (96 °C, 0.95 mbar) as a yellow oil (340 mg, 2.08 mmol, 20%). The product is sensitive to air and light exposure.

IR (neat): $\tilde{\nu}$ = 3062 (w), 2947 (w), 2826 (w), 2775 (w), 1696 (s), 1598 (m), 1579 (w), 1450 (m), 1376 (m), 1314 (w), 1286 (w), 1228 (s), 1171 (w), 1068 (w), 1024 (w), 968 (m), 860 (w), 757 (m), 718 (s), 690 (s), 673 (m), 580 (w), 498 (w), 459 (w), 406 (w). **^{1}H NMR (300 MHz, $CDCl_3$)**: δ = 8.05 – 7.95 (m, 2H), 7.62 – 7.52 (m, 1H), 7.46 (ddt, *J* = 8.2, 6.6, 1.2 Hz, 2H), 3.77 (s, 2H), 2.39 (s, 6H). **^{13}C NMR (101 MHz, $CDCl_3$)**: δ = 196.8, 136.0, 133.3, 128.7, 128.2, 65.5, 45.8. **HRMS (ESI)**: Calculated for $C_{10}H_{14}NO$ $[M+H]^+$: 164.1070, Found: 164.1069. Spectroscopic data was in agreement to those previously reported [16].

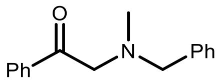

2-(Benzyl(methyl)amino)-1-phenylethan-1-one (1k)

Following general procedure **A** with minor alterations, using 2-bromoacetophenone (2.00 g, 10.0 mmol, 1.00 equiv) and K_2CO_3 (2.78 g, 20.1 mmol, 2.00 equiv) in acetonitrile (50.0 mL). *N*-Methyl-1-phenylmethanamine (1.32 g, 1.40 mL, 10.6 mmol, 1.05 equiv) was added dropwise over 15 min at 0 °C. The desired product was obtained after bulb-to-bulb distillation (190 °C 0.75 mbar) as a yellow-orange oil (1.29 g, 5.38 mmol, 54%).

IR (neat): $\tilde{\nu}$ = 3062 (w), 3028 (w), 2788 (w), 1682 (s), 1598 (m), 1580 (w), 1494 (w), 1449 (m), 1369 (m), 1286 (m), 1258 (w), 1220 (m), 1180 (w), 1124 (w), 1076 (w), 1026 (w), 967 (m), 870 (w), 742 (s), 691 (s), 589 (w), 484 (w), 439 (w). **^{1}H NMR (300 MHz, $CDCl_3$)**: δ = 8.04 – 7.89 (m, 2H), 7.60 – 7.52 (m, 1H), 7.48 – 7.39 (m, 2H), 7.39 – 7.19 (m, 5H), 3.81 (s, 2H), 3.70 (s, 2H), 2.39 (s, 3H). **^{13}C NMR (101 MHz, $CDCl_3$)**: δ = 197.9, 138.3, 136.2, 133.3, 129.4, 128.6, 128.5, 128.4, 128.4, 127.4, 63.2, 62.1, 42.9. **HRMS (ESI)**: Calculated for $C_{16}H_{18}NO$ $[M+H]^+$: 240.1383, Found: 240.1384. Spectroscopic data was in agreement to those previously reported [17].

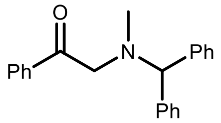

2-(Benzhydryl(methyl)amino)-1-phenylethan-1-one (1l)

Following general procedure **A** with minor alterations, using 2-bromoacetophenone (2.00 g, 10.0 mmol, 1.00 equiv) and K_2CO_3 (2.78 g, 20.1 mmol, 2.00 equiv) in acetonitrile (50.0 mL). *N*-Methyl-1,1-diphenylmethanamine (2.08 g, 10.6 mmol, 1.05 equiv) was added at 0 °C and the reaction stirred at rt for 23 h, then at 55 °C for

2 h. The desired product was obtained after automated FC (CyH/EtOAc 100:00 to 80:20) as an off-white solid (2.50 g, 7.93 mmol, 79%).

M.P.: 75 °C – 78 °C. **IR (neat)**: $\tilde{\nu}$ = 3062 (w), 3028 (w), 2925 (w), 2788 (w), 1689 (s), 1598 (m), 1580 (w), 1492 (m), 1450 (s), 1280 (m), 1219 (m), 1181 (w), 1119 (w), 1027 (w), 985 (w), 749 (s), 705 (s), 560 (w), 457 (w). **^{1}H NMR (300 MHz, $CDCl_3$)**: δ = 7.96 – 7.83 (m, 2H), 7.58 – 7.51 (m, 1H), 7.50 – 7.46 (m, 4H), 7.45 – 7.40 (m, 2H), 7.34 – 7.26 (m, 4H), 7.25 – 7.14 (m, 2H), 4.80 (s, 1H), 3.84 (s, 2H), 2.35 (s, 3H). **^{13}C NMR (101 MHz, $CDCl_3$)**: δ = 198.6, 142.5, 136.4, 133.1, 128.7, 128.5, 128.3, 128.2, 127.3, 74.8, 61.8, 41.4. **HRMS (ESI)**: Calculated for $C_{22}H_{22}NO$ $[M+H]^+$: 316.1696, Found: 316.1687.

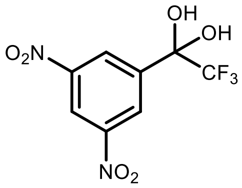

3'5'-Dinitro-2,2,2-trifluoroacetophenone monohydrate (S8)

3'5'-Dinitro-2,2,2-trifluoroacetophenone monohydrate was prepared according to a literature known procedure [18]. 2,2,2-Trifluoroacetophenone (1.98 g, 11.4 mmol, 1.00 equiv) was dissolved in H_2SO_4 conc. (50 mL) and HNO_3 aq. (65%, 15 mL) was added slowly at 0 °C and stirred for 30 min before the reaction mixture was heated to 130 °C. After 4 h the reaction mixture was cooled to rt before another portion of HNO_3 aq. (65%, 15 mL) was added. The solution was refluxed at 130 °C for 3 d before it was poured on ice. The aqueous phase was extracted with Et_2O (3 × 25 mL) and the organic phase was carefully washed with $NaHCO_3$ sat. until no gas evolution was observed. The organic phase was dried over K_2CO_3 and the solvent was removed in vacuo. The crude product was recrystallised from H_2O to yield the hydrate as colourless crystals (1.65 g, 6.24 mmol, 55%).

^{1}H NMR (400 MHz, DMSO-d_6): δ = 8.91 (t, J = 2.1 Hz, 1H), 8.67 (d, J = 2.1 Hz, 2H), 8.45 (s, 2H). **^{13}C NMR (101 MHz, DMSO-d_6):** δ = 148.0, 142.7, 127.4, 122.8 (q, J = 289.0 Hz), 119.7, 91.8 (q, J = 32.0 Hz). **^{19}F NMR (376 MHz, DMSO-d_6):** δ = – 82.6 (*s*, C*F*$_3$). Spectroscopic data was in agreement to those previously reported [18].

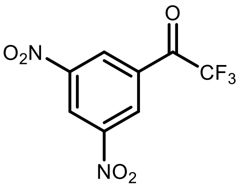

3'5'-Dinitro-2,2,2-trifluoroacetophenone (7)

3'5'-Dinitro-2,2,2-trifluoroacetophenone monohydrate (**S8**) was refluxed with toluene in a Dean-Stark apparatus for 4 h. The water-free product was obtained as a light yellow solid (quantitative conversion of the hydrate).

^{1}H NMR (400 MHz, $CDCl_3$): δ = 9.37 (t, J = 2.1 Hz, 1H), 9.19 (dd, J = 2.0, 0.9 Hz, 2H).**^{13}C NMR (101 MHz, $CDCl_3$):** δ = 177.4 (q, J = 38.0 Hz), 149.3, 132.6, 129.6 (q, J = 2.2 Hz), 124.5, 116.0 (q, J = 290.2 Hz, CF_3). **^{19}F NMR (377 MHz, $CDCl_3$):** δ = – 71.8 (*s*, CF_3). Spectroscopic data was in agreement to those previously reported [18].

3. Substrate scope photocyclization

The investigation of the effect of structural variations on the photoreaction (Table 1, Discussion) was conducted in CD_3CN to achieve comparative results after 4 h of irradiation. For the isolation of the respective substances longer irradiation times were needed, and some products precipitated from MeCN in the scale-up after 6 h. Therefore, some reactions were performed in DMF, as it showed a better solubility for some products and is also known to efficiently facilitate the Norrish–Yang reaction [2].

General procedure **B** for the synthesis of 3-phenylazetidinols from α-aminoacetophenones:

3-Phenylazetidinols were prepared following a literature known protocol [2]. An α-aminoacetophenone was dissolved in acetonitrile (50 mM) and was irradiated using a Luzchem Photoreactor equipped with 10 8 Watt LZC-UVA (355 nm) lamps at rt. The solvent was removed under reduced pressure and the desired product was isolated after FC, automated FC, or RP-MPLC.

General procedure **C** for the preparation of 3-phenylazetidinols from α-aminoacetophenones:

An α-aminoacetophenone was dissolved in DMF (50 mM) and was irradiated using a Luzchem Photoreactor equipped with 10 8 Watt LZC-UVA (355 nm) lamps at rt. EtOAc (10 mL) was added and the mixture was washed with aq. LiCl solution (5%, 3 × 10 mL) to remove DMF and the organic phase was dried over $MgSO_4$. The solvent was removed under reduced pressure and the desired product was isolated after FC, automated FC, or RP-MPLC.

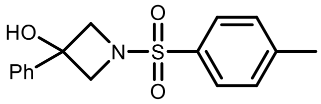

3-Phenyl-1-tosylazetidin-3-ol (3a)

3-Phenyl-1-tosylazetidin-3-ol was prepared following a literature known protocol.[2] *N*,4-Dimethyl-*N*-(2-oxo-2-phenylethyl)benzenesulfonamide (**1a**, 346 mg, 1.14 mmol, 1.00 equiv) was dissolved in acetonitrile (22.0 mL) and was irradiated using a Luzchem Photoreactor equipped with 10 8 Watt LZC-UVA (355 nm) lamps for 12 h at rt. The solvent was removed under reduced pressure. The product was obtained after automated FC (CyH/EtOAc 100:0 to 60:40) as a colourless solid (286 mg, 0.94 mmol, 83%).

^{1}H NMR (600 MHz, DMSO-*d*$_6$): δ = 7.78 – 7.73 (m, 2H), 7.57 – 7.51 (m, 2H), 7.32 – 7.22 (m, 3H), 7.19 – 7.15 (m, 2H), 6.35 (s, 1H), 3.93 – 3.89 (m, 2H), 3.83 – 3.80 (m, 2H), 2.47 (s, 3H).**^{13}C NMR (151 MHz, DMSO-*d*$_6$)**: δ = 144.2, 143.7, 130.4, 130.1, 128.5, 128.2, 127.4, 124.7, 68.6, 65.4, 21.1. Spectroscopic data was in agreement to those previously reported.[2]

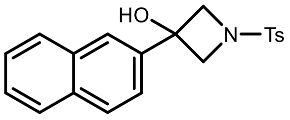

3-(Naphthalen-2-yl)-1-tosylazetidin-3-ol (3b)

Following general procedure **B**, using 3-(naphthalen-2-yl)-1-tosylazetidin-3-ol (**1b**, 303 mg, 0.86 mmol) in DMF (40 mL) for 8 h. The desired product was obtained after RP-MPLC (MeCN/H_2O 50:50) as a colorless solid (51 mg, 0.14 mmol, 16%).

M.P.: decomposition above 165 °C. **IR (neat)**: $\tilde{\nu}$ = 3401 (w), 2924 (w), 1598 (w), 1448 (w), 1337 (m), 1160 (s), 1092 (m), 899 (w), 816 (m), 679 (m), 622 (w), 595 (w), 549 (m), 474 (w). **^{1}H NMR (600 MHz, DMSO-*d*$_6$)**: δ = 7.88 (dd, *J* = 7.4, 1.8 Hz, 1H), 7.83 (d, *J* = 8.6 Hz, 1H), 7.81 – 7.76 (m, 3H), 7.66 (d, *J* = 2.2 Hz, 1H), 7.56 – 7.43 (m, 4H), 7.30 (dd, *J* = 8.6, 1.9 Hz, 1H), 6.52 (s, 1H), 4.05 (d, *J* = 9.0 Hz, 2H), 3.90 (d, *J* = 9.0 Hz, 2H), 2.43 (s, 3H). **^{13}C NMR (151 MHz, DMSO-*d*$_6$)**: δ = 144.3, 140.9, 132.4, 132.1, 130.4, 130.1, 128.5, 128.0, 128.0, 127.4, 126.4, 126.2, 123.3, 123.2, 68.9, 65.2, 21.1. **HRMS (ESI)**: Calculated for $C_{20}H_{20}NO_3S$ [M+H]$^+$: 354.1158 found 354.1151.

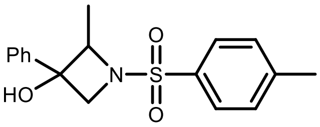

2-Methyl-3-phenyl-1-tosylazetidin-3-ol (3c)

Following general procedure **B**, using *N*-ethyl-4-methyl-*N*-phenacylbenzene-sulfonamide (**1c**, 95.2 mg, 300 µmol, 1.00 equiv) in MeCN (6.00 mL) for 8 h. The desired product was obtained after automated FC (CyH/Et_2O 80:20) as a mixture of both diastereomers (dr = 70:30 for isolated material, 41.0 mg, 129 µmol, 43% yield) as a colourless solid.

Diastereomers are assigned from the isolated mixture according to literature:

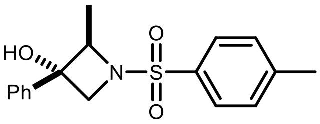

Major-(3c), (2*R*,3*S*)-2-methyl-3-phenyl-1-tosylazetidin-3-ol:

^{1}H NMR (600 MHz, $CDCl_3$): δ = 7.80 – 7.75 (m, 2H), 7.56 – 7.51 (m, 2H), 7.46 – 7.33 (m, 5H), 4.27 (dd, *J* = 8.5, 1.0 Hz, 1H), 4.00 – 3.93 (m, 1H), 3.65 (d, *J* = 8.4 Hz, 1H), 2.48 (s, 3H), 2.14 (s, 1H), 0.90 (d, *J* = 6.5 Hz, 3H). **^{13}C NMR (151 MHz, $CDCl_3$)**: δ = 144.5, 139.1, 131.0, 130.0, 128.7, 128.7, 128.5, 125.8, 74.1, 71.9, 62.9, 21.8, 16.7. Spectroscopic data was in agreement to those previously reported.[19, 20]

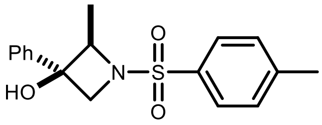

Minor-(3c) (2*R*,3*R*)-2-methyl-3-phenyl-1-tosylazetidin-3-ol:

^{1}H NMR (600 MHz, $CDCl_3$): δ = 7.40 – 7.34 (m, 4H), 7.22 (dd, *J* = 5.1, 2.0 Hz, 3H), 6.98 – 6.93 (m, 2H), 4.19 (q, *J* = 6.4 Hz, 1H), 4.00 – 3.93 (m, 1H), 3.88 (dd, *J* = 9.1, 1.0 Hz, 1H), 2.48 (s, 3H), 2.39 (s, 1H), 1.42 (d, *J* = 6.4 Hz, 3H). **^{13}C NMR (151 MHz, $CDCl_3$)**: δ = 144.4, 141.6, 131.9, 130.0, 128.7, 128.6, 128.1, 124.8, 73.3, 70.3, 63.0, 21.8, 14.5. Spectroscopic data was in agreement to those previously reported. [19]

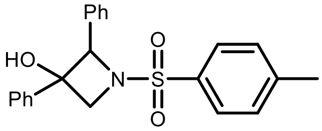

2,3-Diphenyl-1-tosylazetidin-3-ol (3d)

Following general procedure **C**, using *N*-benzyl-4-methyl-*N*-phenacyl-benzenesulfonamide (**1d**, 114 mg, 300 µmol, 1.00 equiv) in DMF (6.00 mL) for 12 h. The desired product was obtained after automated FC (CyH/EtOAc 100:0 to 70:30) as two diastereomers (dr = 49:51 for isolated material) minor-(**3d**) (20.0 mg, 53 µmol, 18%) and major-(**3d**) (21.0 mg, 55 µmol, 18%), both colourless solids.

Diastereomers are assigned from the mixture according to literature:

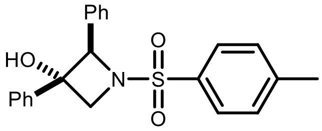

Major-(3d) (2*R*,3*S*)-2,3-diphenyl-1-tosylazetidin-3-ol:

M.P.: 211 – 215 °C. **IR (neat)**: $\tilde{\nu}$ = 3445 (w), 2954 (w), 2918 (m), 2866 (w), 2850 (w), 1450 (w), 1333 (m), 1151 (s), 1087 (w), 823 (w), 778 (w), 697 (m), 677 (m), 600 (m), 550 (w), 536 (w), 525 (w), 513 (w), 513 (w), 457 (m). **^{1}H NMR (400 MHz, DMSO-d_6)**: δ = 7.76 – 7.66 (m, 2H), 7.54 (d, *J* = 7.9 Hz, 2H), 7.24 (dt, *J* = 6.6, 1.4 Hz, 2H), 7.14 – 7.08 (m, 2H), 7.08 – 6.98 (m, 6H), 6.51 (s, 1H), 4.89 (s, 1H), 4.41 – 4.24 (m, 1H), 3.73 (d, *J* = 8.2 Hz, 1H), 2.47 (s, 3H). **^{13}C NMR (101 MHz, DMSO-d_6)**: δ = 144.3, 139.9, 135.9, 130.3, 130.1, 128.5, 127.4, 127.2, 127.1, 126.5, 126.1, 78.3, 74.7, 62.0, 21.2.[b] **HRMS (ESI)**: Calculated for $C_{22}H_{22}NO_3S$ $[M+H]^+$: 380.1315, found: 380.1312. Spectroscopic data (^{1}H NMR) was in agreement to those previously reported [14].

[b] Signals missing due to signal overlapping.

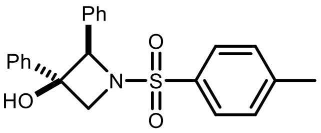

Minor-(3d) (2*R*,3*R*)-2,3-diphenyl-1-tosylazetidin-3-ol**:**

M.P.: 156 – 160 °C. **IR (neat)**: $\tilde{\nu}$ = 3464 (w), 2925 (w), 1698 (w), 1598 (w), 1496 (w), 1450 (w), 1330 (m), 1224 (m), 1158 (s), 1092 (m), 1028 (w), 967 (w), 914 (w), 818 (w), 778 (m), 753 (m), 698 (s), 671 (s), 651 (w), 599 (s), 550 (s), 485 (w). **^{1}H NMR (400 MHz, DMSO-*d*$_6$)**: δ = 7.78 – 7.71 (m, 2H), 7.58 – 7.50 (m, 2H), 7.34 (d, *J* = 3.4 Hz, 5H), 7.26 – 7.13 (m, 3H), 6.91 – 6.69 (m, 2H), 6.07 (s, 1H), 4.83 (s, 1H), 4.02 (d, *J* = 9.2 Hz, 1H), 3.94 (dd, *J* = 9.2, 1.0 Hz, 1H), 2.48 (s, 3H). **^{13}C NMR (101 MHz, DMSO-*d*$_6$)**: δ = 144.5, 143.3, 135.3, 130.4, 130.1, 128.5, 128.1, 127.9, 127.7, 127.7, 127.4, 124.8, 76.9, 72.6, 62.5, 21.1. **HRMS (ESI)**: Calculated for $C_{22}H_{22}NO_3S$ [M+H]$^+$: 380.1315, found: 380.1310. Spectroscopic data (^{1}H NMR) was in agreement to those previously reported [14].

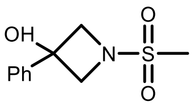

1-(Methylsulfonyl)-3-phenylazetidin-3-ol (3e)

Following general procedure **B**, using *N*-methyl-*N*-phenacylmethanesulfonamide (**1e**, 455 mg, 2.00 mmol, 1.00 equiv) in MeCN (40 mL) for 8 h. The desired product was obtained after automated FC (CyH/EtOAc 50:50 to 15:85) as obtained as a colourless solid (269 mg, 1.18 mmol, 59%).

M.P.: 138 – 142 °C. **IR (neat)**: $\tilde{\nu}$ = 3461 (w), 2935 (w), 1449 (w), 1325 (s), 1146 (s), 1078 (m), 1029 (w), 963 (w), 761 (m), 701 (m), 602 (w), 555 (w), 523 (m). **^{1}H NMR (400 MHz, CDCl$_3$)**: δ = 7.57 – 7.52 (m, 2H), 7.47 – 7.40 (m, 2H), 7.39 – 7.33 (m, 1H), 4.35 – 4.26 (m, 2H), 4.23 – 4.16 (m, 2H), 2.96 (s, 3H), 2.83 – 2.72 (m, 1H). **^{13}C NMR (101 MHz, CDCl$_3$)**: δ = 142.1, 129.0, 128.6, 124.7, 70.6, 64.7, 36.9. **HRMS (ESI)**: Calculated for $C_{10}H_{14}NO_3S$ [M+H]$^+$: 228,0689, found 228,0689.

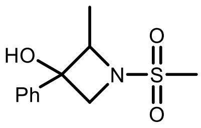

2-Methyl-1-(methylsulfonyl)-3-phenylazetidin-3-ol (3f)

Following general procedure **C**, using *N*-ethyl-*N*-phenacylmethanesulfonamide (**1f**, 90.0 mg, 373 µmol, 1.00 equiv) in DMF (10 mL) for 8 h. The desired product was obtained after automated FC (CyH/EtOAc 100:00 to 60:40) as two diastereomers (dr = 40:60 for isolated material) minor-(**3f**) (8.00 mg, 33.2 µmol, 9%) and major-(**3f**)

(12.0 mg, 49.7 µmol, 13%), both a colourless wax. The diastereomers were assigned according to **(3c)**.

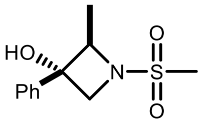

Major-(3f) (2*S*,3*R*)-2-methyl-1-(methylsulfonyl)-3-phenylazetidin-3-ol:

IR (neat): $\tilde{\nu}$ = 3466 (w), 3027 (w), 3012 (w), 2985 (w), 2932 (w), 2890 (w), 1621 (w), 1497 (w), 1470 (w), 1451 (w), 1412 (w), 1377 (w), 1322 (s), 1243 (w), 1144 (s), 1079 (m), 1031 (w), 989 (m), 989 (m), 971 (w), 916 (w), 866 (w), 794 (m), 765 (m), 743 (w), 701 (m), 701 (m), 598 (w), 548 (w), 526 (m), 506 (w). **^{1}H NMR (400 MHz, $CDCl_3$)**: δ = 7.58 – 7.51 (m, 2H), 7.48 – 7.41 (m, 2H), 7.40 – 7.32 (m, 1H), 4.46 (qd, *J* = 6.5, 0.9 Hz, 1H), 4.29 (dd, *J* = 8.6, 0.9 Hz, 1H), 4.09 (d, *J* = 8.6 Hz, 1H), 2.98 (s, 3H), 2.77 (br s, 1H), 0.92 (d, *J* = 6.5 Hz, 3H). **^{13}C NMR (101 MHz, $CDCl_3$)**: δ = 139.1, 128.7, 128.5, 125.9, 73.7, 71.6, 62.5, 36.9, 17.1. **HRMS (ESI)**: Calculated for $C_{11}H_{16}NO_3S$ $[M+H]^+$: 242.0845, found 242.0842.

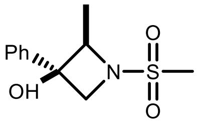

Minor-(3f) (2*S*,3*S*)-2-methyl-1-(methylsulfonyl)-3-phenylazetidin-3-ol**:**

IR (neat): $\tilde{\nu}$ = 3468 (w), 3029 (w), 2979 (w), 2932 (w), 1604 (w), 1497 (w), 1449 (w), 1378 (w), 1315 (s), 1180 (m), 1144 (s), 1076 (m), 1032 (w), 991 (m), 960 (m), 913 (w), 897 (w), 799 (m), 753 (m), 700 (s), 624 (m), 583 (m), 518 (m), 484 (m). **^{1}H NMR (400 MHz, $CDCl_3$)**: δ = 7.46 – 7.38 (m, 4H), 7.37 – 7.29 (m, 1H), 4.67 (qd, *J* = 6.4, 1.0 Hz, 1H), 4.38 (d, *J* = 8.7 Hz, 1H), 3.87 (dd, *J* = 8.7, 1.0 Hz, 1H), 2.92 (s, 3H), 2.44 (br s, 1H), 1.48 (d, *J* = 6.4 Hz, 3H). **^{13}C NMR (101 MHz, $CDCl_3$)**: δ =141.9, 129.0, 128.5, 125.1, 73.0, 68.7, 61.8, 38.9, 14.7. **HRMS (ESI)**: Calculated for $C_{11}H_{16}NO_3S$ $[M+H]^+$: 242.0845, found 242.0841.

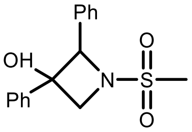

1-(Methylsulfonyl)-2,3-diphenylazetidin-3-ol (3g)

Following general procedure **C**, using *N*-benzyl-*N*-(2-oxo-2-phenylethyl)methane-sulfonamide (**1g**, 96.0 mg, 317 µmol, 1.00 equiv) in DMF (12.7 mL) for 8 h. The desired product was obtained after automated FC (CyH/EtOAc 50:50) as two diastereomers (dr = 44:56) minor-(**3g**) (20.0 mg, 65.9 µmol, 21%) and major-(**3g**) (26.0 mg, 85.7 µmol, 27%) as colourless resigns. The diastereomers were not assigned.

Major-(3g):

IR (neat): $\tilde{\nu}$ = 3468 (w), 3063 (w), 3030 (w), 2931 (w), 1496 (w), 1450 (w), 1409 (w), 1320 (s), 1227 (w), 1193 (w), 1146 (s), 1079 (w), 1059 (w), 1027 (w), 965 (m), 913 (w), 870 (w), 826 (w), 784 (m), 756 (w), 732 (w), 698 (s), 644 (w), 625 (w), 579 (w), 529 (m), 512 (w), 472 (w), 462 (w), 446 (w), 421 (w), 410 (w). **^{1}H NMR (400 MHz, $CDCl_3$)**: δ =7.26 – 7.21 (m, 2H), 7.20 – 7.13 (m, 3H), 7.09 (q, *J* = 3.5 Hz, 3H), 7.07 – 7.02 (m, 2H), 5.43 (s, 1H), 4.45 (d, *J* = 8.7 Hz, 1H), 4.32 (d, *J* = 8.7 Hz, 1H), 3.01 (s, 1H), 2.84 (s, 3H). **^{13}C NMR (101 MHz, $CDCl_3$)**: δ = 138.3, 135.2, 128.2, 128.1, 127.1, 126.3, 78.2, 76.0, 59.8, 38.8.[c] **HRMS (ESI)**: Calculated for **$C_{16}H_{18}NO_3S$** [M+H]+: 304.1002, found: 304.0999. Spectroscopic data (^{1}H NMR) was in agreement to those previously reported [14].

Minor-(3g):

IR (neat): $\tilde{\nu}$ = 3712 (w), 3652 (w), 3465 (w), 3032 (w), 2933 (w), 2349 (w), 2262 (w), 2222 (w), 2184 (w), 2168 (w), 2069 (w), 2038 (w), 1991 (w), 1969 (w), 1727 (w), 1496 (w), 1450 (w), 1313 (s), 1144 (s), 1062 (m), 1027 (m), 963 (m), 908 (w), 828 (w), 790 (m), 751 (m), 732 (m), 698 (s), 651 (w), 635 (w), 582 (m), 519 (m), 466 (w), 456 (w), 442 (w), 418 (w). **^{1}H NMR (400 MHz, $CDCl_3$)**: δ = 7.52 (d, *J* = 7.7 Hz, 2H), 7.48 – 7.31 (m, 8H), 5.65 (s, 1H), 4.63 (d, *J* = 8.7 Hz, 1H), 3.96 (d, *J* = 8.8 Hz, 1H), 2.86 (d, *J* = 1.9 Hz, 3H), 2.09 (s, 1H). **^{13}C NMR (101 MHz, $CDCl_3$)**: δ = 142.1, 133.6, 129.4, 129.1, 129.0, 128.5, 127.9, 125.3, 75.1, 73.3, 60.9, 40.1. **HRMS (ESI)**: Calculated for $C_{16}H_{18}NO_3S$ [M+H]+: 304.1002, found: 304.0999. Spectroscopic data (^{1}H NMR) was in agreement to those previously reported [14].

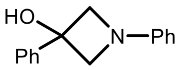

1,3-Diphenylazetidin-3-ol (3h)

Following general procedure **B**, using 2-(methyl(phenyl)amino)-1-phenylethan-1-one (**1h**, 135.2, 600 µmol, 1.00 equiv) in THF for 8 h. The desired product was obtained after automated FC (CyH/EtOAc 90:10 to 80:20.) as an off-white solid (75.0 mg, 333 µmol, 55%).

M.P.: 80 – 84 °C. **IR (neat)**: $\tilde{\nu}$ = 3370 (w), 3060 (w), 3031 (w), 2929 (w), 2851 (w), 1599 (s), 1502 (s), 1472 (m), 1449 (w), 1343 (m), 1261 (w), 1228 (w), 1176 (w), 1155 (w), 1126 (w), 1061 (w), 1028 (w), 912 (w), 874 (w), 828 (w), 752 (s), 693 (s), 668 (w), 562 (w), 516 (w). **^{1}H NMR (400 MHz, $CDCl_3$)**: δ = 7.63 – 7.57 (m, 2H), 7.41 (dd, *J* = 8.4, 6.8 Hz, 2H), 7.36 – 7.30 (m, 1H), 7.29 – 7.22 (m, 2H), 6.81 (tt, *J* = 7.3, 1.1 Hz, 1H), 6.58 – 6.50 (m, 2H), 4.26 (d, *J* = 7.8 Hz, 2H), 4.09 (d, *J* = 7.8 Hz, 2H), 2.62 (s, 1H). **^{13}C NMR (101 MHz, $CDCl_3$)**: δ = 151.3, 143.6, 129.1, 128.7, 127.9, 124.8, 118.3,

[c] Signals missing due to signal overlapping.

112.2, 72.5, 67.2. **HRMS (ESI)**: Calculated for $C_{15}H_{16}NO$ $[M+H]^+$: 226.1226, found: 226.1218. Spectroscopic data was in agreement to those previously reported [15].

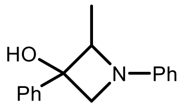

2-Methyl-1,3-diphenylazetidin-3-ol (3i)

Following general procedure **C**, using 2-(*N*-ethylanilino)-1-phenylethanone (**1i**, 145 mg, 606 µmol, 1.00 equiv) in DMF (12.1 mL) for 24 h. The desired product was obtained after automated FC (CyH/EtOAc 100:00 to 70:30) as two diastereomers (dr = 44:56) minor-(**3i**) (14.0 mg, 58.5 µmol, 10%) and major-(**3i**) (18.0 mg, 75.2 µmol, 12%) as colourless waxes. The diastereomers were not assigned.

Major-(3i):

IR (neat): $\tilde{\nu}$ = 3416 (w), 3088 (w), 3058 (w), 3027 (w), 2977 (w), 2930 (w), 2865 (w), 1682 (w), 1599 (s), 1498 (s), 1447 (m), 1374 (m), 1323 (m), 1260 (w), 1178 (m), 1142 (m), 1064 (m), 1028 (m), 909 (w), 752 (s), 699 (s), 648 (w), 617 (w), 585 (w), 507 (w). **^{1}H NMR (400 MHz, CDCl$_3$)**: δ = 7.48 – 7.41 (m, 2H), 7.40 – 7.34 (m, 2H), 7.32 – 7.21 (m, 3H), 6.81 (tt, J = 7.4, 1.1 Hz, 1H), 6.61 (dtd, J = 8.8, 2.1, 1.2 Hz, 2H), 4.38 – 4.30 (m, 1H), 4.19 – 4.14 (m, 1H), 4.08 (d, J = 8.3 Hz, 1H), 3.65 (br s, 1H), 1.80 – 1.47 (m, 3H). **^{13}C NMR (101 MHz, CDCl$_3$)**: δ = 151.5, 143.3, 129.2, 128.7, 127.9, 125.2, 118.5, 112.8, 74.6, 70.5, 65.6, 15.5. **HRMS (ESI)**: Calculated for $C_{16}H_{18}NO$ $[M+H]^+$: 240.1482, found 240.1382.

Minor-(3i):

IR (neat): $\tilde{\nu}$ = 3401 (w), 3060 (w), 3031 (w), 2925 (m), 2855 (w), 1599 (s), 1499 (s), 1470 (w), 1451 (m), 1377 (w), 1329 (s), 1179 (w), 1147 (m), 1100 (w), 1073 (m), 1032 (w), 1007 (w), 876 (w), 781 (w), 754 (s), 694 (s), 596 (w), 523 (w), 463 (w), 427 (w), 411 (w). **^{1}H NMR (400 MHz, CDCl$_3$)**: δ = 7.68 – 7.61 (m, 2H), 7.48 – 7.40 (m, 2H), 7.39 – 7.31 (m, 1H), 7.29 – 7.20 (m, 4H), 6.82 (tt, J = 7.3, 1.1 Hz, 1H), 6.71 – 6.60 (m, 2H), 4.52 (dd, J = 7.9, 1.0 Hz, 1H), 4.21 (q, J = 6.4 Hz, 1H), 3.83 (d, J = 7.9 Hz, 1H), 2.26 (br s, 1H), 1.02 (d, J = 6.4 Hz, 3H). **^{13}C NMR (101 MHz, CDCl$_3$)**: δ = 152.3, 140.8, 129.2, 128.4, 127.8, 126.1, 118.8, 113.1, 75.5, 72.6, 65.6, 18.2. **HRMS (ESI)**: Calculated for $C_{16}H_{18}NO$ $[M+H]^+$: 240.1482, found 240.1382. Spectroscopic data was in agreement to those previously reported [15].

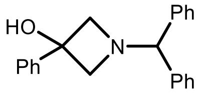

1-Benzhydryl-3-phenylazetidin-3-ol (3l)

Following general procedure **B**, using 2-(benzhydryl(methyl)amino)-1-phenylethan-1-one (284 mg, 900 µmol, 1.00 equiv) in acetonitrile (45 mL) for 12 h at 22 °C. The solvent was removed under reduced pressure and the crude product was purified via

automated FC (CyH/EtOAc 100:00 to 50:50). The product was obtained as a colorless solid (37.0 mg, 117 µmol, 13%).

M.P.: 99 – 102 °C. **IR (neat)**: $\tilde{\nu}$ = 3363 (w), 3084 (w), 3060 (w), 3027 (w), 2944 (w), 2836 (w), 1600 (w), 1493 (w), 1450 (m), 1390 (w), 1344 (w), 1308 (w), 1267 (w), 1212 (m), 1179 (w), 1157 (w), 1076 (w), 1064 (w), 1028 (w), 913 (w), 890 (w), 858 (w), 754 (m), 741 (m), 698 (s), 638 (w), 623 (w), 614 (w), 550 (w), 472 (w). **^{1}H NMR (400 MHz, $CDCl_3$)**: δ = 7.71 – 7.57 (m, 2H), 7.51 – 7.38 (m, 6H), 7.36 – 7.25 (m, 5H), 7.24 – 7.15 (m, 2H), 4.49 (s, 1H), 3.67 – 3.56 (m, 2H), 3.48 – 3.34 (m, 2H), 2.57 (s, 1H). **^{13}C NMR (101 MHz, $CDCl_3$)**: δ = 144.3, 142.3, 128.6, 128.6, 127.7, 127.6, 127.3, 125.0, 78.2, 71.6, 67.7. **HRMS (ESI)**: Calculated for $C_{22}H_{22}NO$ $[M+H]^+$ 316.1696, found: 316.1691. Spectroscopic data was in agreement to those previously reported [21].

4. Ring opening

4.1. Optimisation of dioxolane formation

4.2. Unsuccessful attempts for Ts-protected azetidinol (3a)

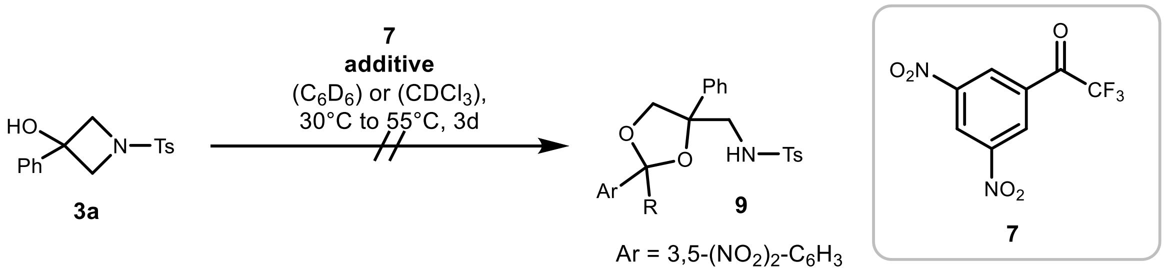

To achieve a successful formation of a dioxolane (see discussion, Scheme 3), several azetidinols and ketones were subjected to several Lewis acids (**S8** to **S13**), Brønsted acids (**S14** to **S17**) and base **S18**, without success:

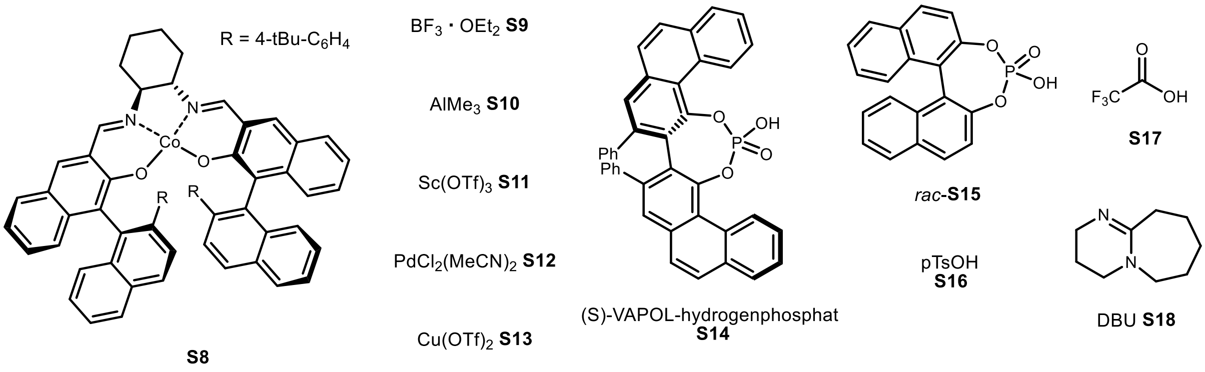

4.3. Successful opening for benzhydryl-protected azetidinol (3l)

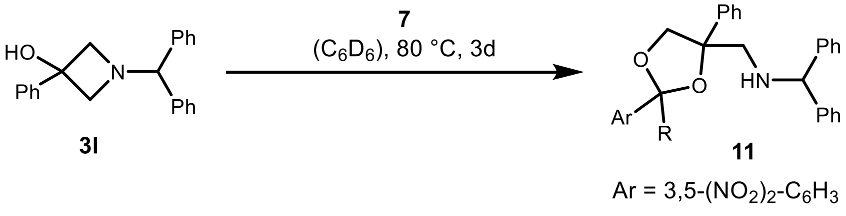

By changing the protecting group to benzhydryl and elevating the temperature to 80 °C, a clean conversion from **3l** to **11** was achieved without the addition of catalysts or additives.

4.4. -Successful dioxolane formation

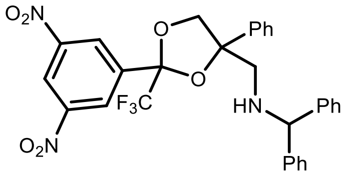

***N*-((2-(3,5-Dinitrophenyl)-4-phenyl-2-(trifluoromethyl)-1,3-dioxolan-4-yl)methyl)-1,1-diphenylmethanamine (11)**

1-Benzhydryl-3-phenylazetidin-3-ol (**3l**, 63.1 mg, 200 µmol, 1.00 equiv) and 1-(3,5-dinitrophenyl)-2,2,2-trifluoroethanone (**7**, 52.8 mg, 200 µmol, 1.00 equiv) were dissolved in dry C_6D_6 (1.00 mL) under inert-gas atmosphere and heated to 80 °C for 72 h. The flask was allowed to cool to rt, and the crude mixture was directly purified via automated FC (CyH/EtOAc 100:0 to 50:50) to obtain the product as a single diastereomer (108.0 mg, 186 µmol, 93%) and a colourless oil.

IR (neat): $\tilde{\nu}$ = 3102 (w), 3028 (w), 2281 (w), 1630 (w), 1599 (w), 1543 (s), 1493 (w), 1453 (w), 1344 (s), 1182 (s), 1135 (s), 1103 (m), 1071 (m), 998 (m), 914 (m), 850 (w), 812 (w), 764 (w), 746 (m), 729 (s), 702 (s), 663 (w), 621 (w), 597 (w), 559 (w), 501 (m), 470 (w). **^{1}H NMR (600 MHz, C_6D_6)**: δ = 8.54 (d, J = 2.1 Hz, 2H), 8.08 (t, J = 2.1 Hz, 1H), 7.24 (td, J = 8.4, 1.4 Hz, 4H), 7.15 – 6.95 (m, 7H), 6.91 – 6.75 (m, 4H), 4.54 (s, 1H), 4.44 (d, J = 8.5 Hz, 1H), 3.95 (d, J = 8.5 Hz, 1H), 2.95 – 2.66 (m, 2H), 1.77 (s, 1H). **^{13}C NMR (151 MHz, C_6D_6)**: δ = 148.0, 144.1, 144.1, 140.4, 138.6, 128.9, 128.5, 128.5, 127.6, 127.6, 127.5, 127.4, 127.0, 125.7, 122.4 (d, J = 288.1 Hz), 120.1, 104.5 (q, J = 33.3 Hz), 89.8, 77.7, 73.7, 67.5, 55.5, 27.2. **^{19}F NMR** (282 MHz, C_6D_6): δ = -81.35. **HRMS (ESI)**: Calculated for **$C_{30}H_{25}F_3N_3O_6$** $[M+H]^+$: 580.1690, found: 580.1682.

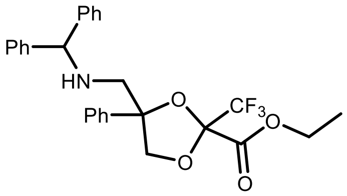

Ethyl 4-((benzhydrylamino)methyl)-4-phenyl-2-(trifluoromethyl)-1,3-dioxolane-2-carboxylate (16)

1-Benzhydryl-3-phenylazetidin-3-ol (**3l**, 63.1 mg, 200 µmol, 1.00 equiv) and ethyl 3,3,3-trifluoro-2-oxopropanoate (**15**, 34.0 mg, 26.5 µL, 200 µmol, 1.00 equiv) were dissolved in dry C_6D_6 (1.00 mL) under inert-gas atmosphere and heated to 80 °C for 72 h. The flask was allowed to cool to rt, and the crude mixture was directly purified via automated FC (CyH/EtOAc 100:0 to 90:10) to obtain the product as a mixture of two diastereomers (dr = 60:40 for isolated substance, 71.0 mg, 146 µmol, 73%) as a colourless oil. The diastereomers were not assigned.

IR (neat): $\tilde{\nu}$ = 1757 (s), 1493 (w), 1451 (w), 1275 (m), 1195 (s), 1138 (s), 1029 (m), 747 (m), 702 (s). **HRMS (ESI)**: Calculated for $C_{27}H_{27}F_3NO_4$ $[M+H]^+$: 486.1887, found: 486.1880.

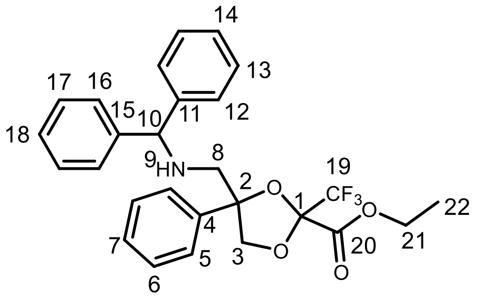

Major-(16):

^{1}H NMR (600 MHz, C_6D_6): δ = 7.35 – 7.29 (m, 1H, H-7), 7.26 – 6.97 (m, 14H), 4.51 (s, 1H, H-10), 4.43 (d, J = 7.7 Hz, 1H, H-3), 4.30 (d, J = 7.8 Hz, 1H, H-3), 3.75 – 3.63 (m, 2H, H-21), 2.82 – 2.77 (m, 2H, H-8), 1.90 (br s, 1H, H-9), 0.62 (t, J = 7.1 Hz, 3H, H-22). **^{13}C NMR (151 MHz, C_6D_6)**: δ = 164.3 (C-20), 144.3 (2C, C-11, C-15), 141.5 (C-4), 128.8 – 127.3 (14C, $C_{arom.}$), 125.7 ($C_{arom.}$), 122.2 (q, J = 287.9 Hz, CF_3-C19), 102.5 (q, J = 33.2 Hz, C-1), 89.7 (C-2), 75.4 (C-3), 67.3 (C-10), 62.4 (C-21), 55.6 (C-8), 13.5 (C-22). **^{19}F NMR (282 MHz, C_6D_6)**: δ = – 79.85.

Minor-(16):

^{1}H NMR (600 MHz, C_6D_6): δ = 7.31 – 7.28 (m, 1H, H-7), 7.26 – 6.97 (m, 14H), 4.58 (s, 1H, H-10), 4.43 (d, J = 7.8 Hz, 1H, H-3), 4.10 (d, J = 7.7 Hz, 1H, H-3), 3.90 – 3.76 (m, 2H, H-21), 2.92 – 2.83 (m, 2H, H-8), 2.31 (br s, 1H, H-9), 0.77 (t, J = 7.1 Hz, 3H, H-22). **^{13}C NMR (151 MHz, C_6D_6)**: δ = 164.7 (C-20), 144.4 (2C, C-11, C-15), 141.1 (C-4), 128.8 – 127.3 (14C, $C_{arom.}$), 125.3 ($C_{arom.}$), 122.3 (q, J = 289.6 Hz, CF_3-C19), 102.8 (q, J = 33.8 Hz, C-1), 90.3 (C-2), 75.5 (C-3), 67.6 (C-10), 63.0 (C-21), 56.0 (C-8), 13.6 (C-22). **^{19}F NMR (282 MHz, C_6D_6)**: δ = – 79.34.

4.5. Determination of the relative configuration of 1,3-dioxolanes

The both diastereotopic CH2-groups of the substrate **11** are unambiguously identified via gHMBC or COSY experiments. The relative configuration of the formed major diastereoisomer could be determined via 2D-^{1}H,^{1}H NOESY experiments. NOE interactions between the phenyl ring and the H_a protons of the 3,5-dinitrobenzene ring could be observed, as well as NOE interactions from both aromatic rings to an identical proton of the dioxolane ring. It can therefore be concluded, that the two aromatic substituents are positioned *syn* to each other. Another set of NOE interactions was observed for the other diastereotopic proton, with respective interactions to the amine and benzhydryl proton.

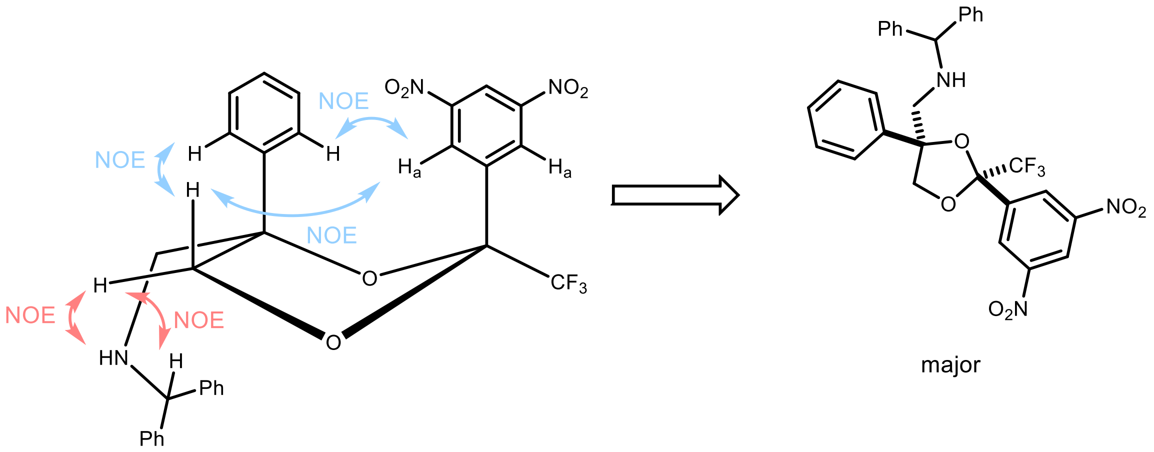

4.6. Conditions for borodioxolane cleavage

Optimisation tables:

Used boronic acids and diols for the deprotection:

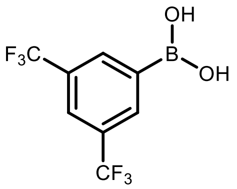

17

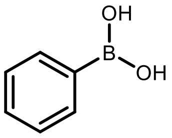

S19

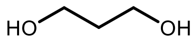

Propan-1,3-diol
S20

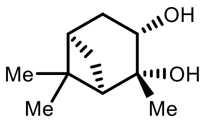

pinanediol
S21

Table S1: Short optimisation of the borodioxolane cleavage.

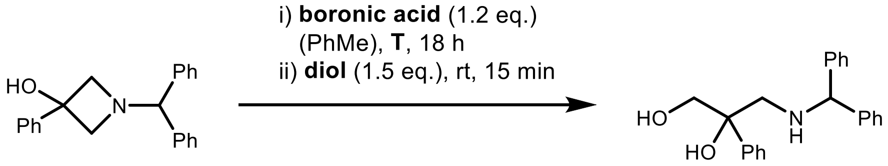

3l **21**

Entry	Boronic acid	Diol	**T** (°C)	Yield **21** NMR[a] (%)
1	**S19**	**S20**	30	-
2	**S19**	**S20**	100	21
3	**17**	**S20**	100	42[b]
4	**17**	**S21**	100	81

[a]Yields were determined by ^{1}H NMR using CH_2Br_2 as an internal standard. [b]42% product, 40% starting material.

Isolation:

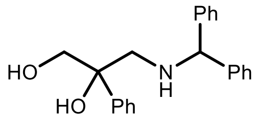

3-(Benzhydrylamino)-2-phenylpropane-1,2-diol (21)

1-Benzhydryl-3-phenylazetidin-3-ol (**3l**, 54.7 mg, 173 µmol, 1.00 equiv) and (3,5-bis(trifluoromethyl)phenyl)boronic acid (61.9 mg, 240 µmol, 1.38 equiv) were dissolved in toluene (1.50 mL) and the mixture was heated to 100 °C for 18 h. The mixture was allowed to cool to rt, diluted with EtOAc (3 mL) and (1*S*,2*S*,3*R*,5*S*)-2,3-pinanediol (50.0 mg, 294 µmol, 1.69 equiv) was added. The mixture was stirred at rt for 15 min and the organic layer was washed with brine, dried over Na_2SO_4 and the solvent evaporated under reduced pressure. The crude product was purified via automated FC (CH_2Cl_2/MeOH 100:0 to 90:10), then RP-MPLC (H_2O/MeCN 50:50) to obtain the product (28.1 mg, 84.3 µmol, 49%) as a colourless oil.

IR (neat): $\tilde{\nu}$ = 3368 (br), 3061 (w), 3027 (w), 2921 (w), 2863 (w), 1493 (m), 1451 (m), 1388 (w), 1054 (m), 899 (w), 762 (m), 746 (m), 700 (s), 550 (w), 439 (w). **^{1}H NMR (400 MHz, $CDCl_3$)**: δ = 7.46 – 7.38 (m, 2H), 7.37 – 7.18 (m, 14H), 4.77 (s, 1H), 3.80 (d, *J* = 11.5 Hz, 1H), 3.62 (dd, *J* = 11.5, 0.8 Hz, 1H), 3.10 (d, *J* = 12.0 Hz, 1H), 3.02 (dd, *J* = 12.1, 0.9 Hz, 1H), 2.89 (s, 2H). **^{13}C NMR (101 MHz, $CDCl_3$)**: δ = 143.2, 143.0, 142.8, 128.8, 128.8, 128.6, 127.6, 127.5, 127.4, 127.2, 125.2, 75.5, 70.1, 67.3, 55.3.[d] **HRMS (ESI):** Calculated for $C_{22}H_{24}NO_2$ $[M+H]^+$: 334.1802, Found: 334.1794.

[d] Signals missing due to signal overlapping.

5. References

1. Rosenau, C. P.; Jelier, B. J.; Gossert, A. D.; Togni, A. *Angew. Chem. Int. Ed.* **2018,** *57*, 9528–9533.
2. Ishida, N.; Shimamoto, Y.; Murakami, M. *Angew. Chem. Int. Ed.* **2012,** *51*, 11750–11752.
3. Saxena, A.; Ghosh, N. *J. Org. Chem.* **2023,** *88*, 300–309.
4. Bodireddy, M. R.; Mahla, R. S.; Khaja Mohinuddin, P. M.; Reddy, G. T.; Raghava Prasad, D. V.; Kumar, H.; Reddy, N. C. G. *RSC Adv.* **2016,** *6*, 75651–75663.
5. Chai, Y.-M.; Zou, Q.; Guo, Z.-X.; Qin, Y.-J.; Zhang, P. *Tetrahedron Lett.* **2023,** *122*, 154517.
6. Chen, W.; Xu, H.; Wu, R.; Chen, Y.; Yu, P.; Jin, Y. *Org. Biomol. Chem.* **2023,** *21*, 5547–5552.
7. Heffernan, S. J.; Beddoes, J. M.; Mahon, M. F.; Hennessy, A. J.; Carbery, D. R. *Chem. Commun.* **2013,** *49*, 2314–2316.
8. Bai, F.; Wang, N.; Bai, Y.; Ma, X.; Gu, C.; Dai, B.; Chen, J. *J. Org. Chem.* **2023,** *88*, 2985–2998.
9. Fuhrmann, J.; Haber, H.; Haupt, M.; Henning, H.-G. *J. Prakt. Chem.* **1982,** *324*, 1055–1059.
10. Lachhein, S.; Dehmer, K. Process for the preparation of N-alkyl sulfonamides. EP19920104778 19920319, Mar 19, 1992.
11. Johnson, D. C.; Widlanski, T. S. *J. Org. Chem.* **2003,** *68*, 5300–5309.
12. Andna, L.; Miesch, L. *Org. Biomol. Chem.* **2019,** *17*, 5688–5692.
13. Laha, J. K.; Gupta, P.; Hazra, A. *Beilstein J. Org. Chem.* **2023,** *19*, 771–777.
14. Fuhrmann, J.; Haupt, M.; Henning, H.-G. *J. Prakt. Chem.* **1984,** *326*, 177–186.
15. Li, W.; Duan, Y.; Zhang, M.; Cheng, J.; Zhu, C. *Chem. Commun.* **2016,** *52*, 7596–7599.
16. Shennan, B. D. A.; Sánchez-Alonso, S.; Rossini, G.; Dixon, D. J. *J. Am. Chem. Soc.* **2023,** *145*, 21745–21751.
17. Venkov, A. P.; Vodenicharov, D. M. *Synthesis* **1990,** *1990*, 253–255.
18. Sandvoß, A.; Maag, H.; Daniliuc, C. G.; Schollmeyer, D.; Wahl, J. M. *Chem. Sci.* **2022,** *13*, 6297–6302.
19. Miller, D. D.; Fowble, J.; Patil, P. N. *J. Med. Chem.* **1973,** *16*, 177–178.
20. Ishida, N.; Shimamoto, Y.; Yano, T.; Murakami, M. *J. Am. Chem. Soc.* **2013,** *135*, 19103–19106.
21. Chatterjee, S. S.; Shoeb, A. *Synthesis* **1973,** *1973*, 153–154.

6. UV Spectra

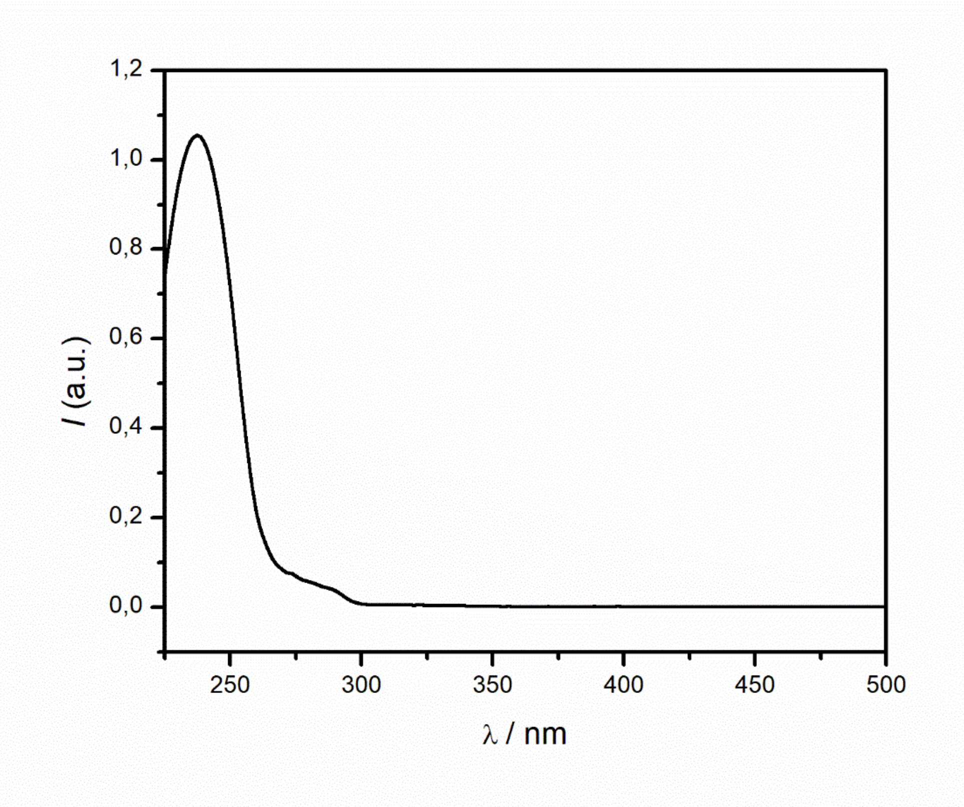

Figure S1: UV Spectrum of **(1a)** in acetonitrile at ca. 7×10^{-5} mol/L.

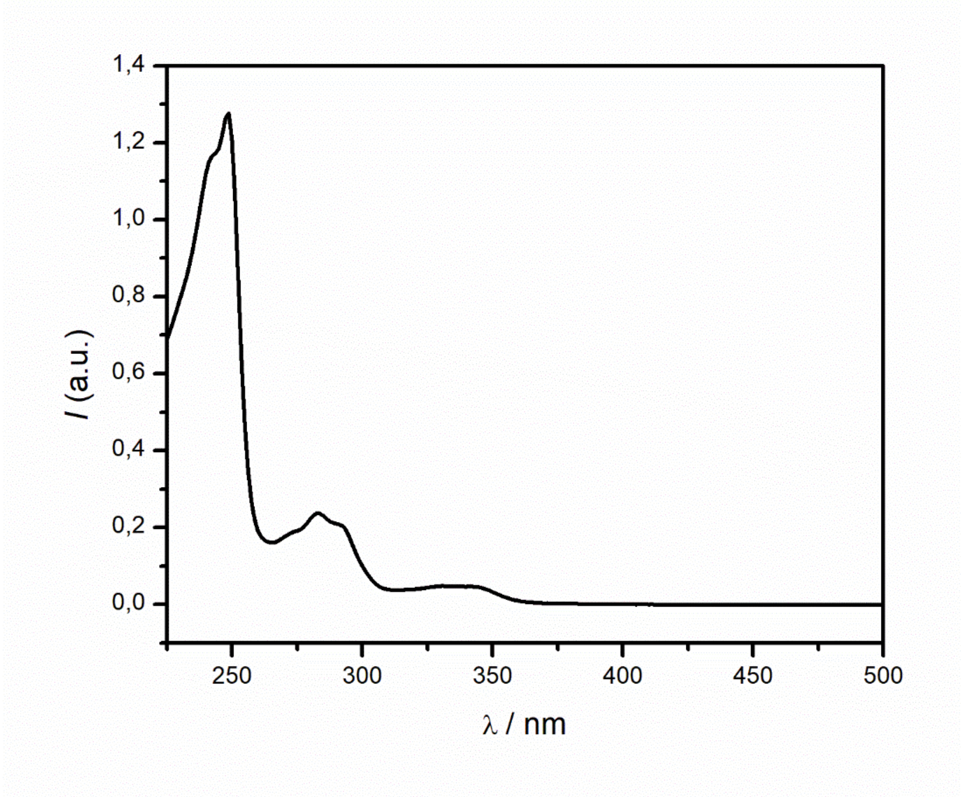

Figure S2: UV Spectrum of **(1b)** in acetonitrile at ca. 2×10^{-5} mol/L.

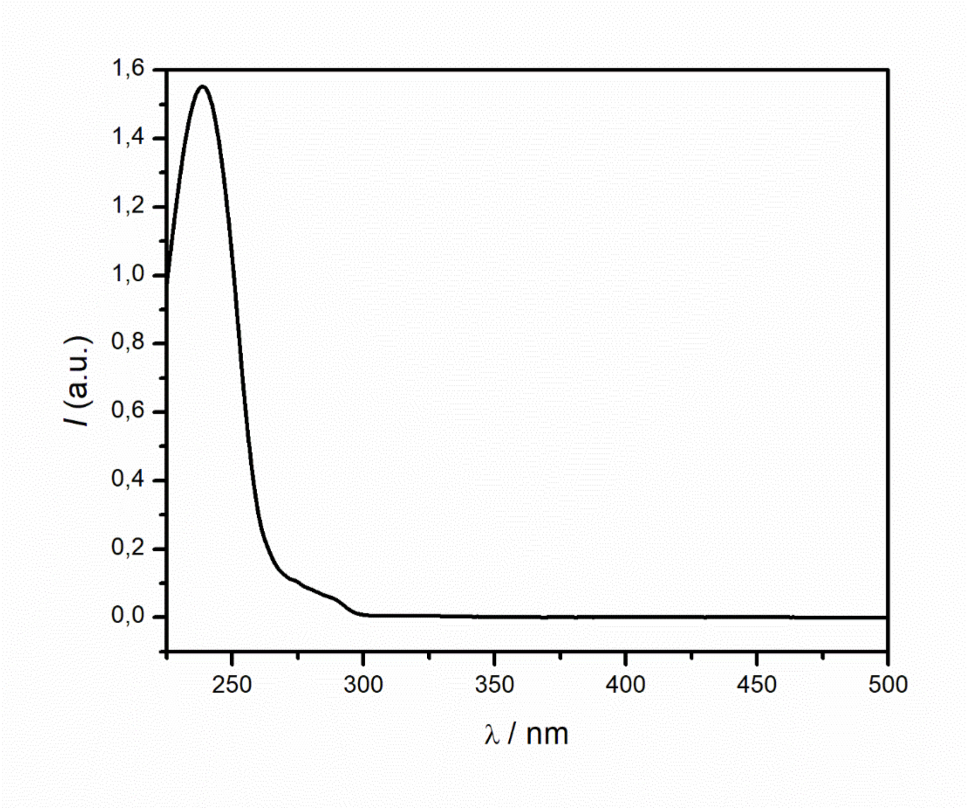

Figure S3: UV Spectrum of **(1c)** in acetonitrile at ca. 7 × 10^{-5} mol/L.

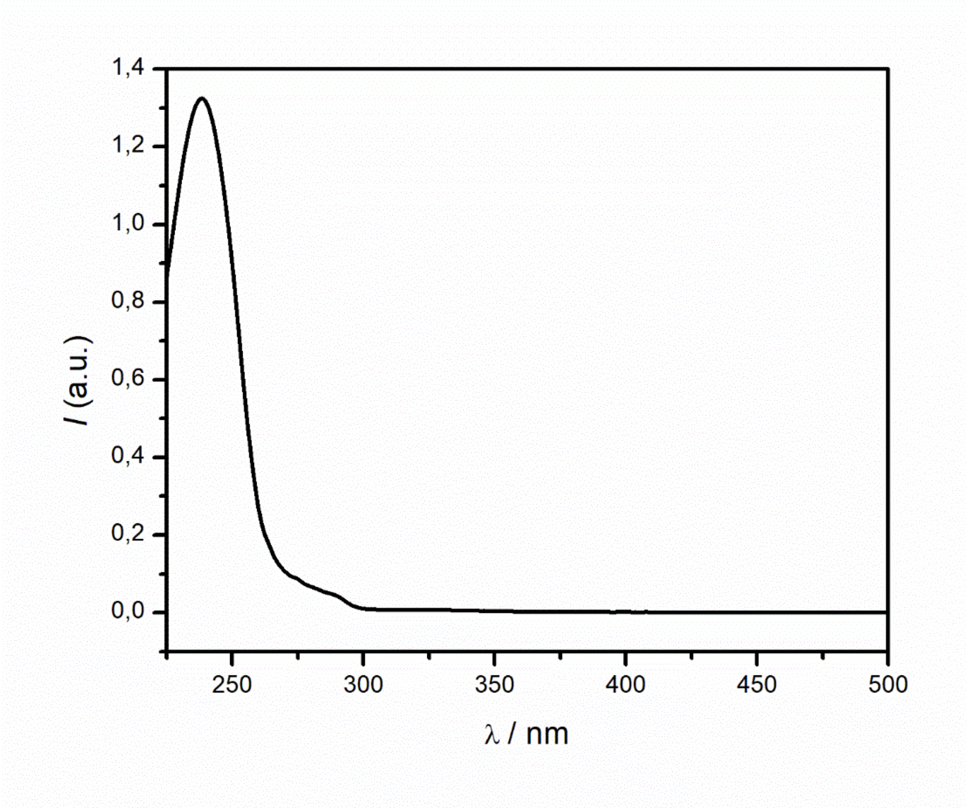

Figure S4: UV Spectrum of **(1d)** in acetonitrile at ca. 6 × 10^{-5} mol/L.

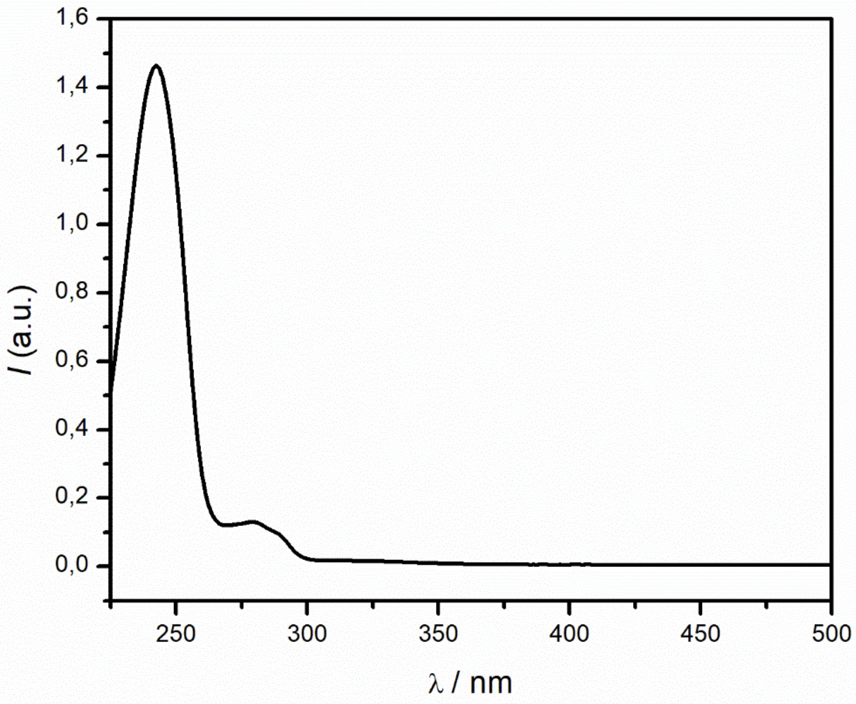

Figure S5: UV Spectrum of **(1e)** in acetonitrile at ca. 1 × 10^{-4} mol/L.

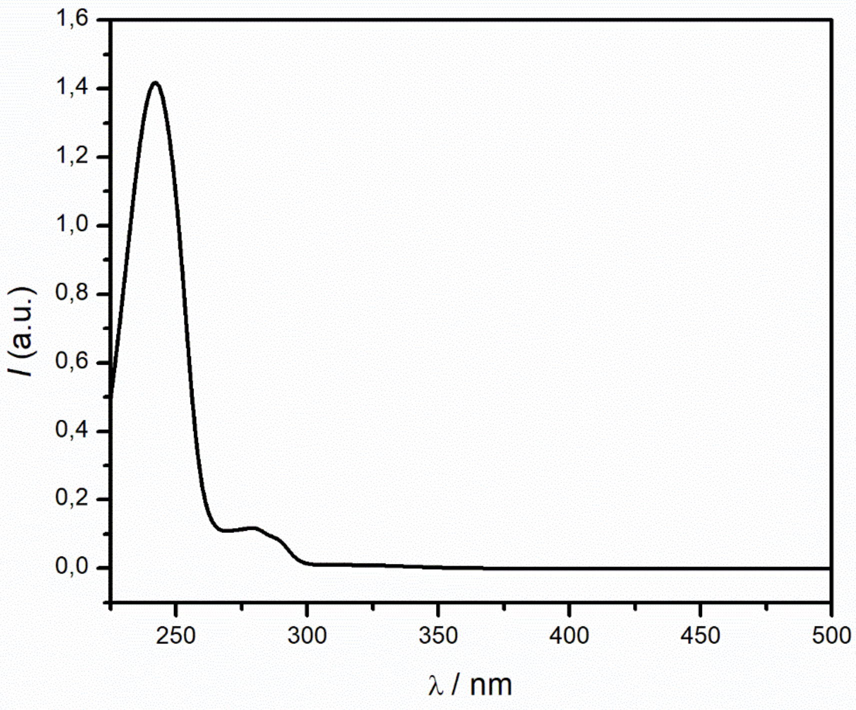

Figure S6: UV Spectrum of **(1f)** in acetonitrile at ca. 1 × 10^{-4} mol/L.

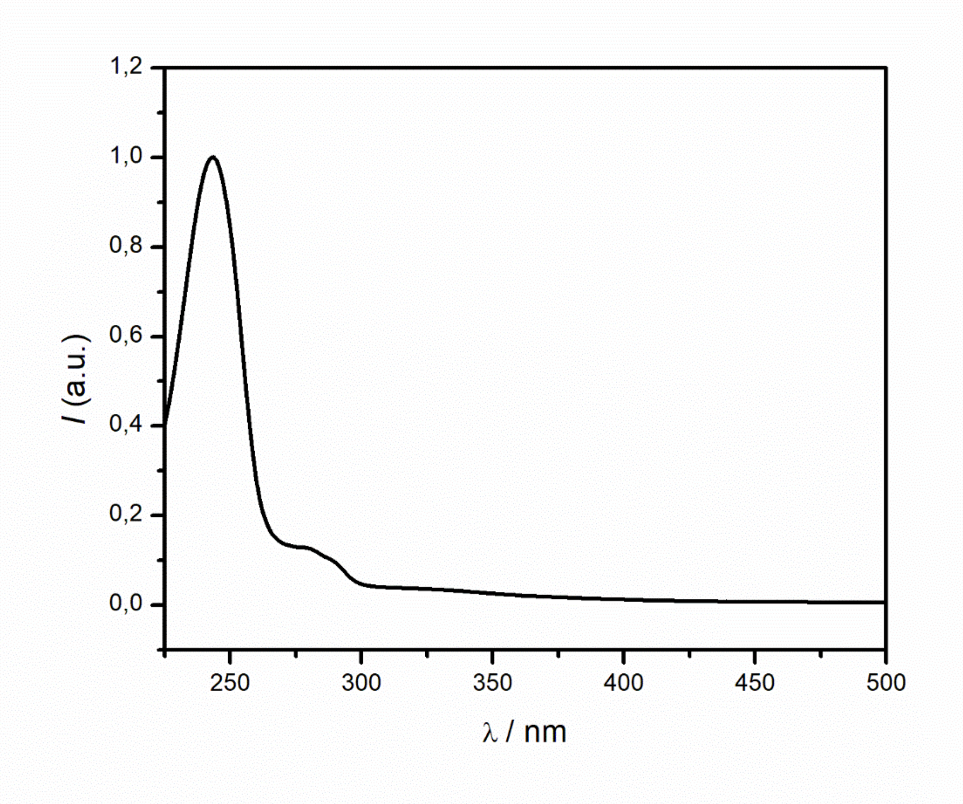

Figure S7: UV Spectrum of **(1g)** in acetonitrile at ca. 6 × 10^{-5} mol/L.

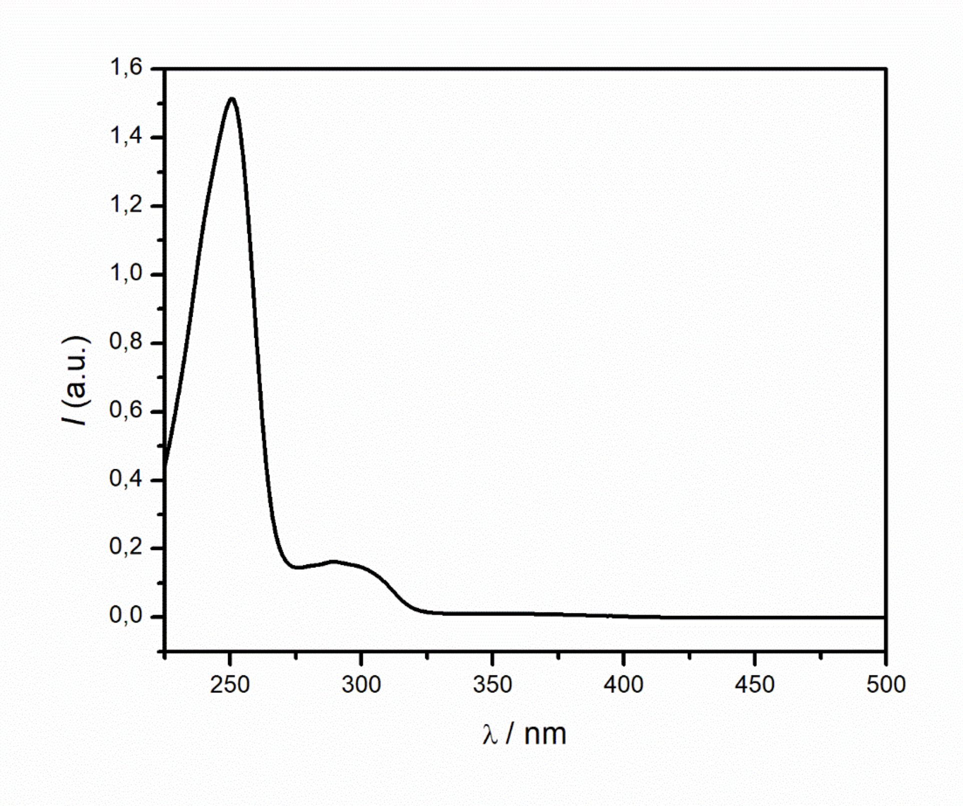

Figure S8: UV Spectrum of **(1h)** in acetonitrile at ca. 5 × 10^{-5} mol/L.

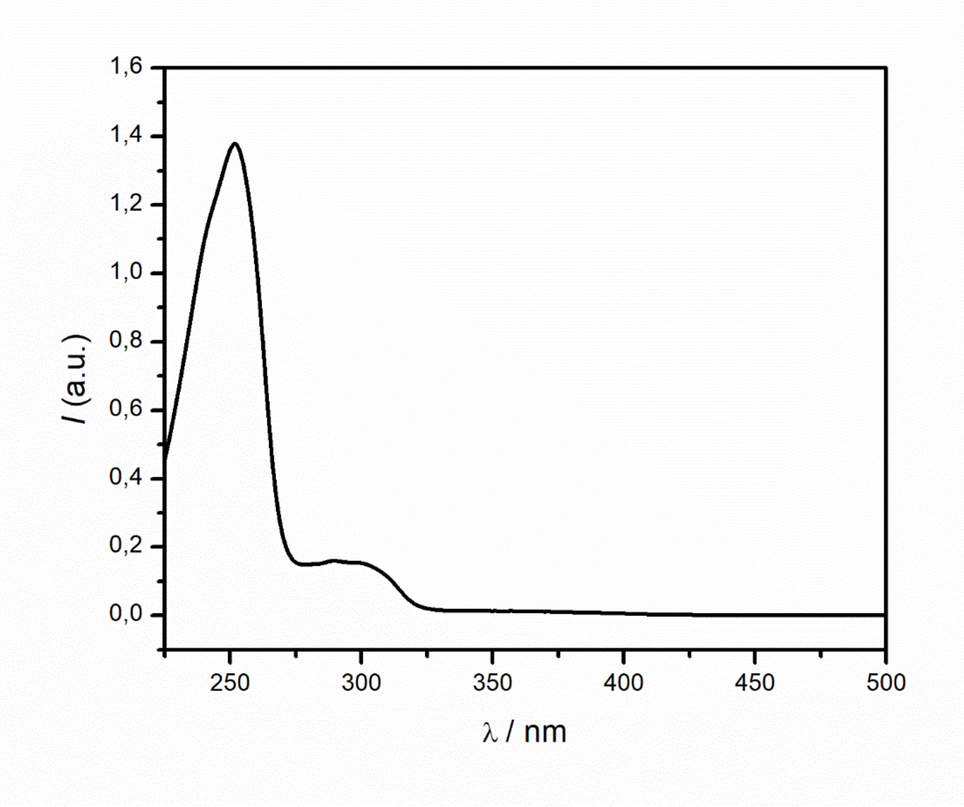

Figure S9: UV Spectrum of **(1i)** in acetonitrile at ca. 4 × 10^{-5} mol/L.

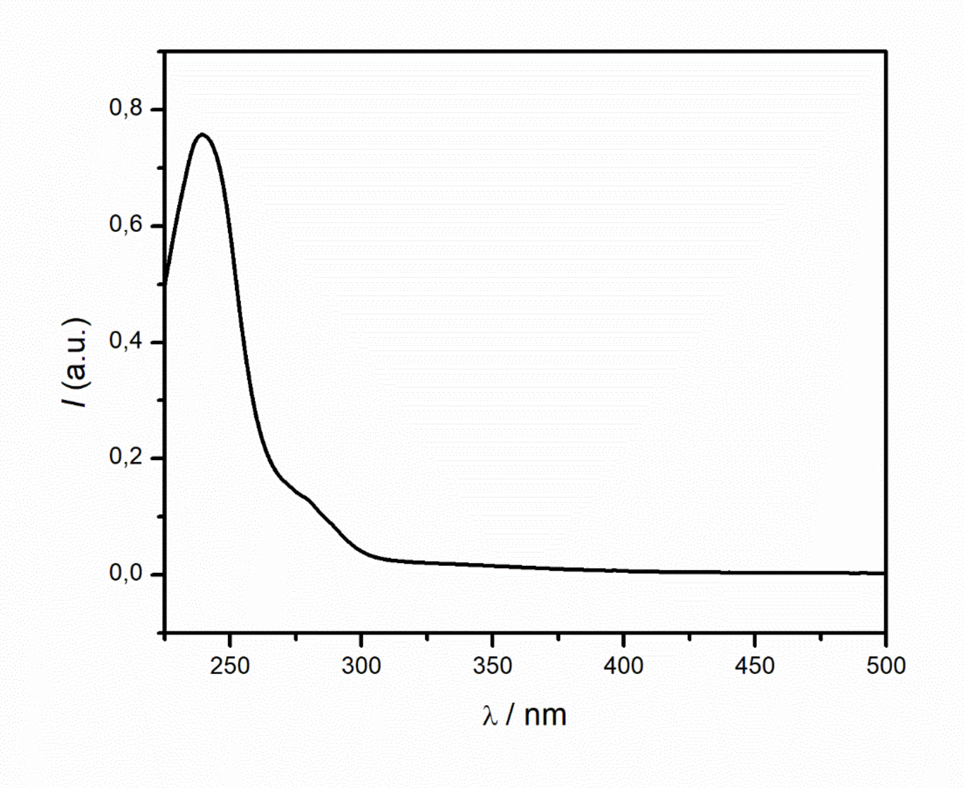

Figure S10: UV Spectrum of **(1j)** in acetonitrile at ca. 7 × 10^{-5} mol/L.

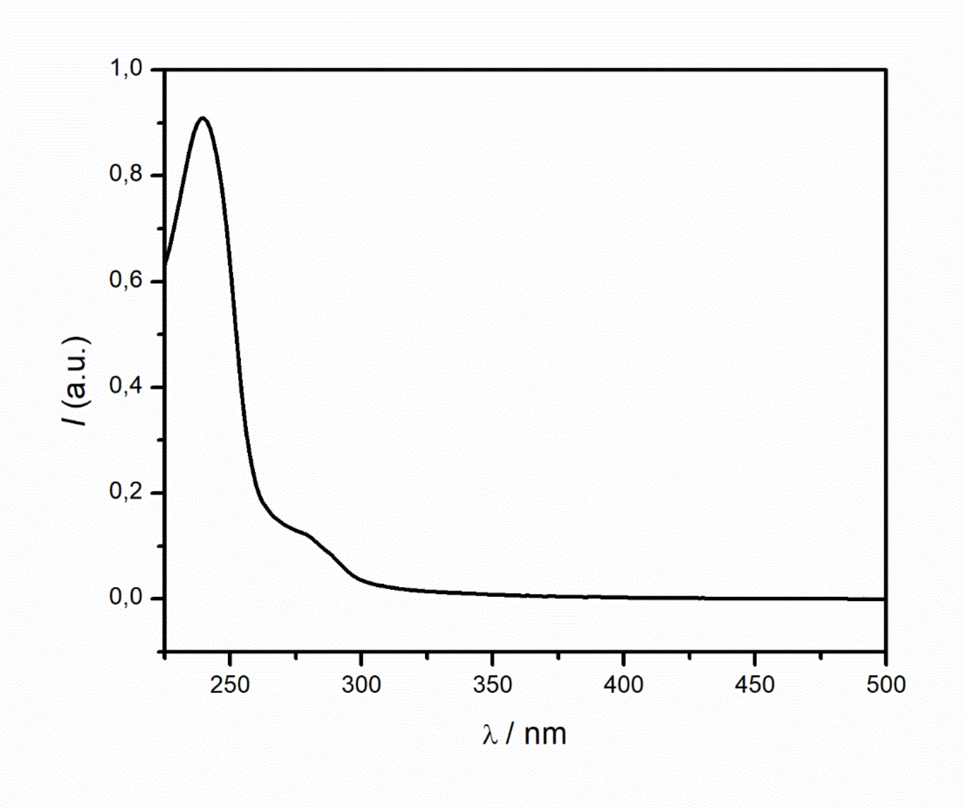

Figure S11: UV Spectrum of **(1k)** in acetonitrile at ca. 8 × 10^{-5} mol/L.

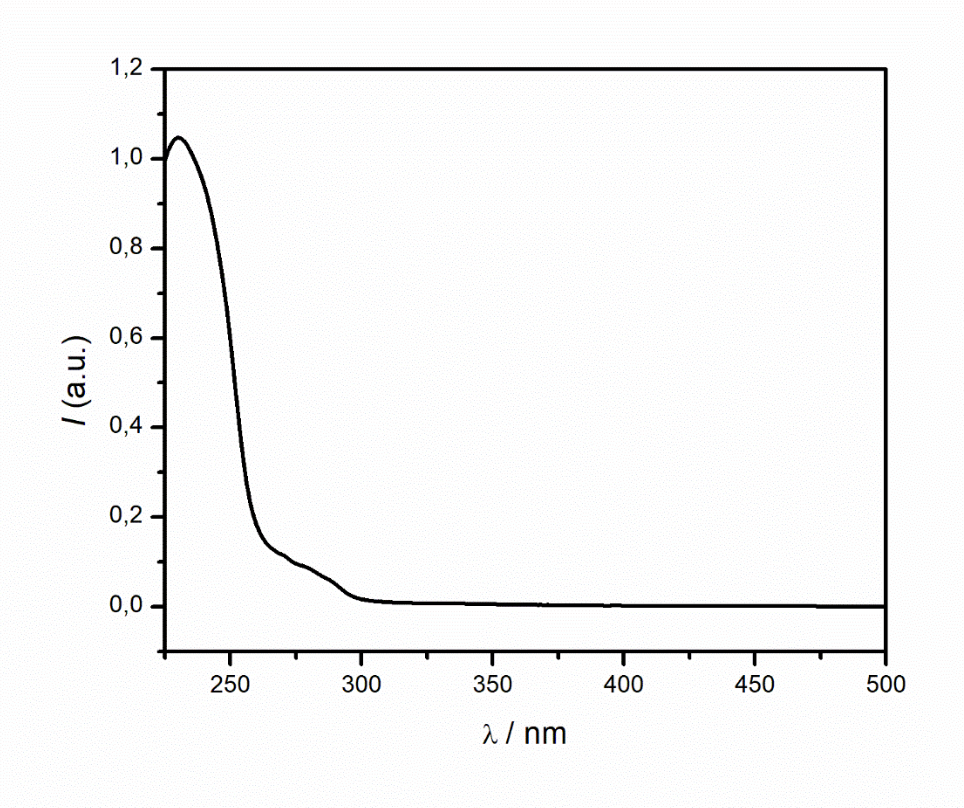

Figure S12: UV Spectrum of **(1l)** in acetonitrile at ca. 7 × 10^{-5} mol/L.

7. NMR Spectra

(S1), ^{1}H, $CDCl_3$, 400 MHz

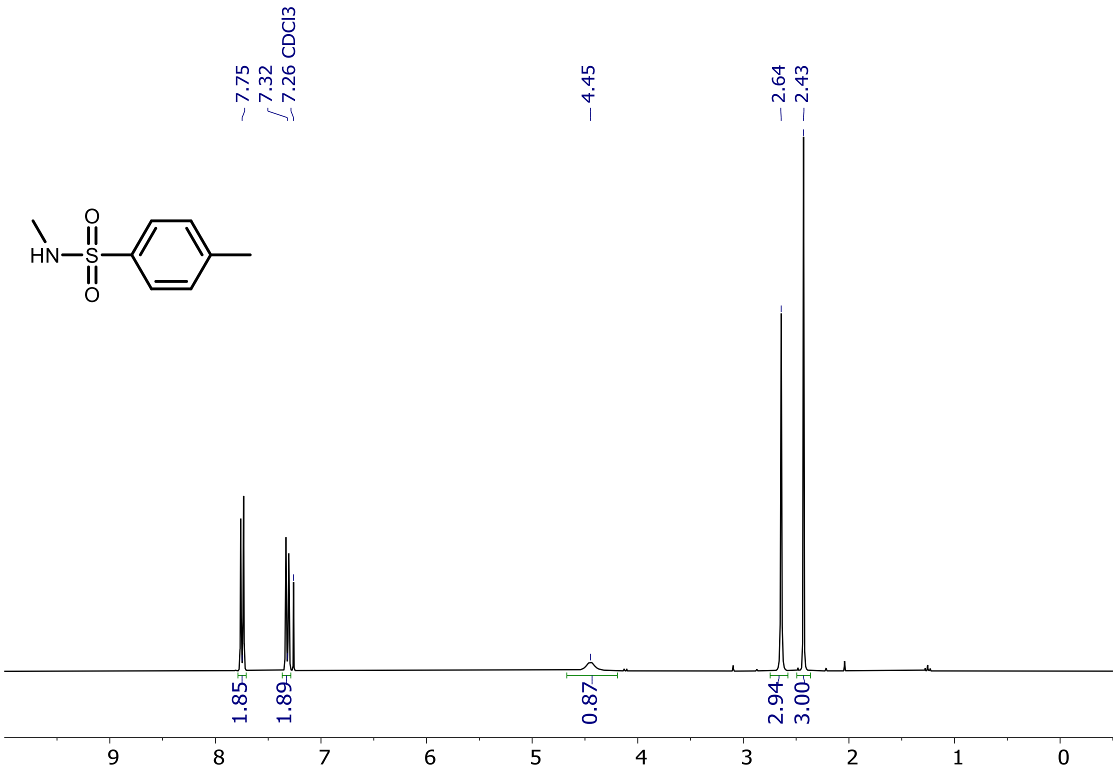

(S1), ^{13}C, $CDCl_3$, 101 MHz

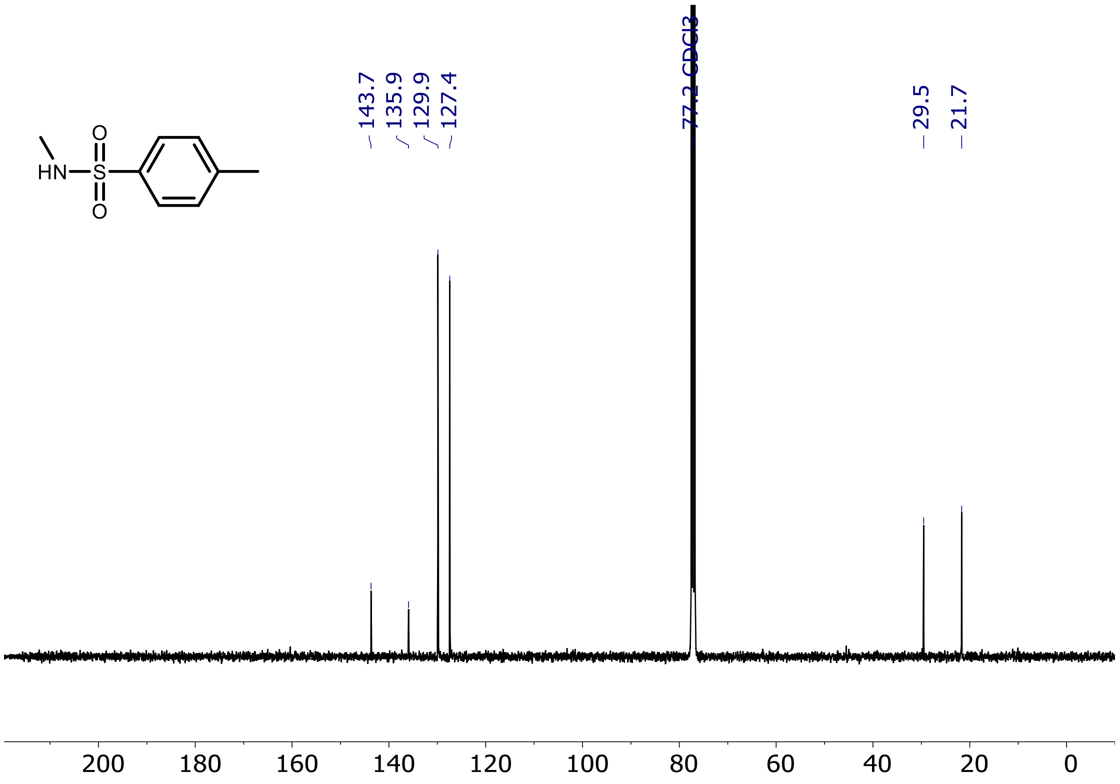

(1a), ^{1}H, $CDCl_3$, 400 MHz

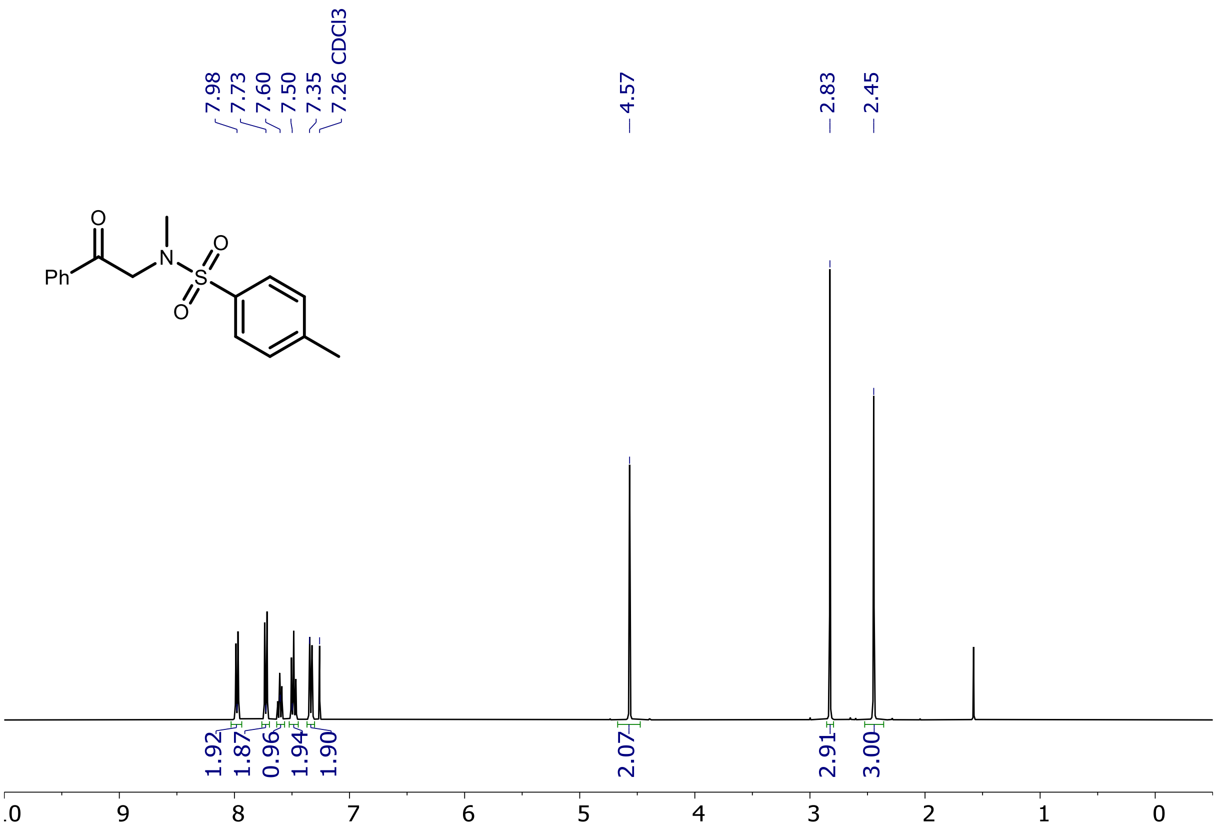

(1a), ^{13}C, $CDCl_3$, 101 MHz

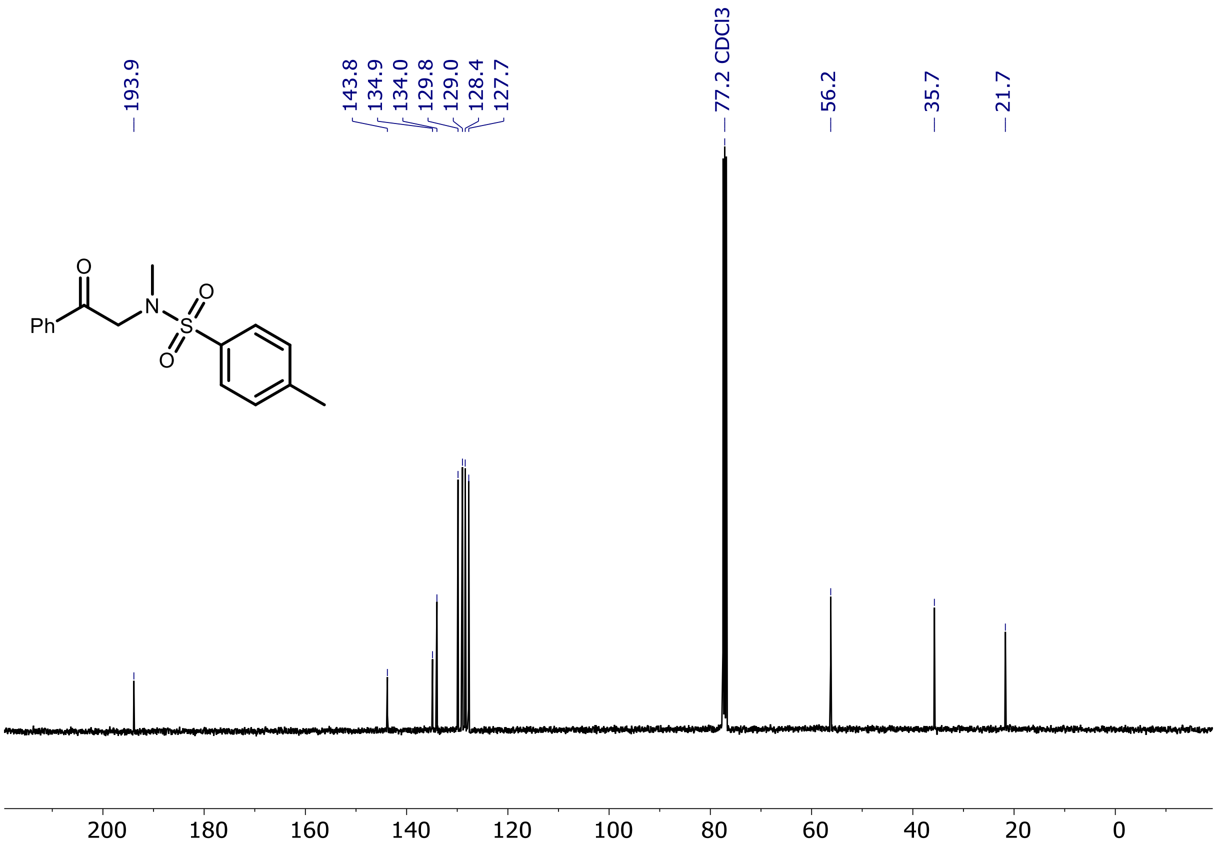

(S2), ^{1}H, $CDCl_3$, 600 MHz

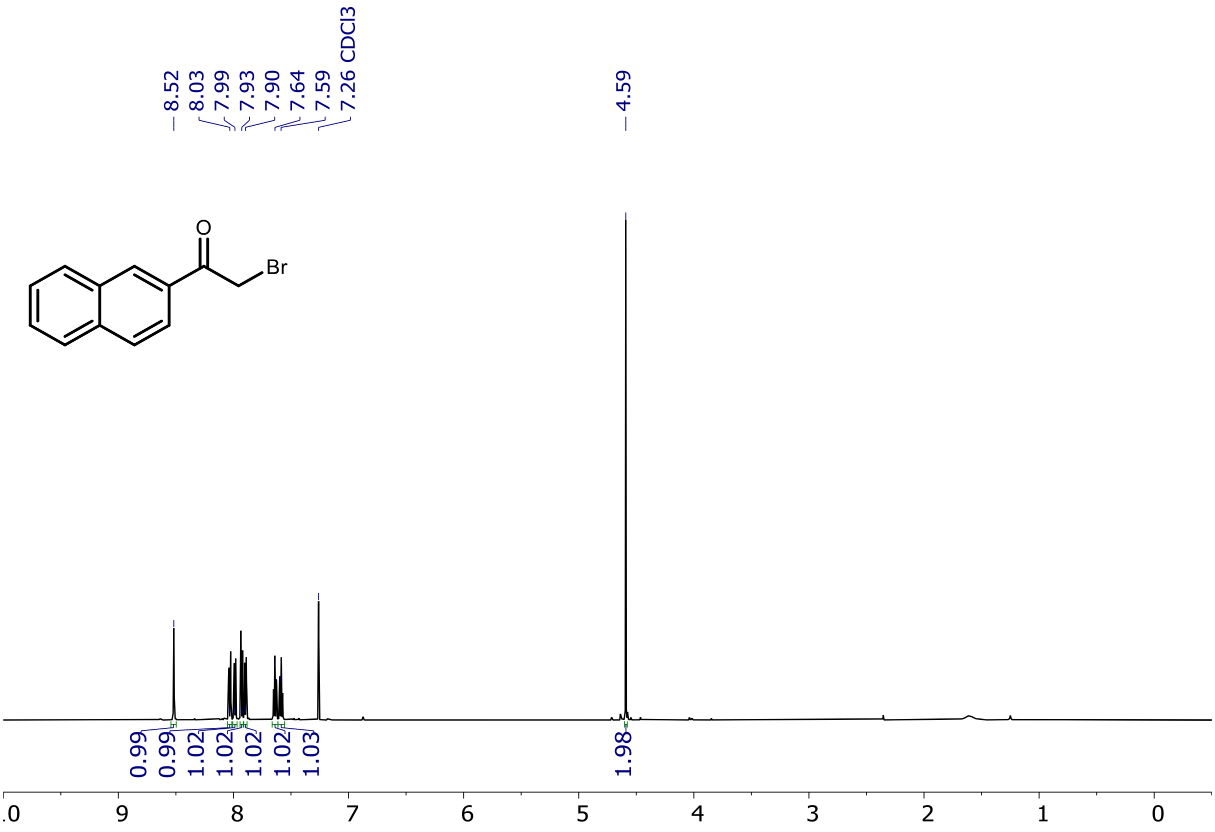

(S2), ^{13}C, $CDCl_3$, 151 MHz

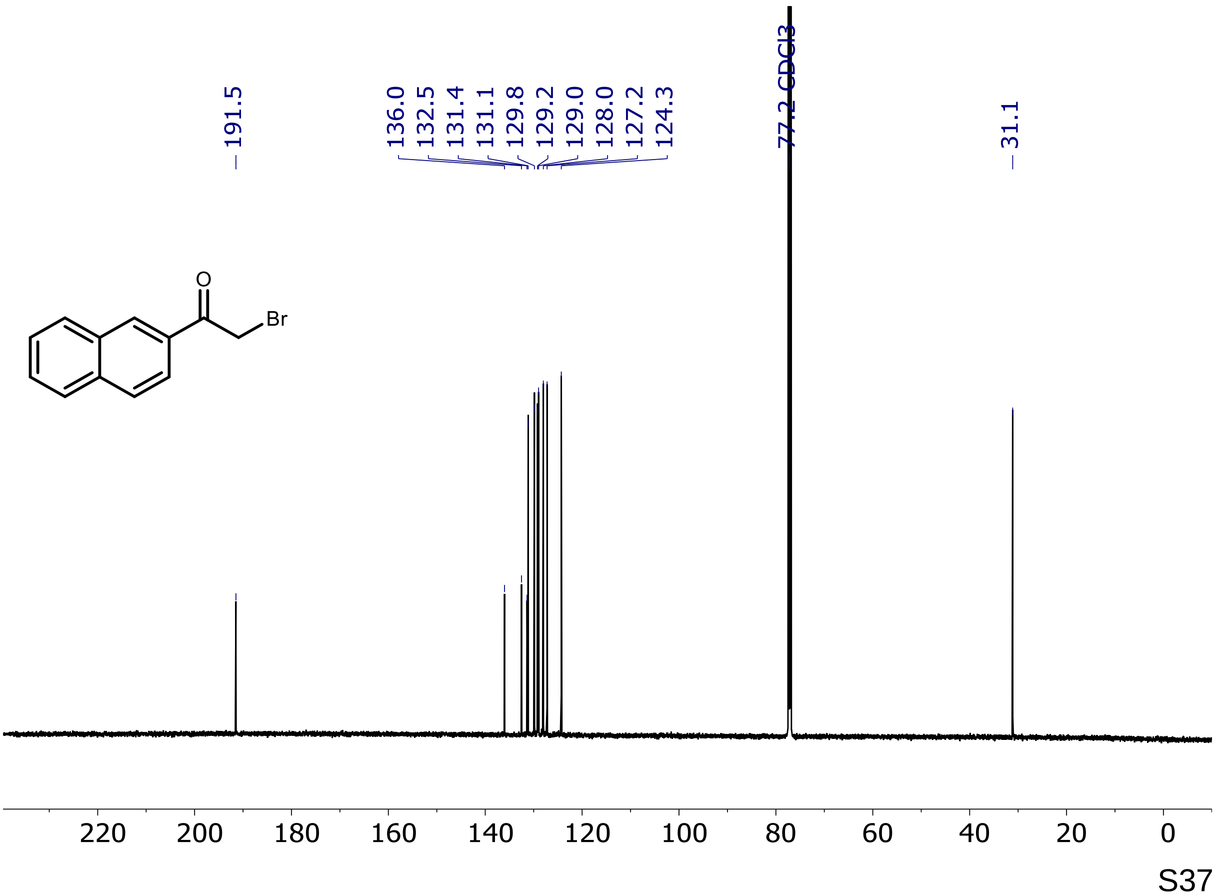

(1b), ^{1}H, $CDCl_3$, 400 MHz

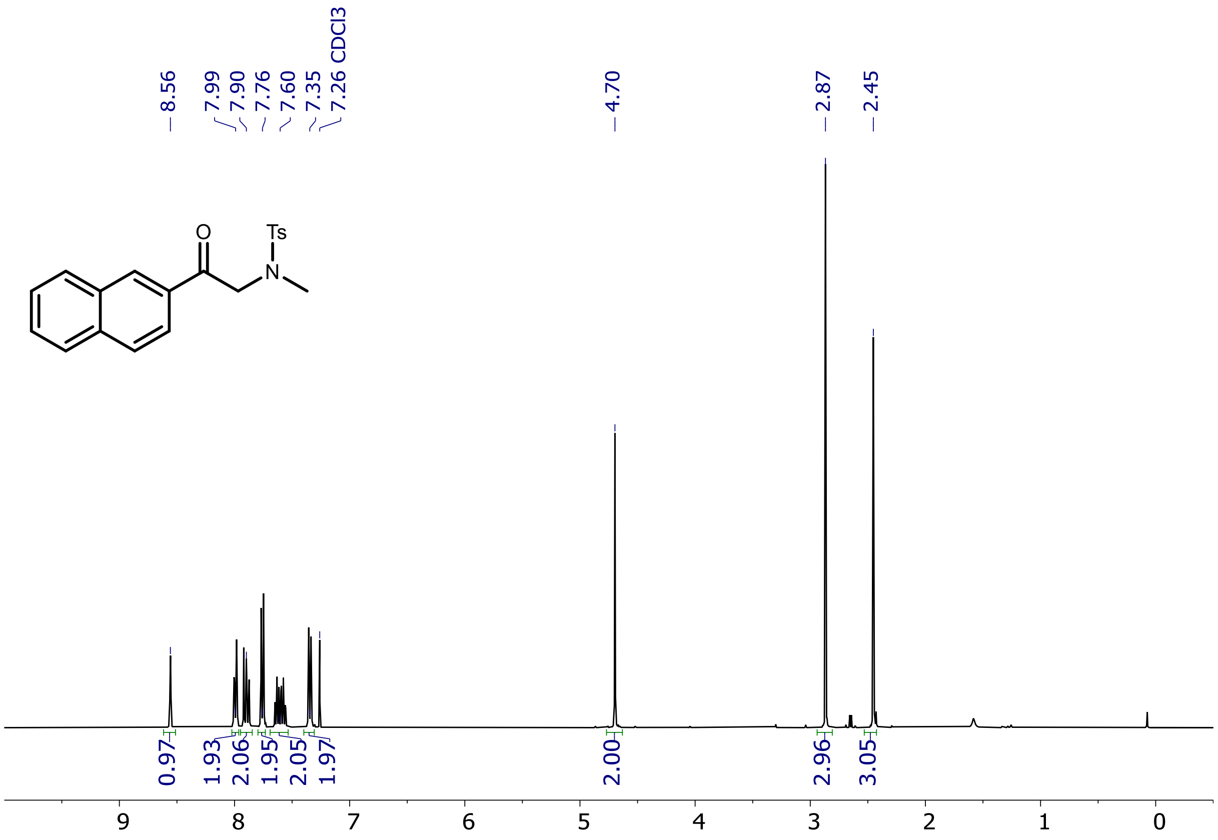

(1b), ^{13}C, $CDCl_3$, 101 MHz

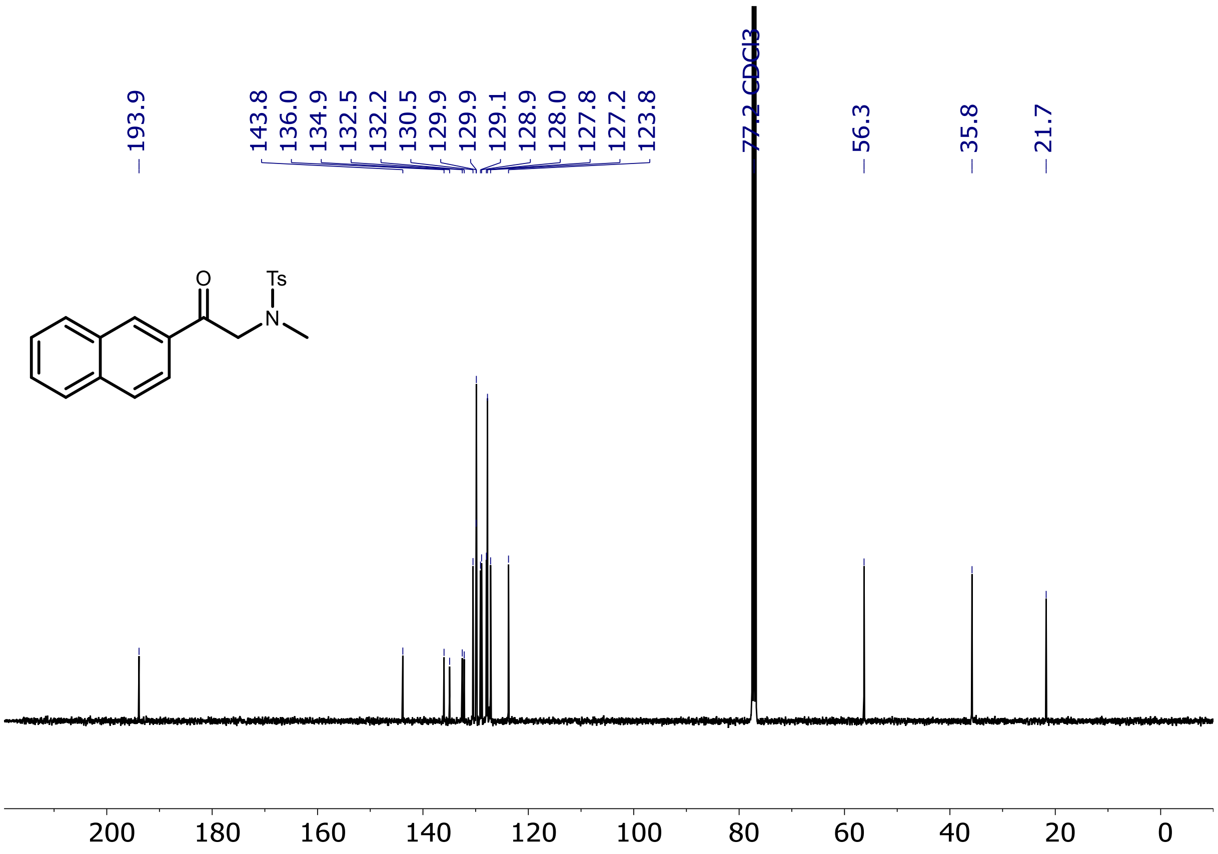

(S3), ^{1}H, $CDCl_3$, 400 MHz

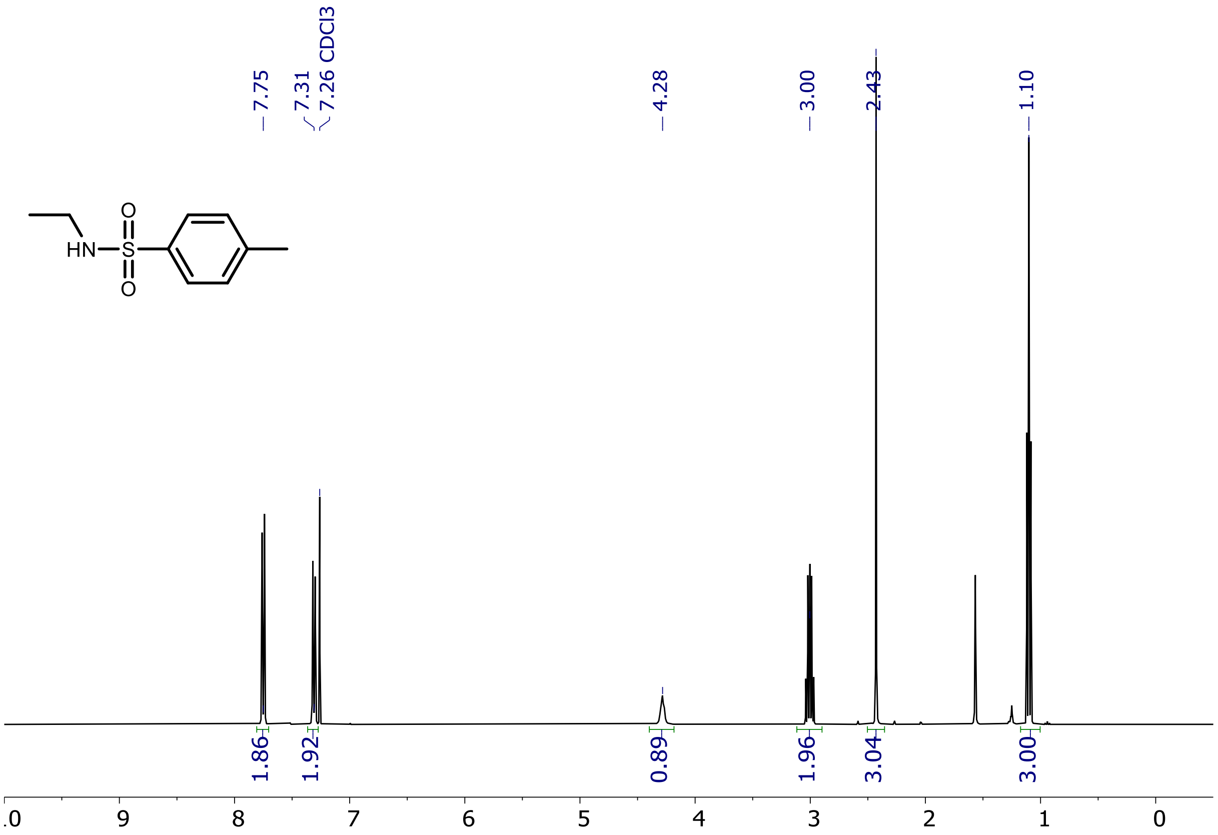

(S3), ^{13}C, $CDCl_3$, 101 MHz

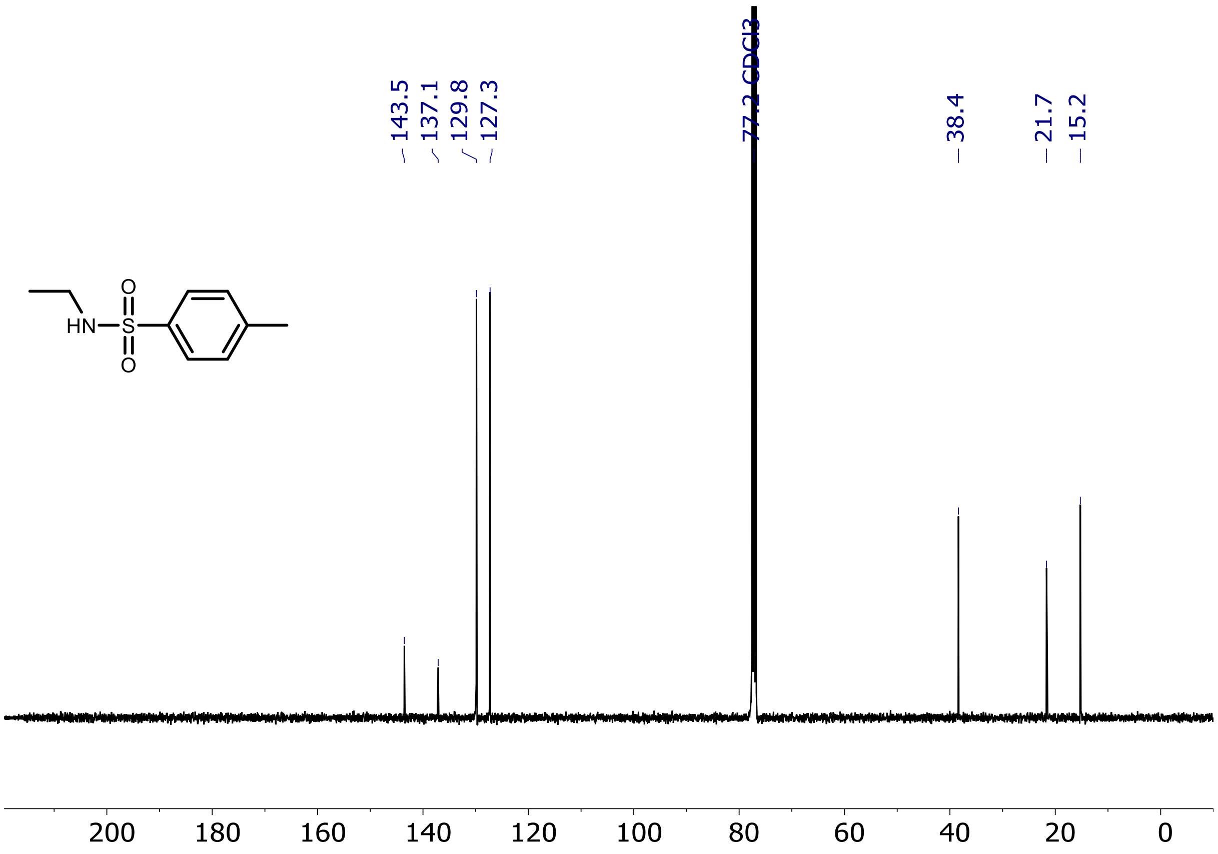

(1c), ^{1}H, $CDCl_3$, 400 MHz

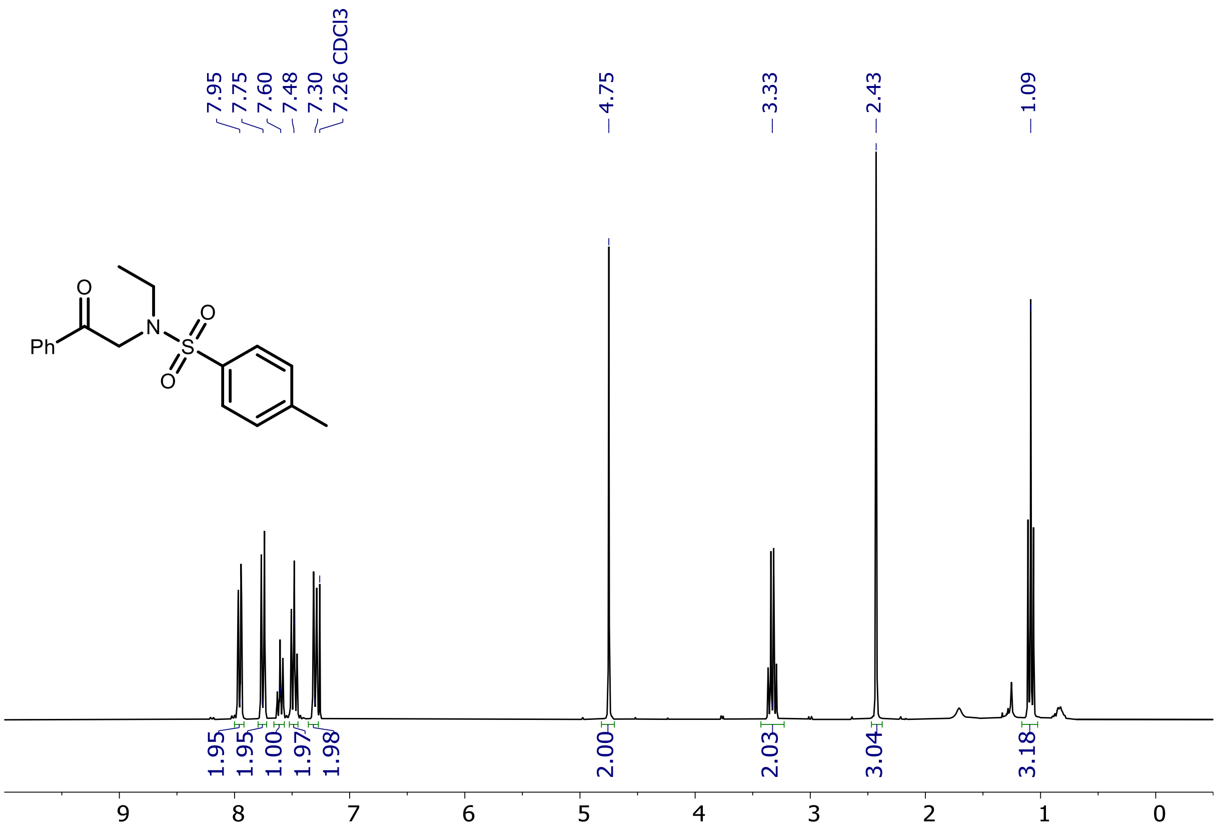

(1c), ^{13}C, $CDCl_3$, 101 MHz

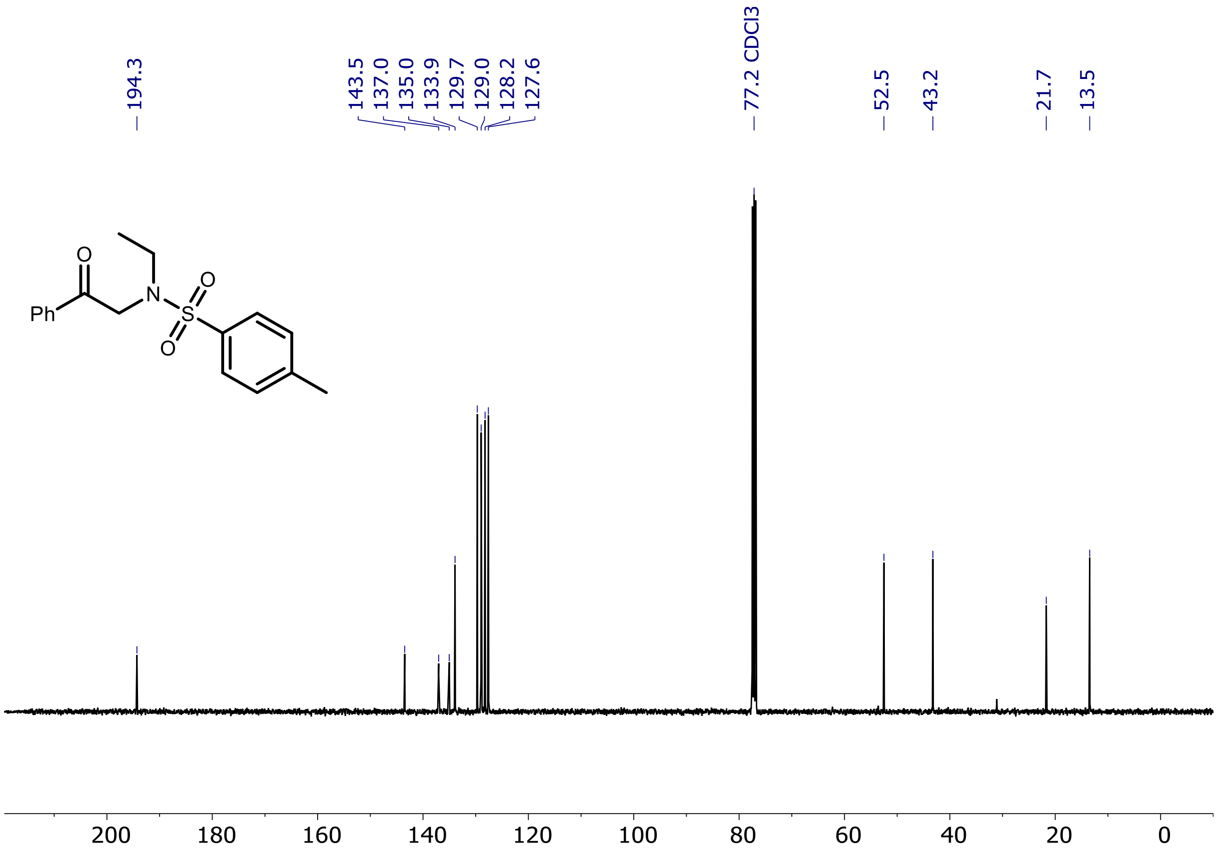

(S4), ^{1}H, $CDCl_3$, 400 MHz

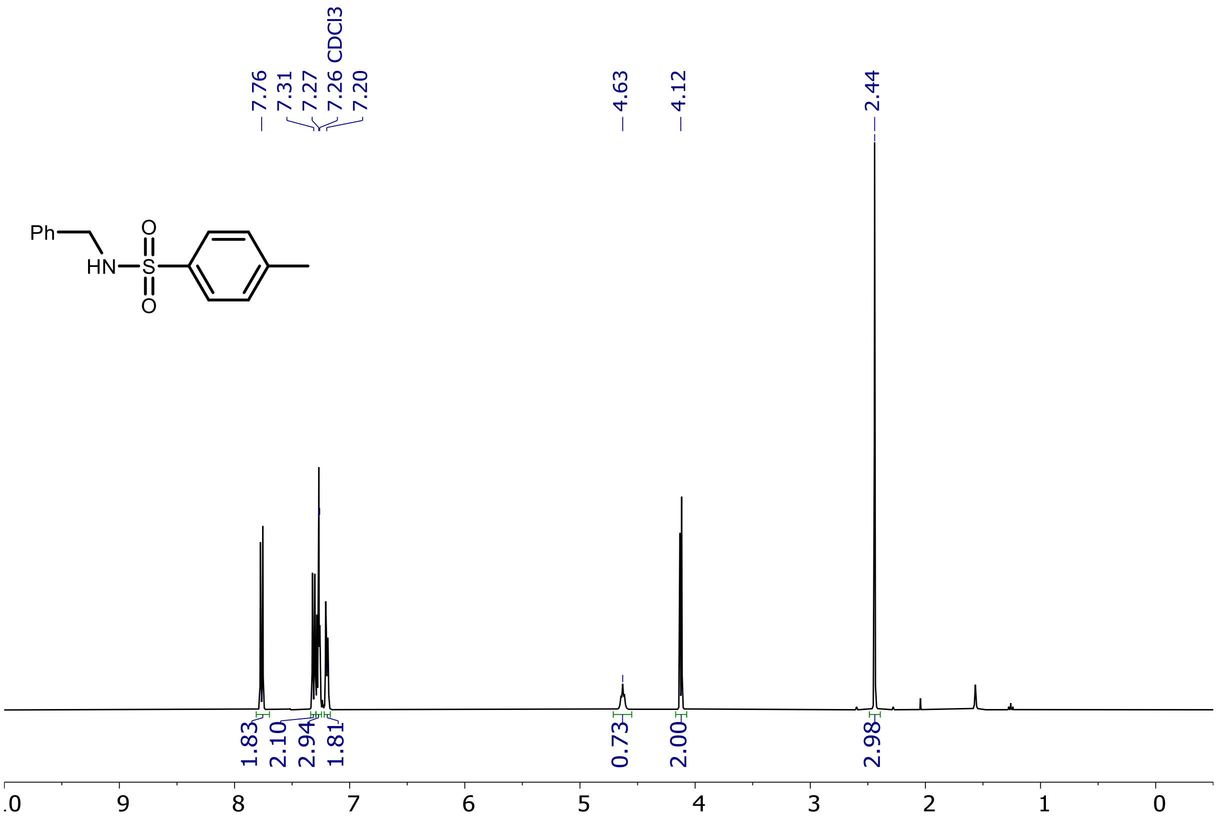

(S4), ^{13}C, $CDCl_3$, 101 MHz

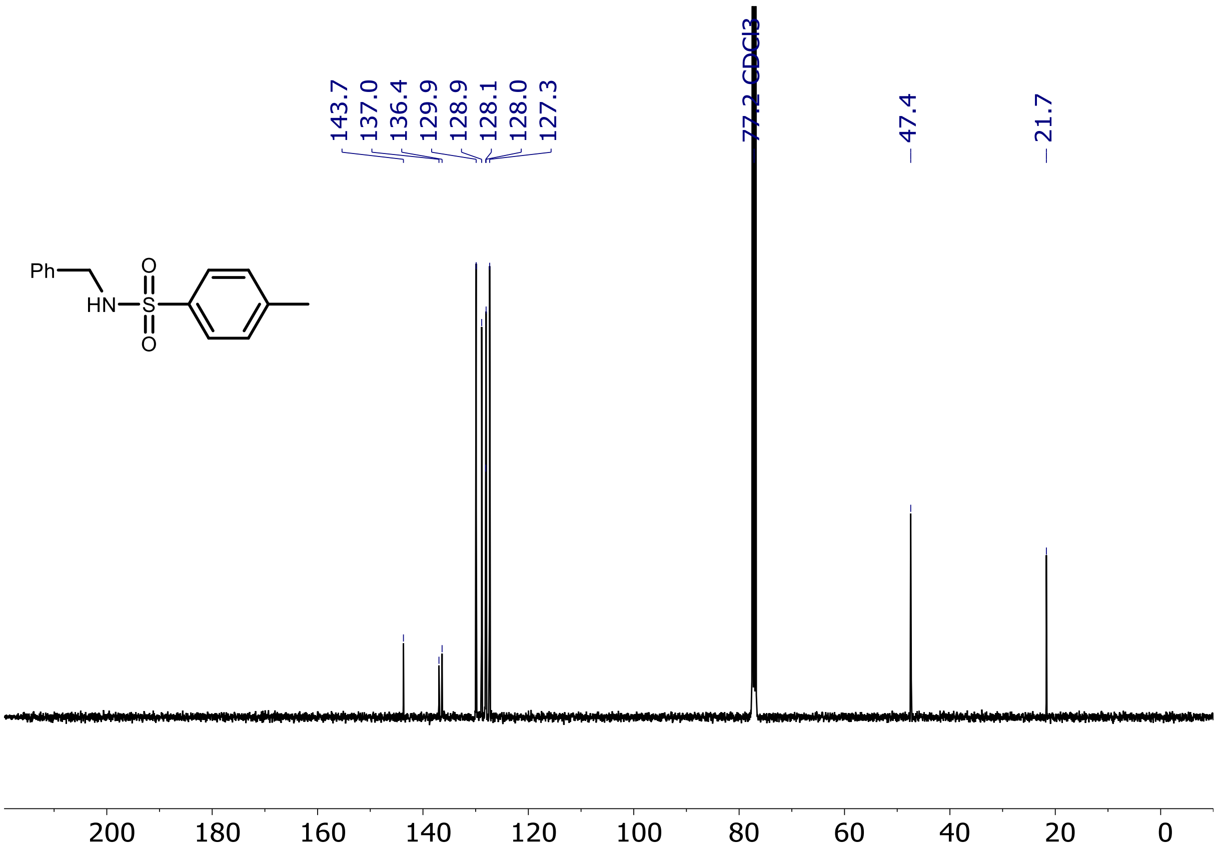

(1d), ^{1}H, DMSO-d_6, 400 MHz

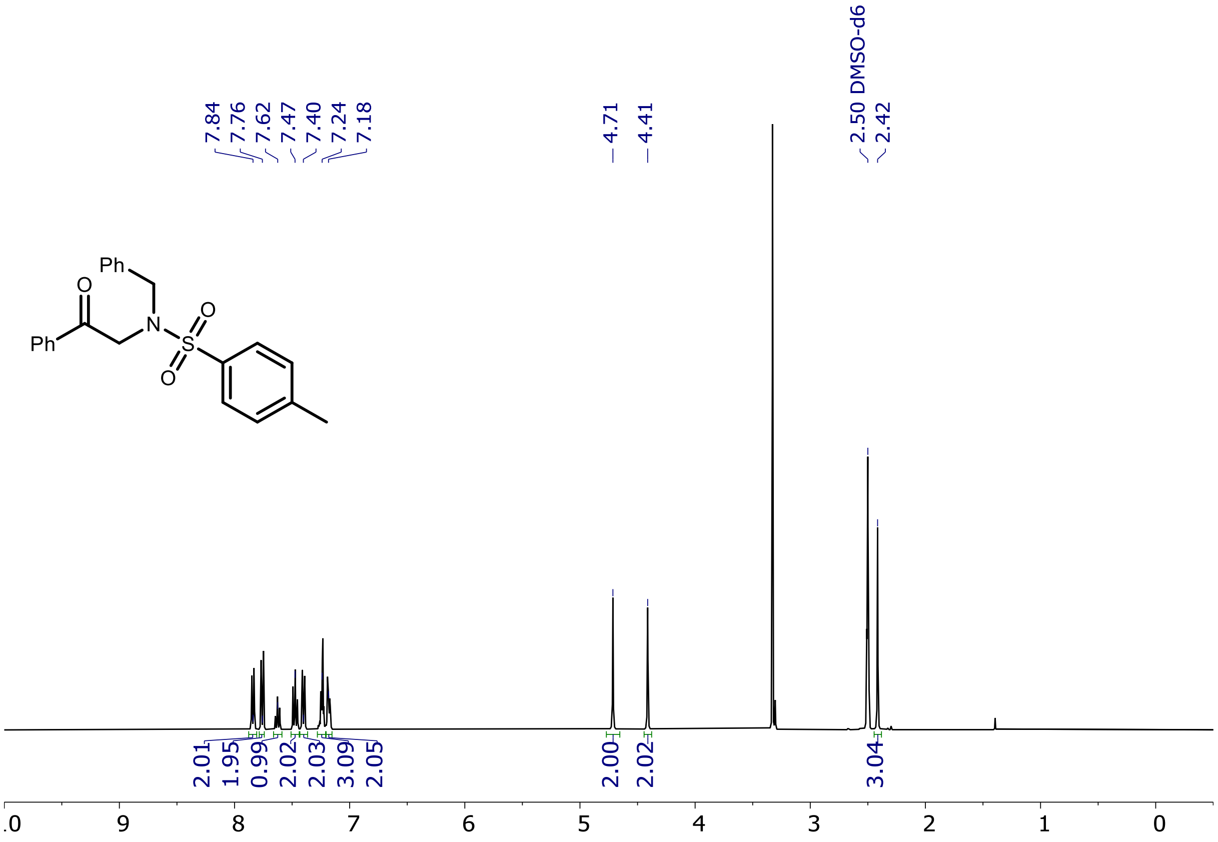

(1d), ^{13}C, DMSO-d_6, 101 MHz

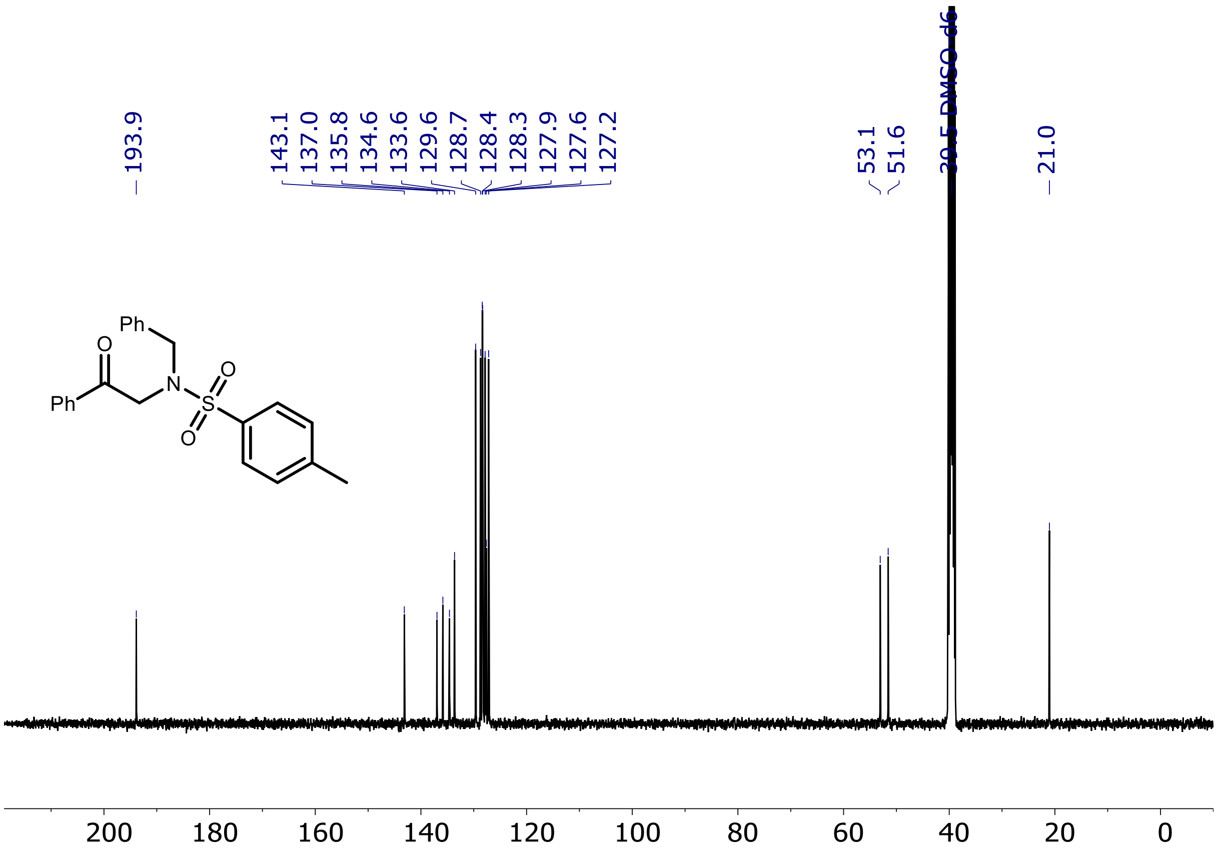

(S5), ^{1}H, $CDCl_3$, 600 MHz

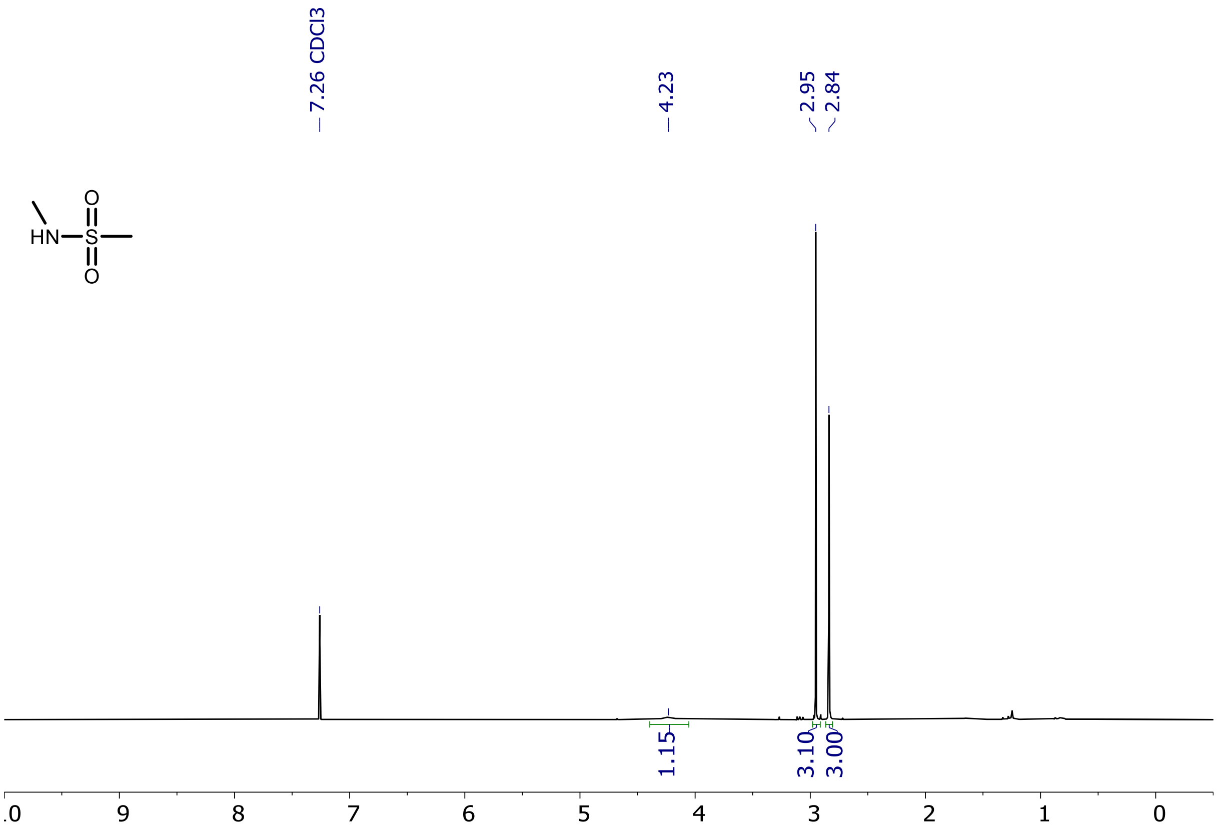

(S5), ^{13}C, $CDCl_3$, 151 MHz

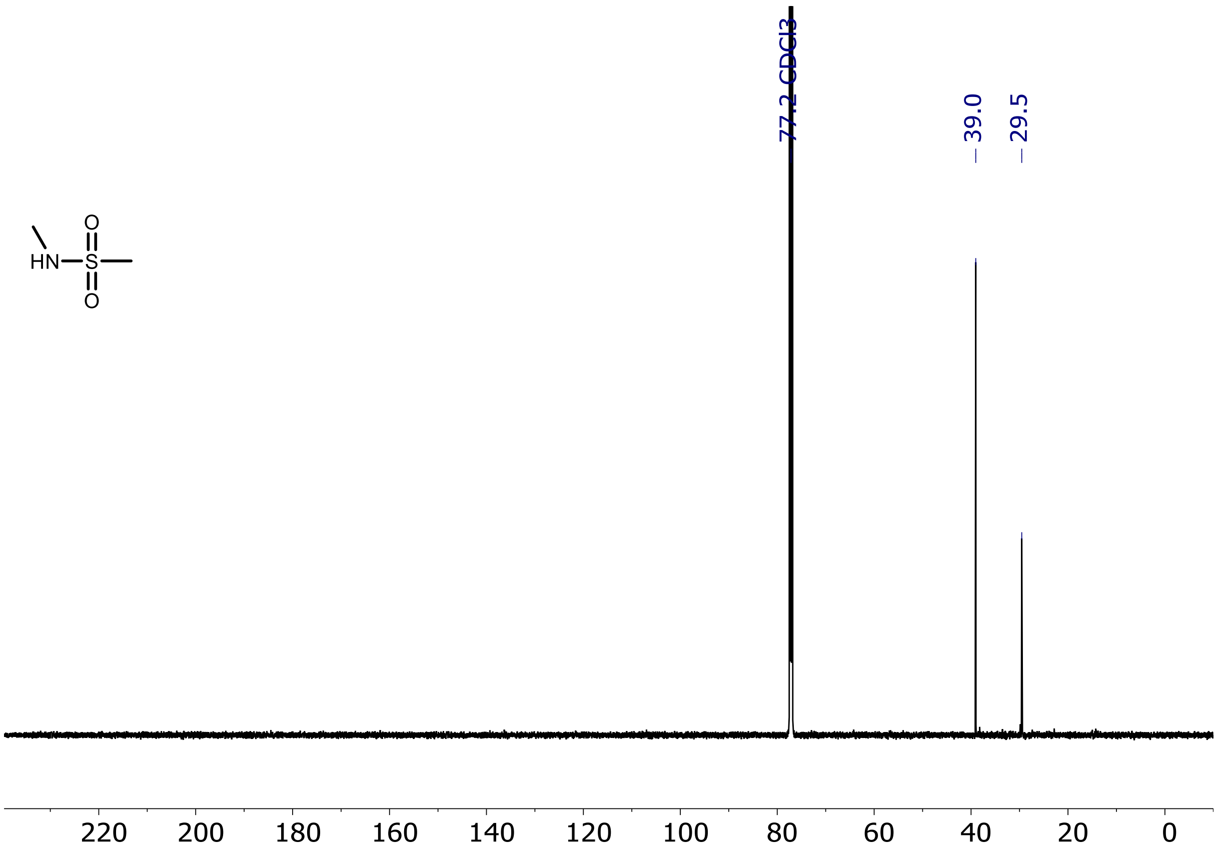

(1e), ^{1}H, CDCl$_3$, 600 MHz

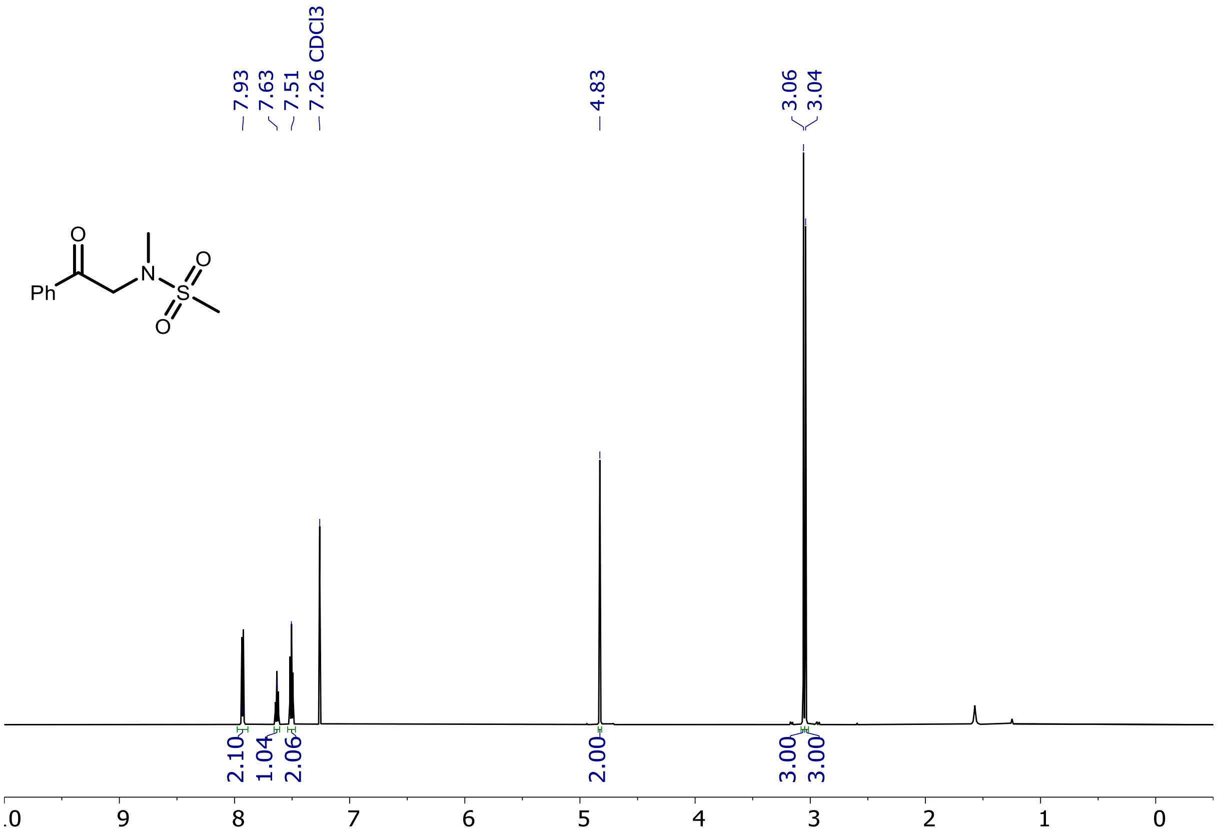

(1e), ^{13}C, CDCl$_3$, 151 MHz

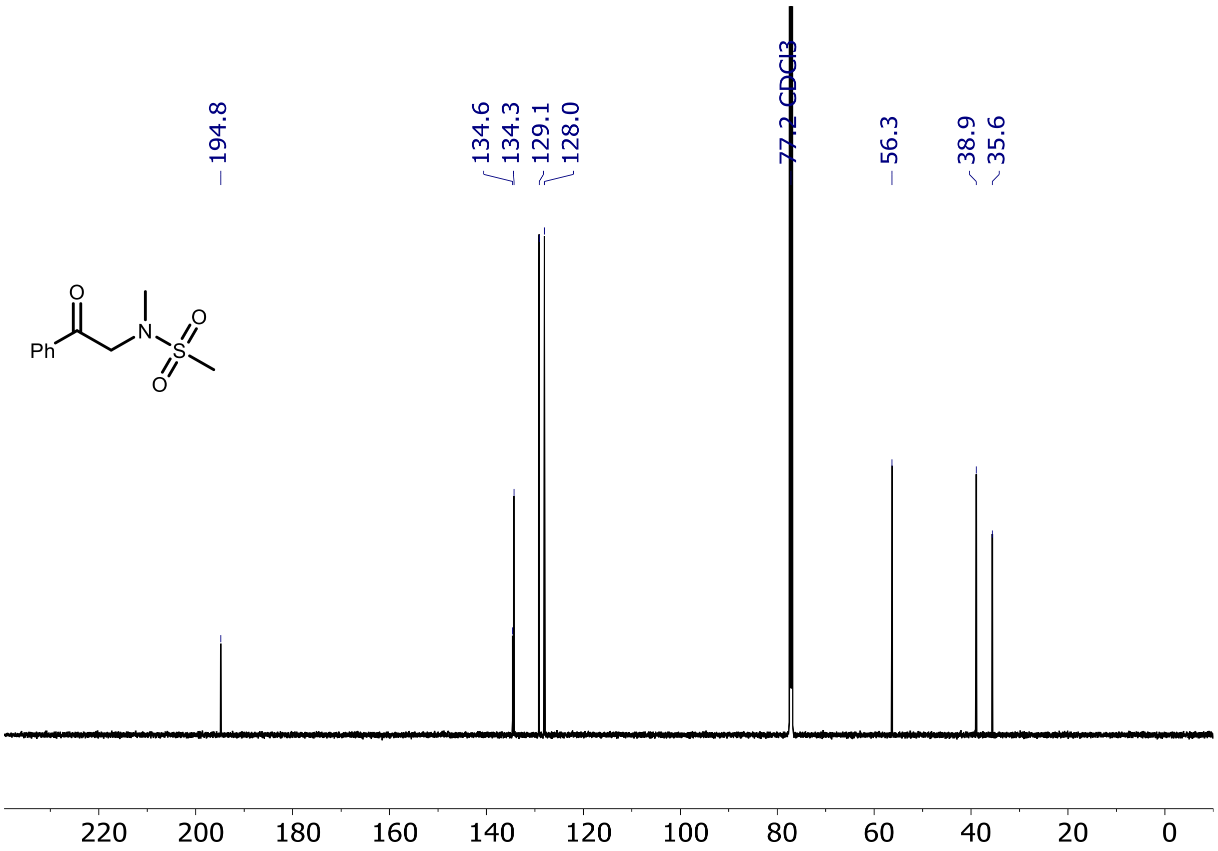

(S6), ^{1}H, $CDCl_3$, 400 MHz

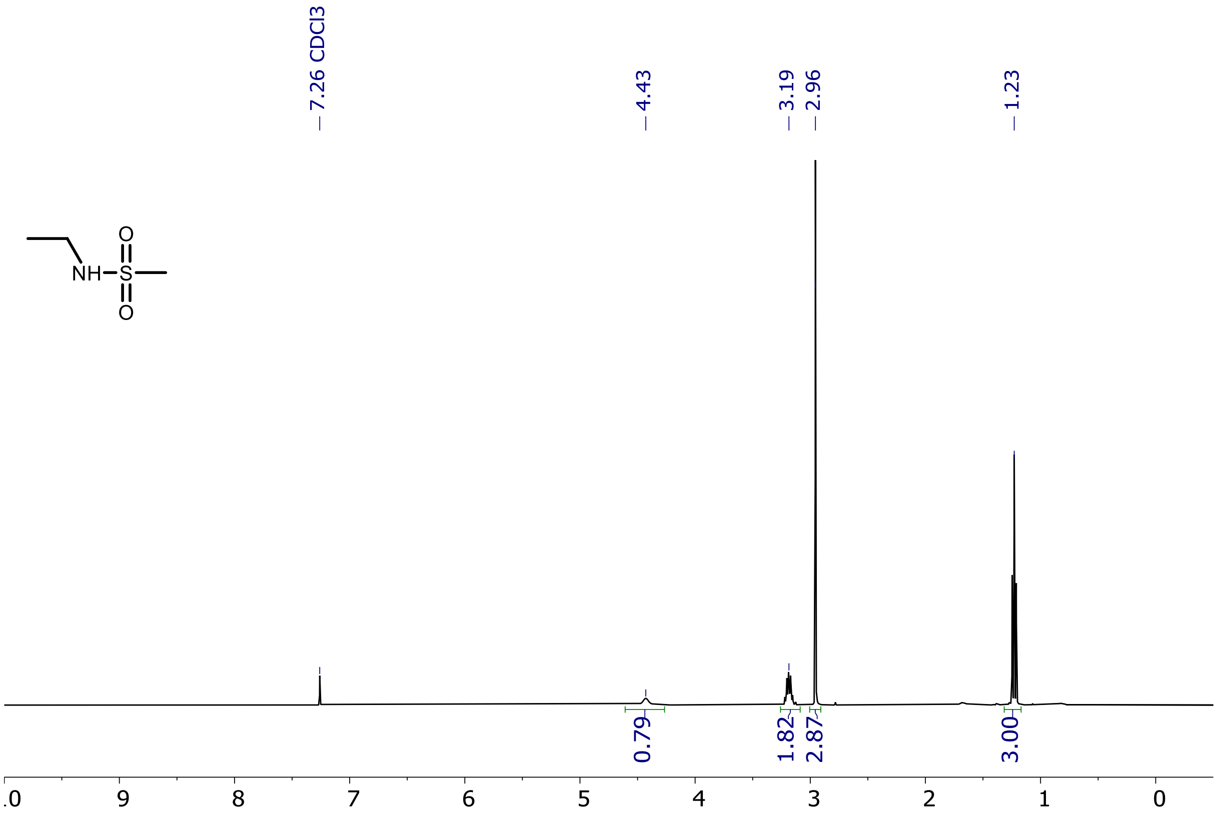

(S6), ^{13}C, $CDCl_3$, 101 MHz

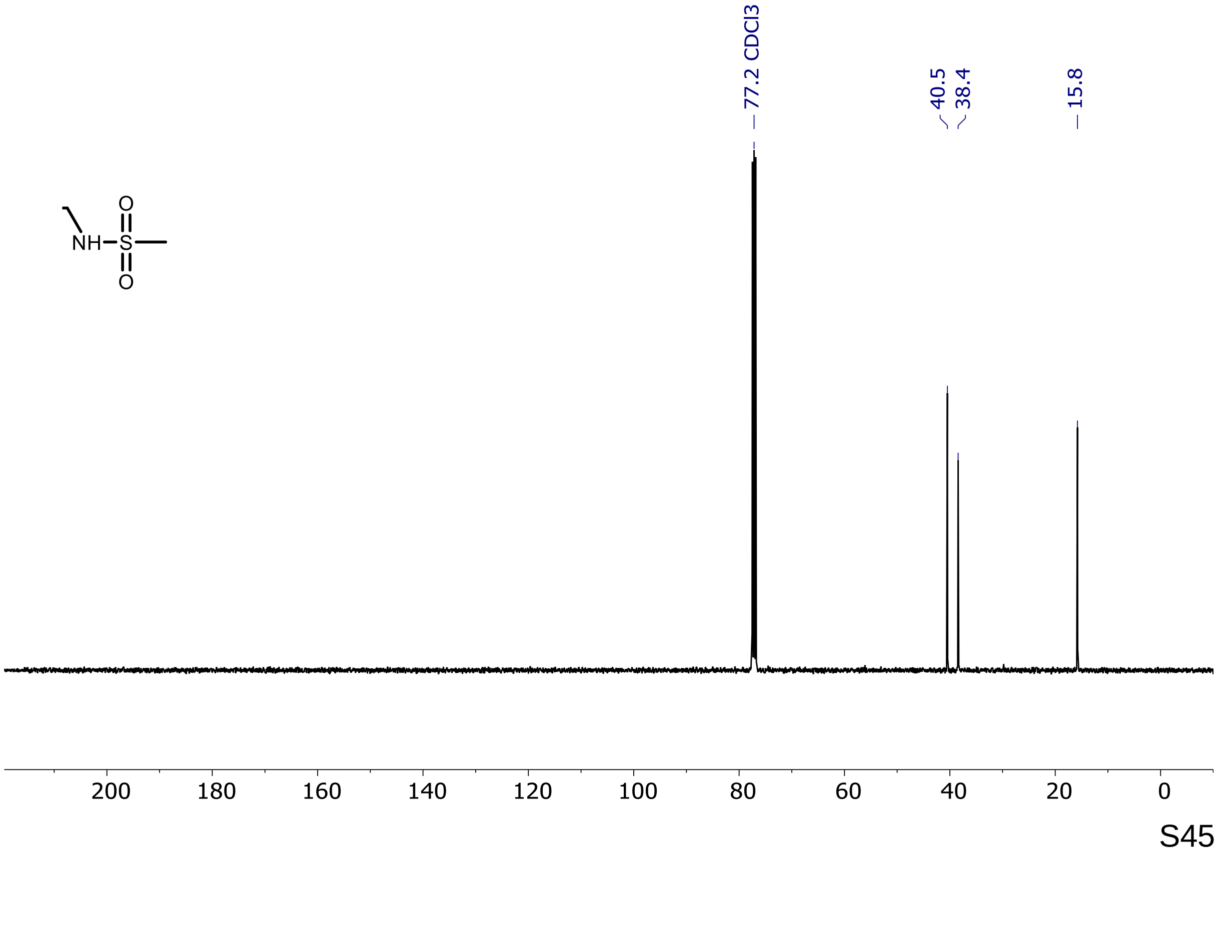

(1f), ^{1}H, $CDCl_3$, 600 MHz

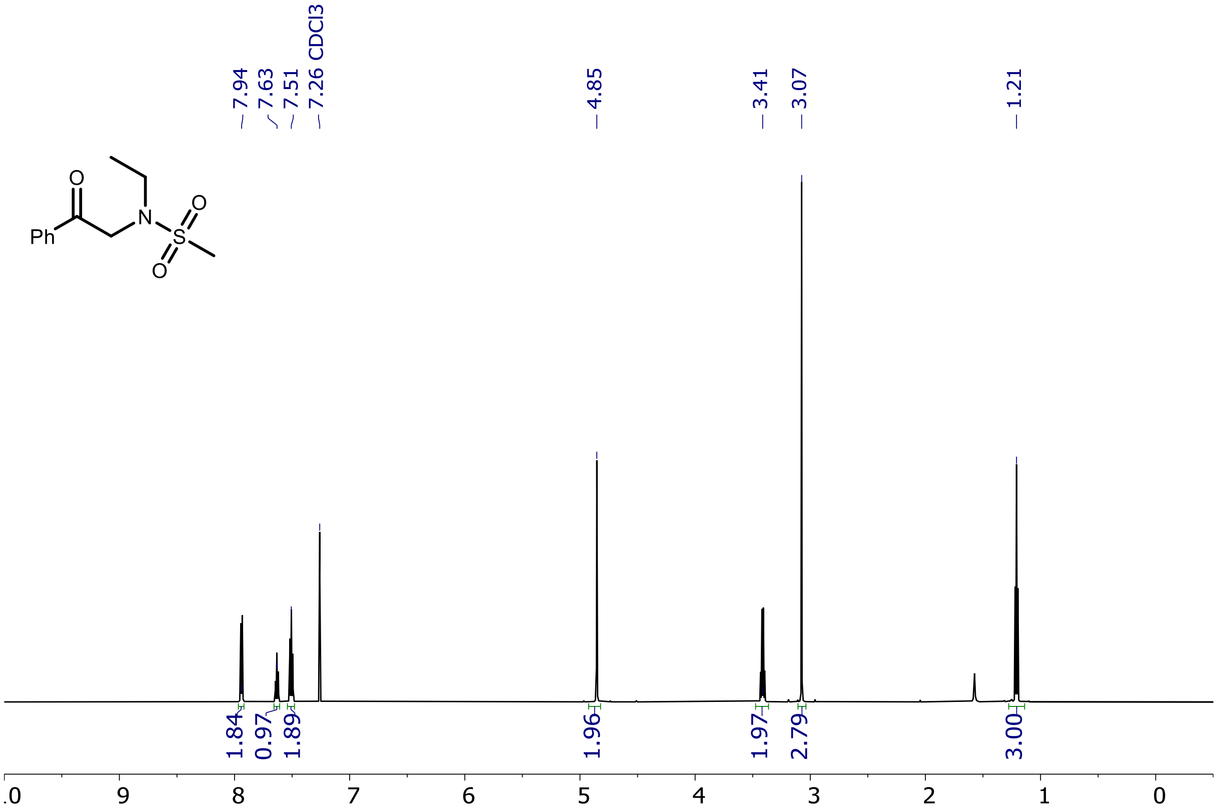

(1f), ^{13}C, $CDCl_3$, 151 MHz

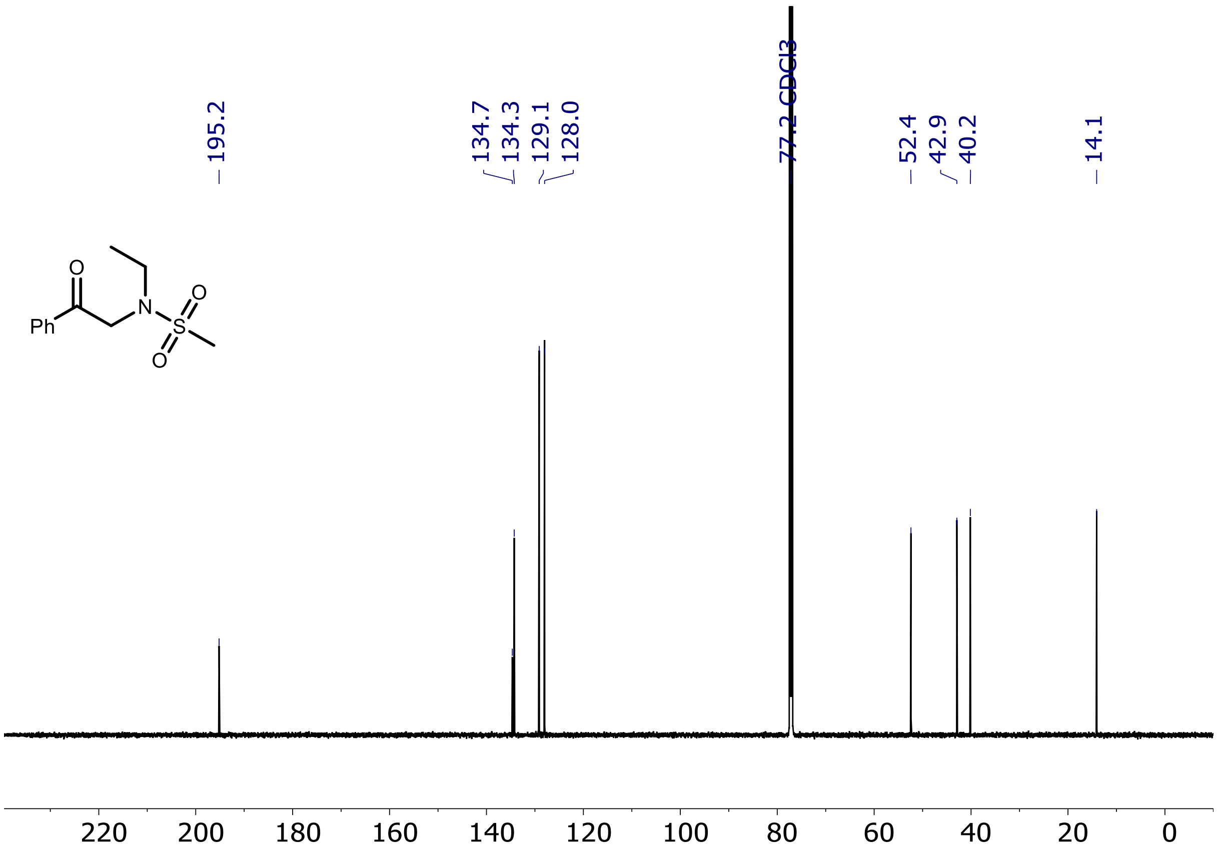

(S7), ^{1}H, $CDCl_3$, 400 MHz

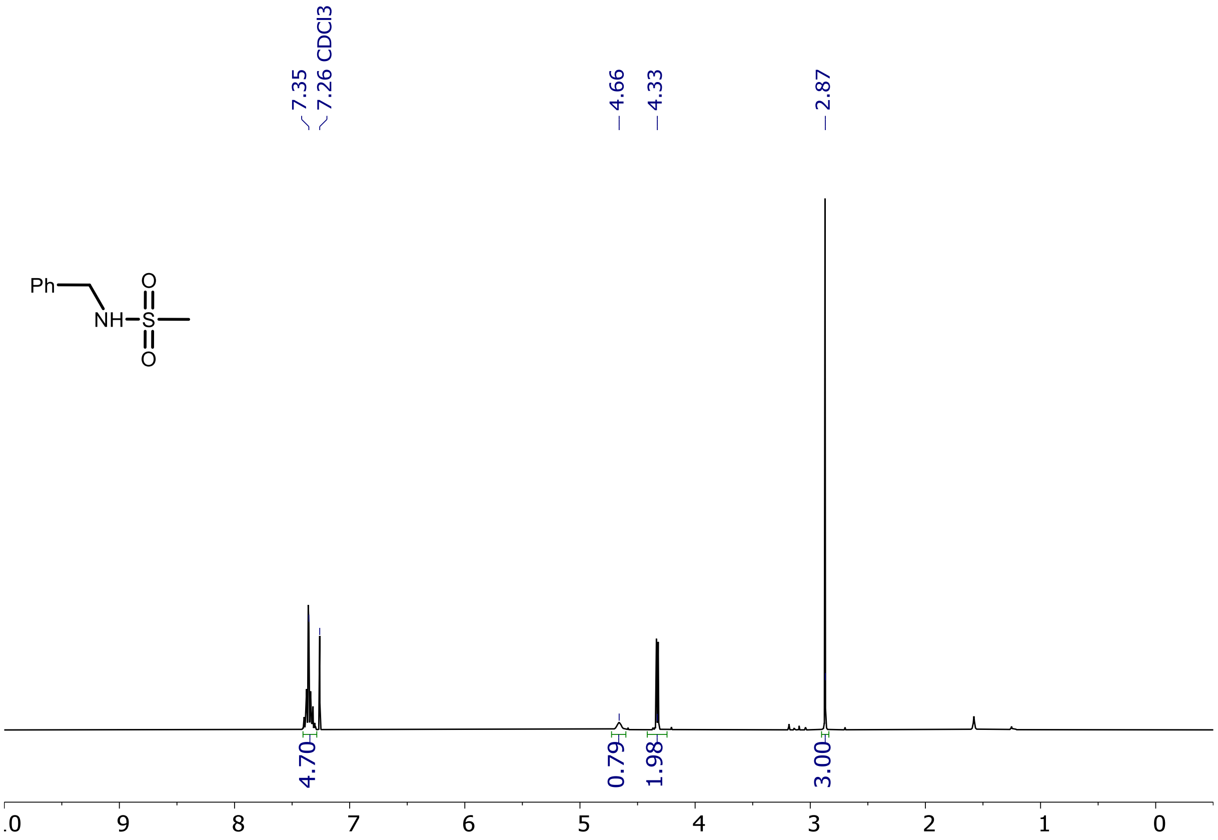

(S7), ^{13}C, $CDCl_3$, 101 MHz

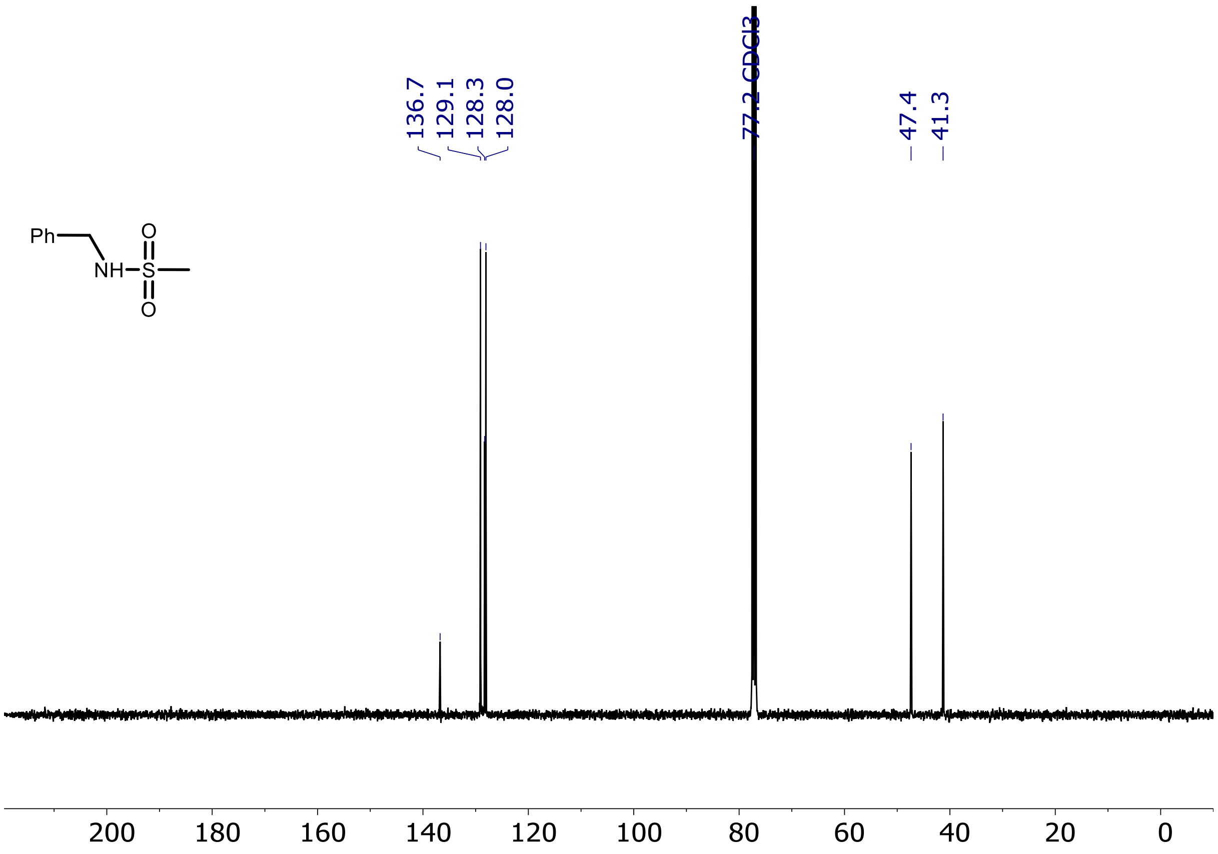

(1g), ^{1}H, $CDCl_3$, 400 MHz

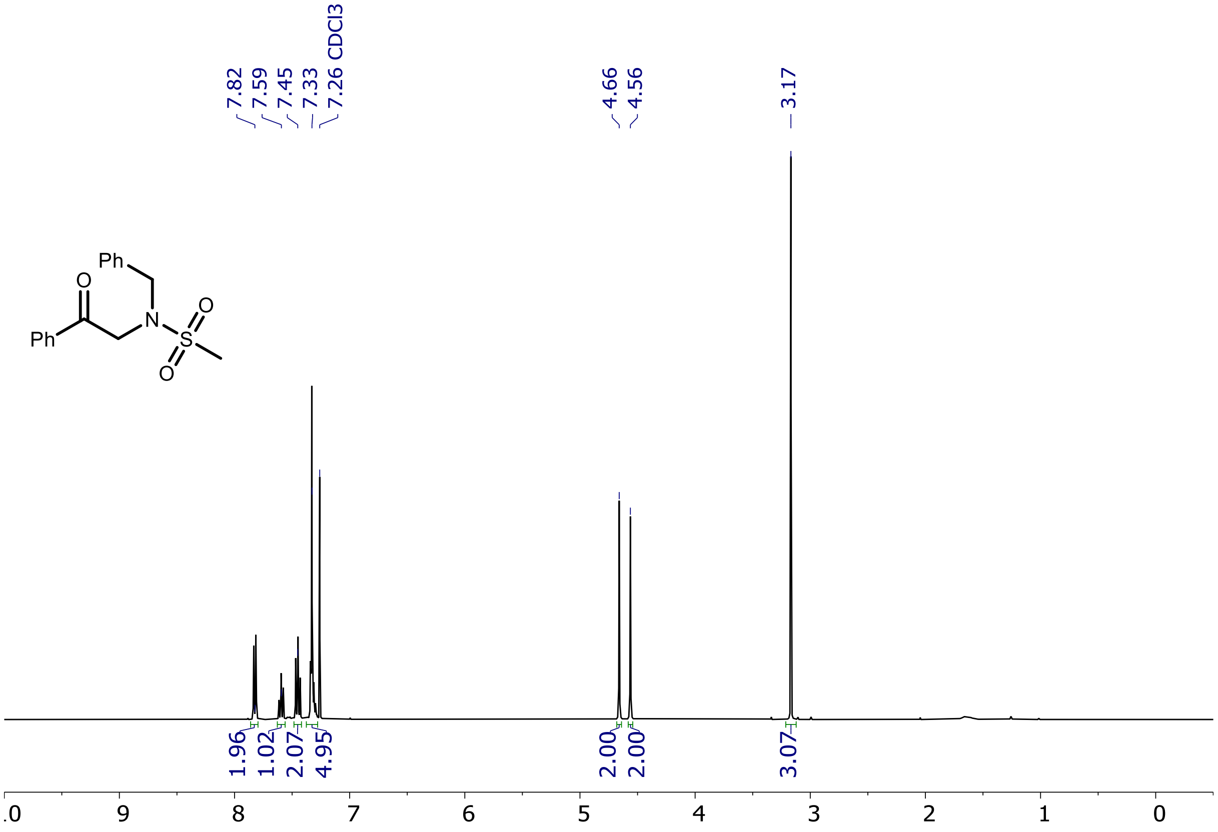

(1g), ^{13}C, $CDCl_3$, 101 MHz

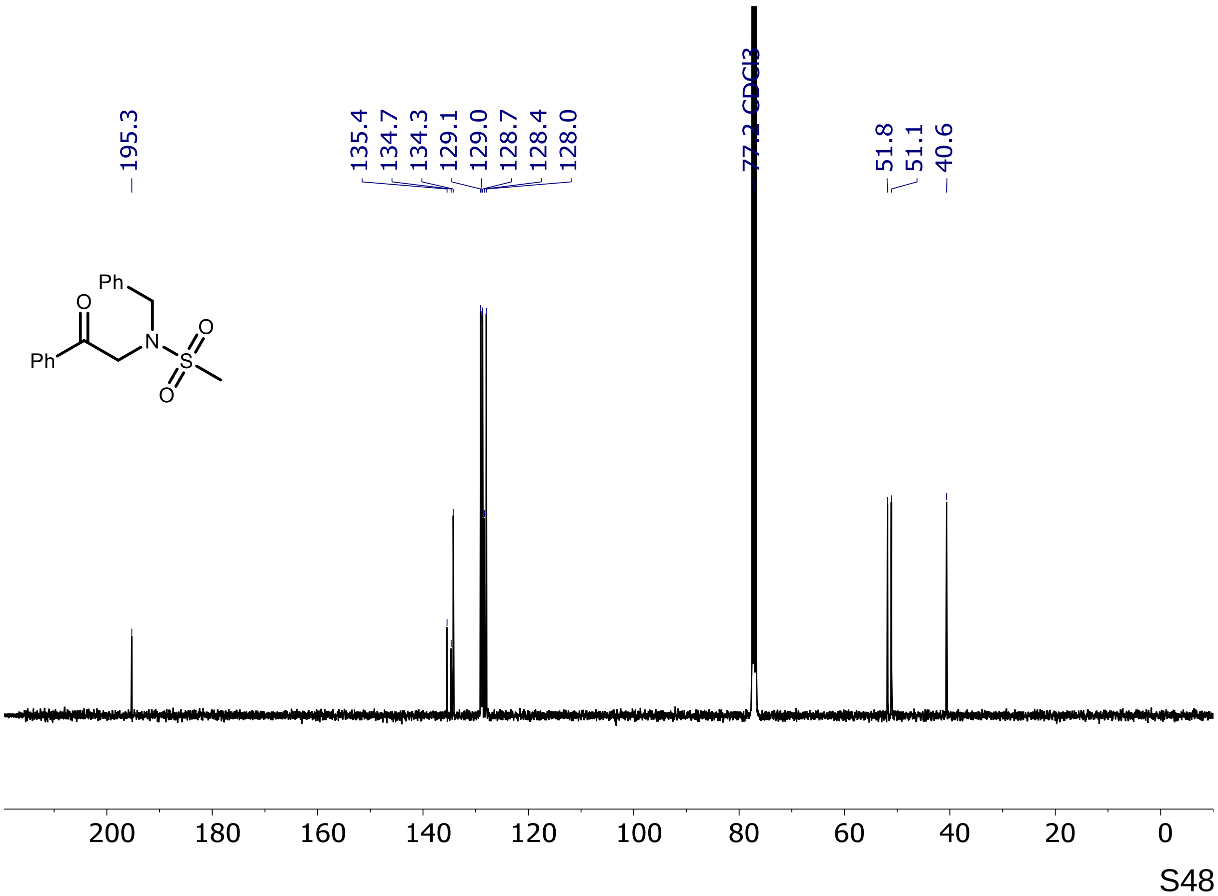

(1h), ^{1}H, $CDCl_3$, 600 MHz

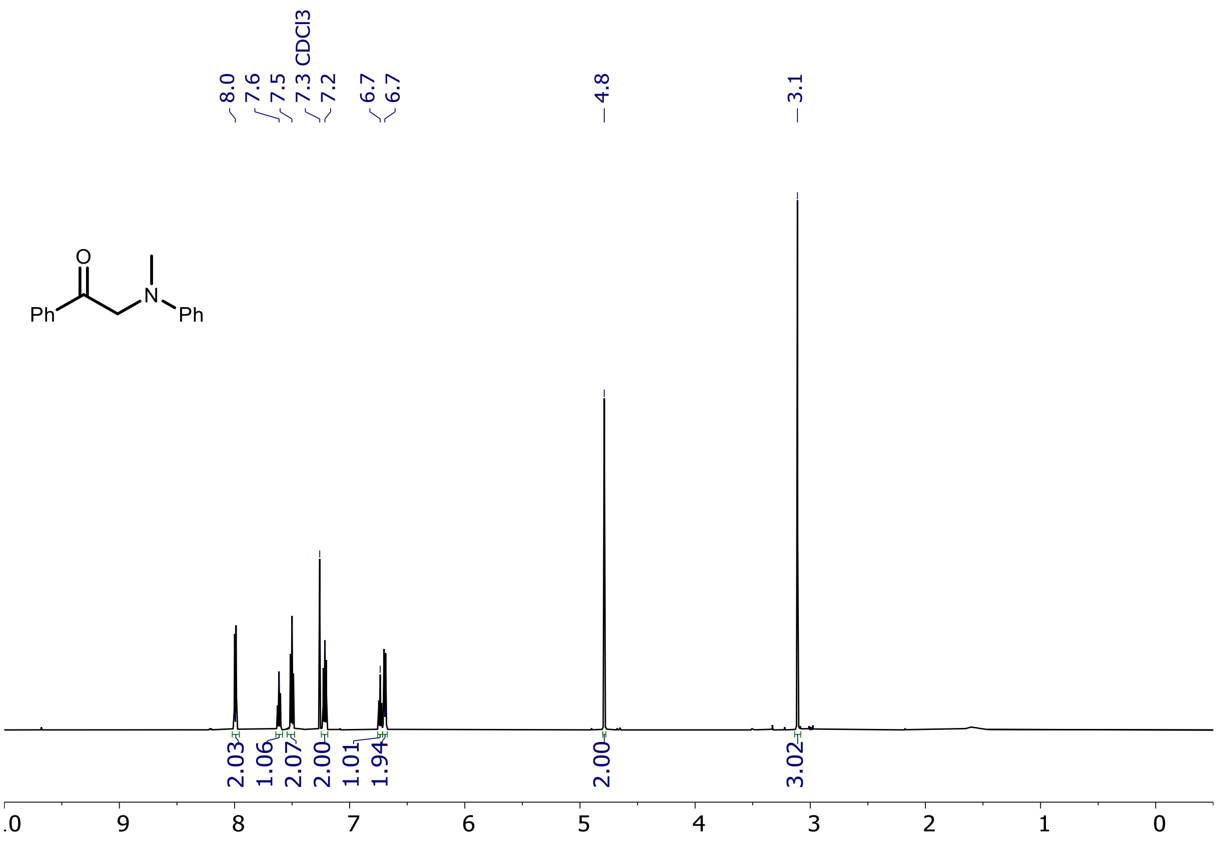

(1h), ^{13}C, $CDCl_3$, 151 MHz

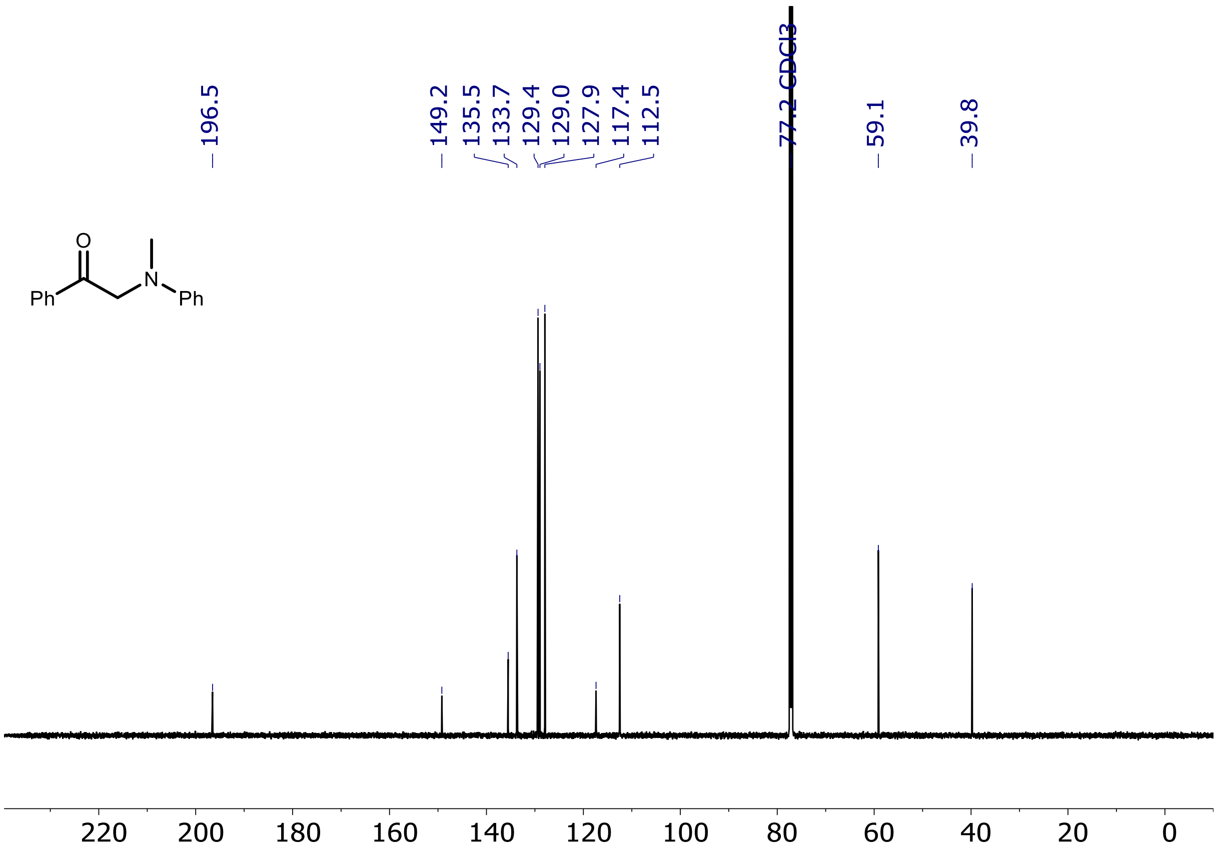

(1i), ^{1}H, $CDCl_3$, 400 MHz

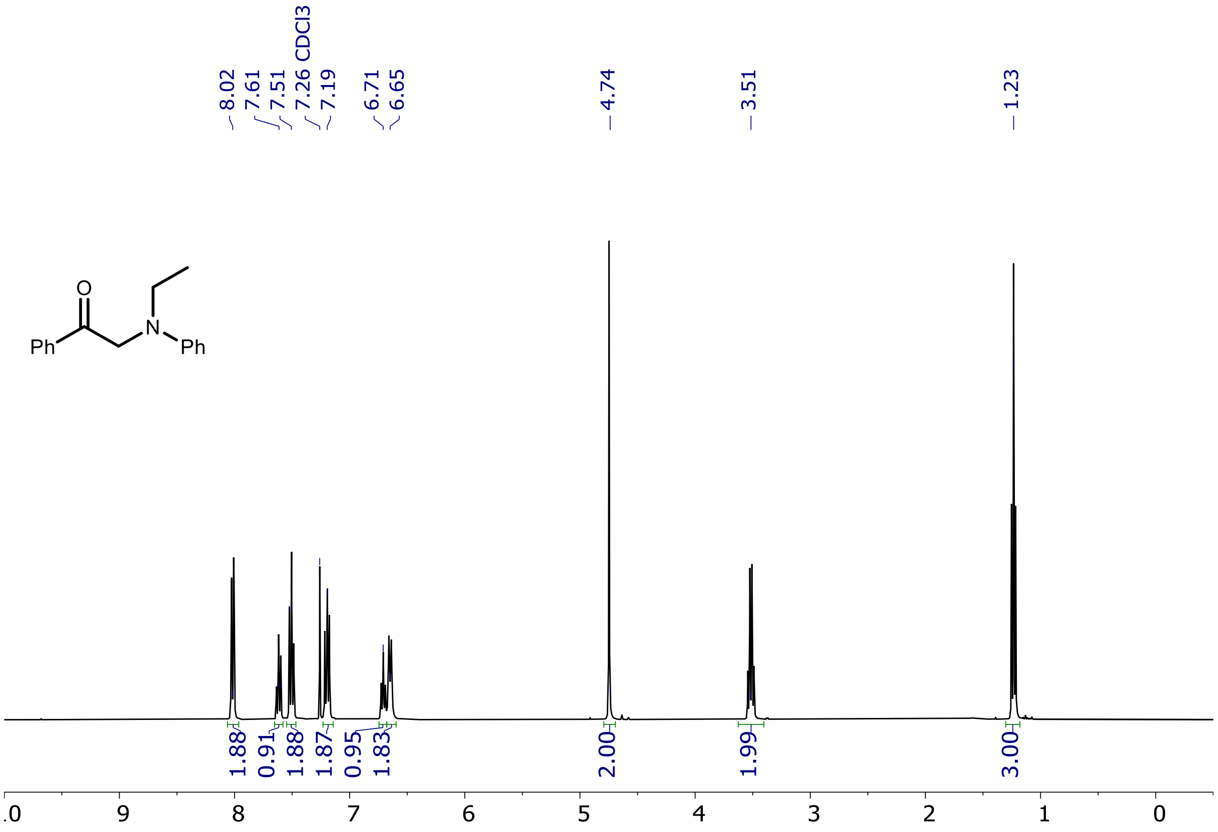

(1i), ^{13}C, $CDCl_3$, 101 MHz

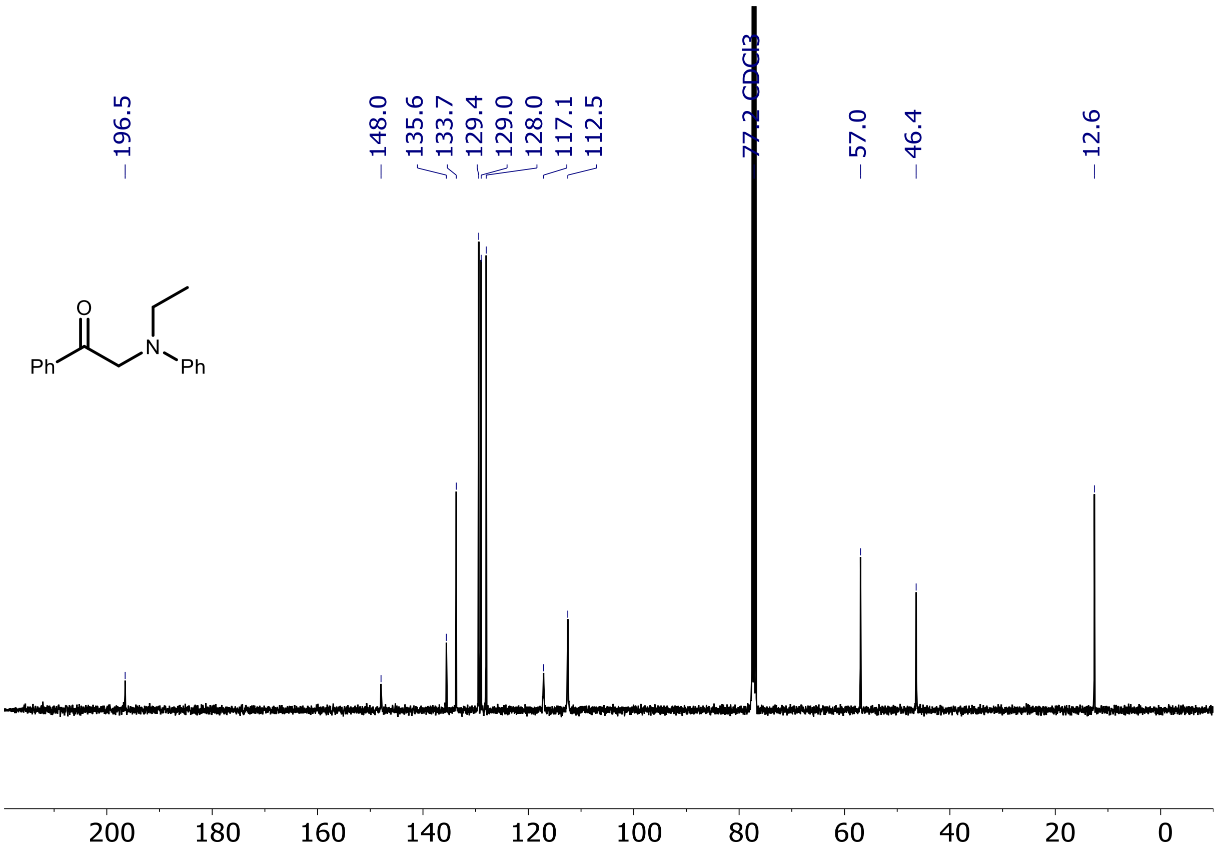

(1j), ^{1}H, $CDCl_3$, 300 MHz

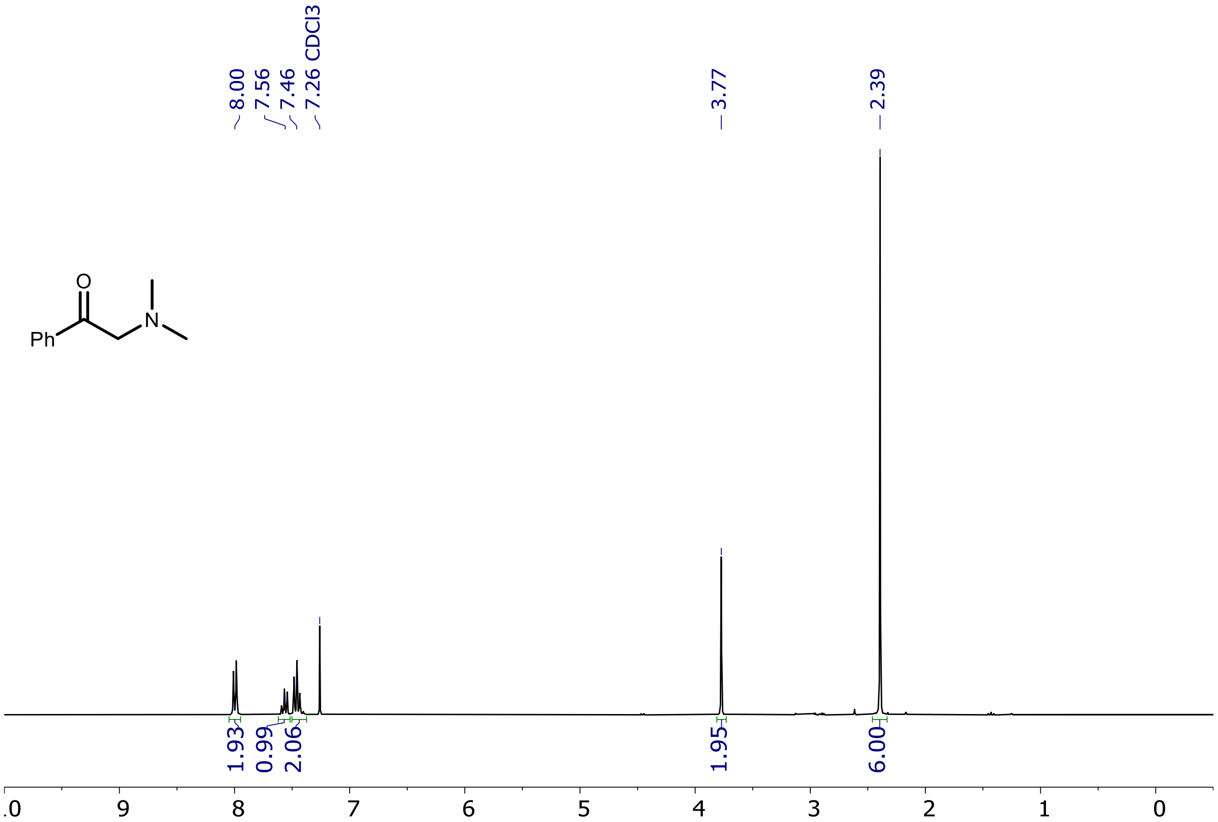

(1j), ^{13}C, $CDCl_3$, 101 MHz

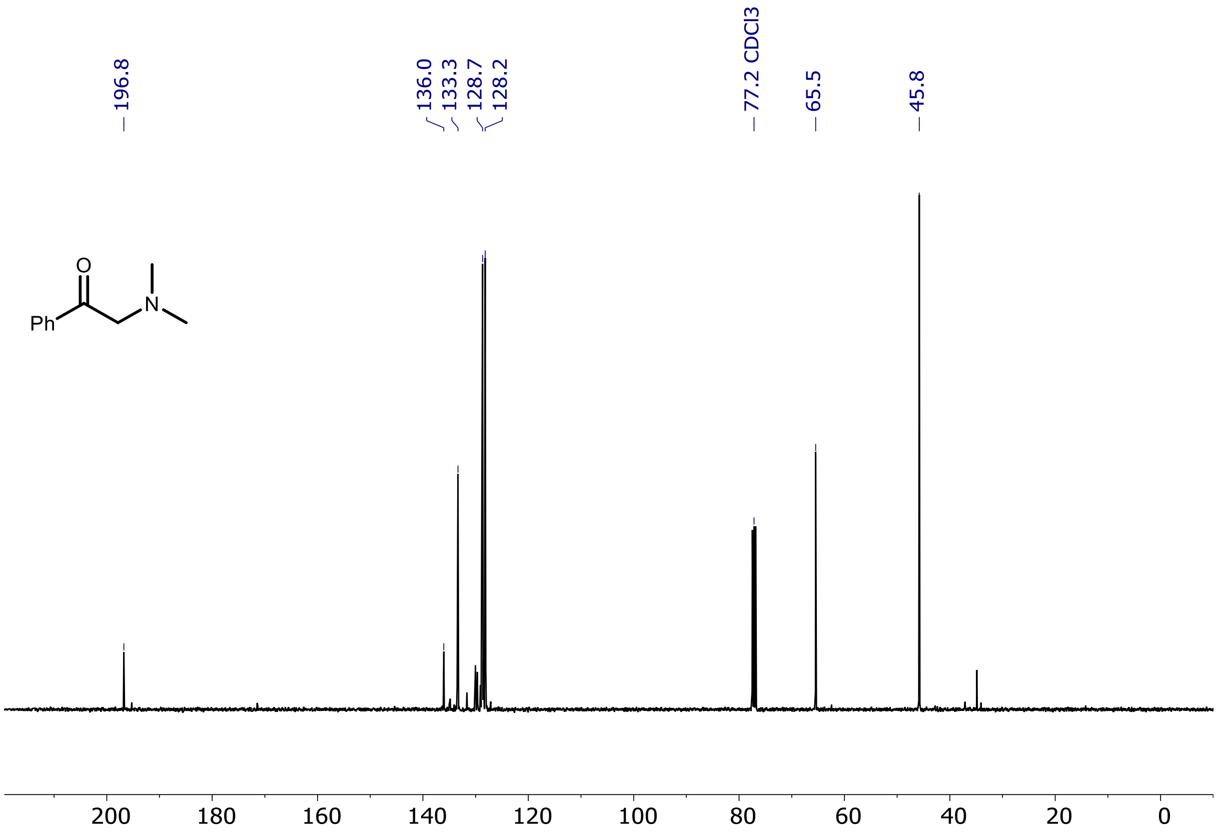

(1k), ^{1}H, $CDCl_3$, 300 MHz

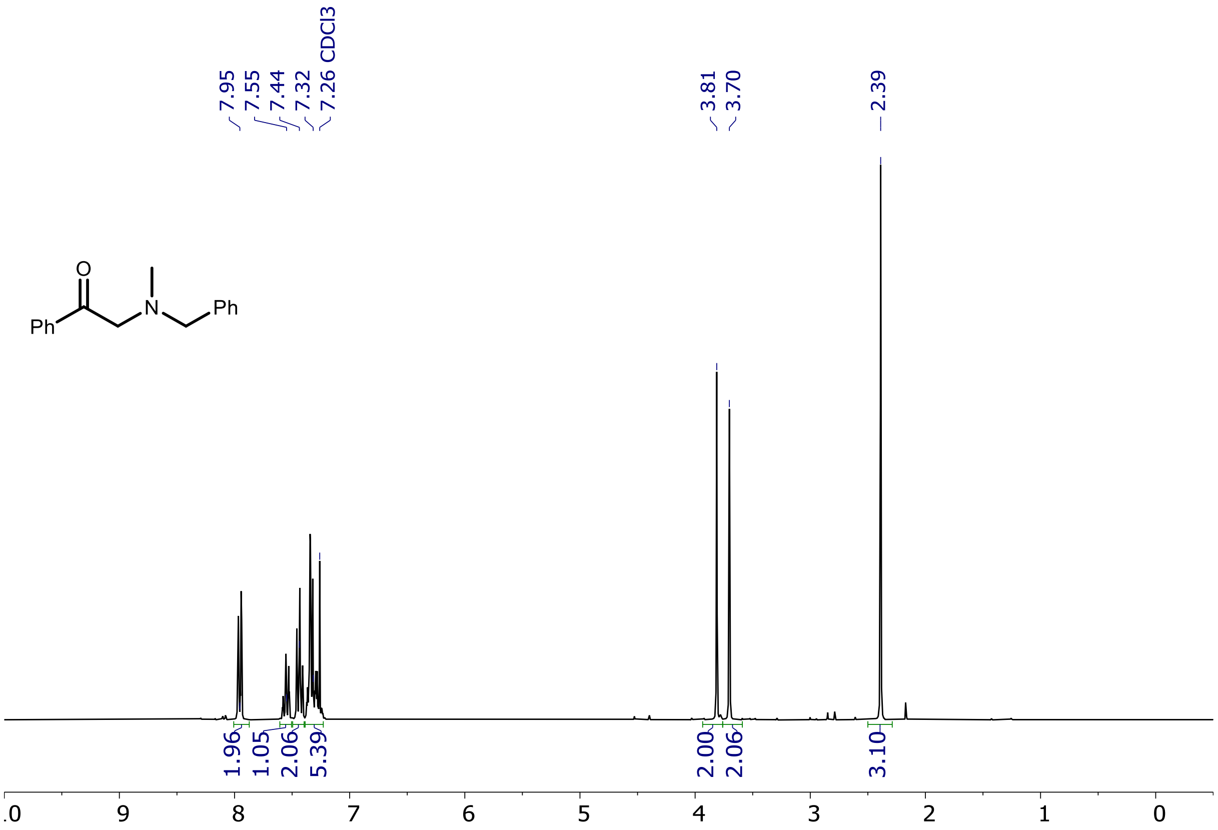

(1k), ^{13}C, $CDCl_3$, 101 MHz

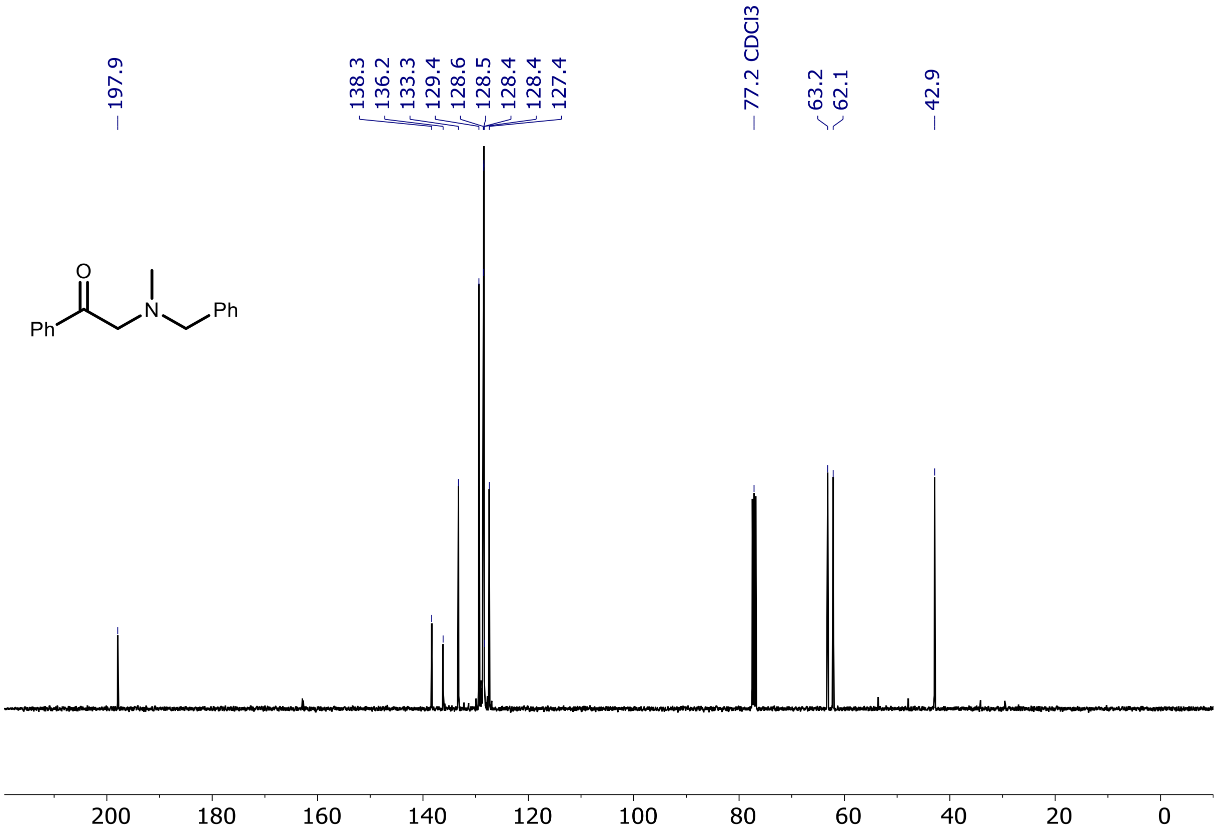

(1l), ^{1}H, CDCl$_3$, 400 MHz

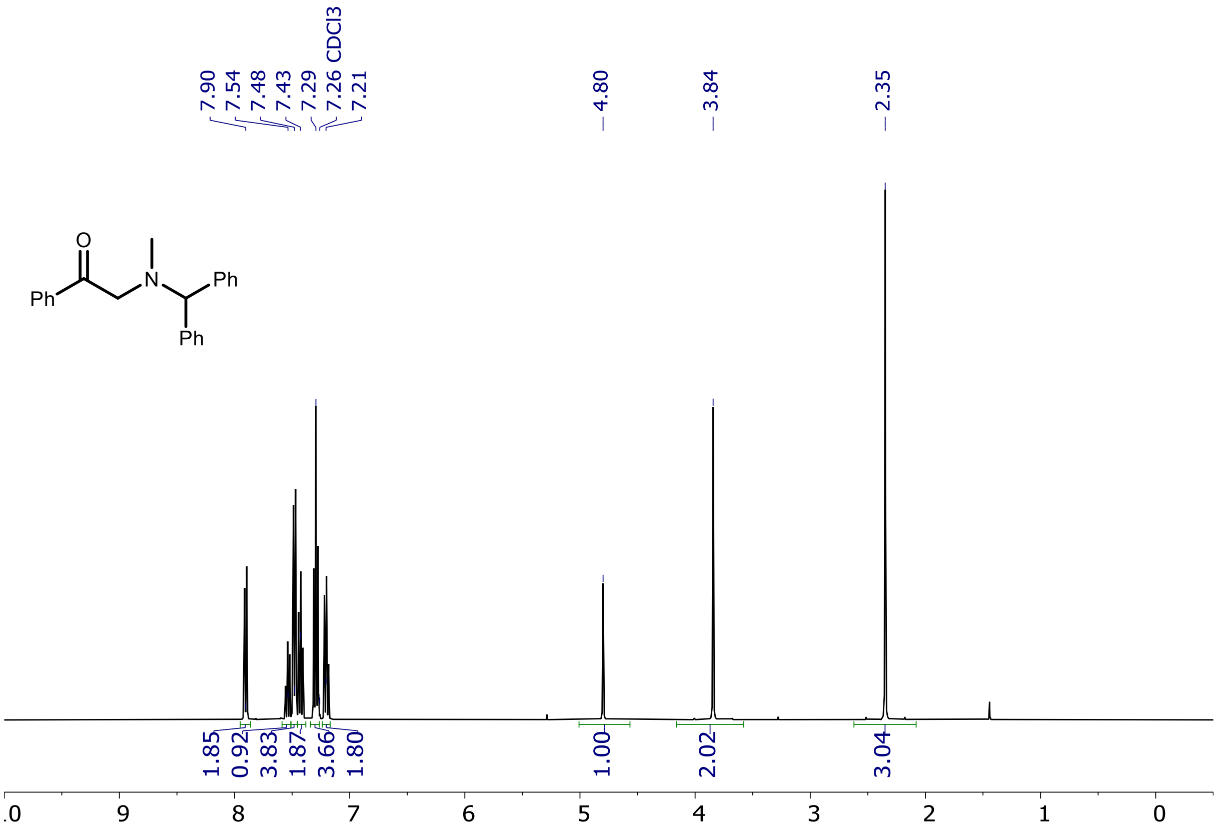

(1l), ^{13}C, CDCl$_3$, 101 MHz

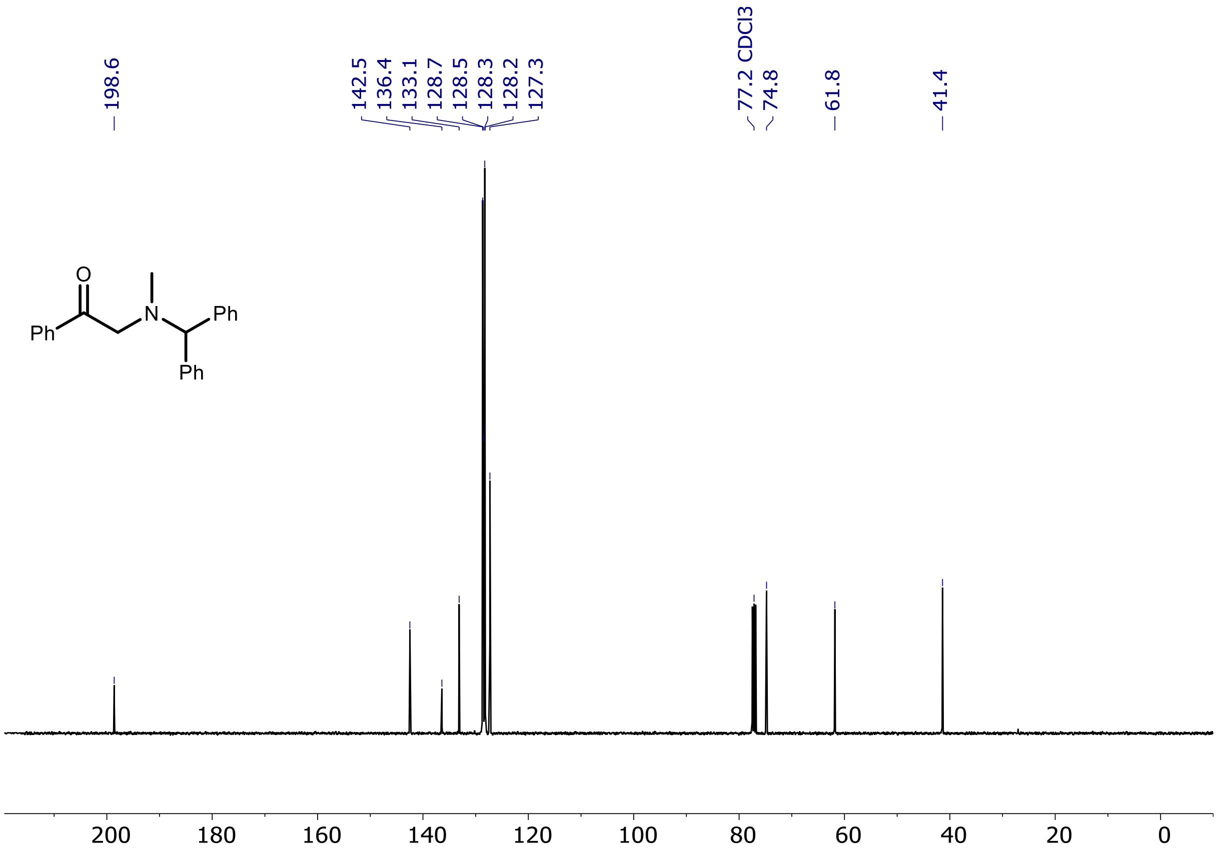

(S8), ^{1}H, DMSO-d6, 400 MHz

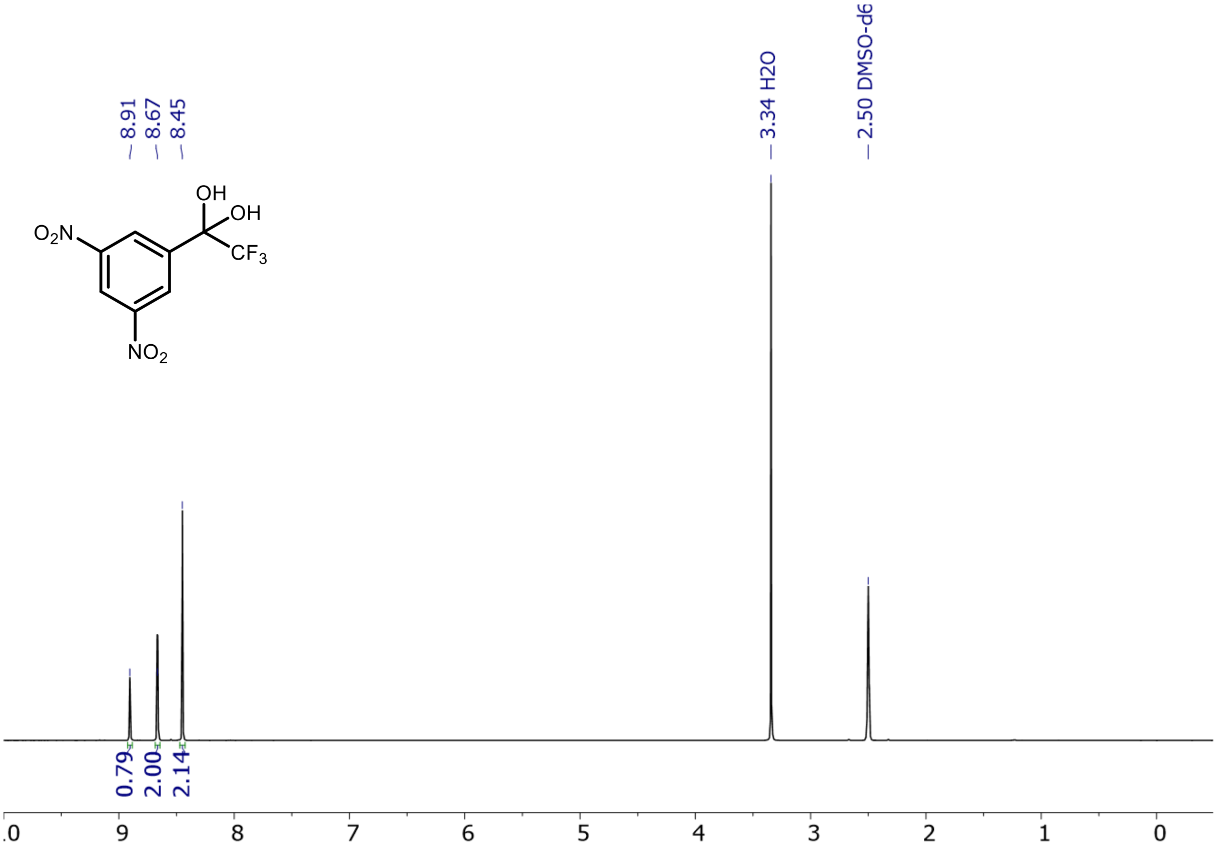

(S8), ^{13}C, DMSO-d6, 101 MHz

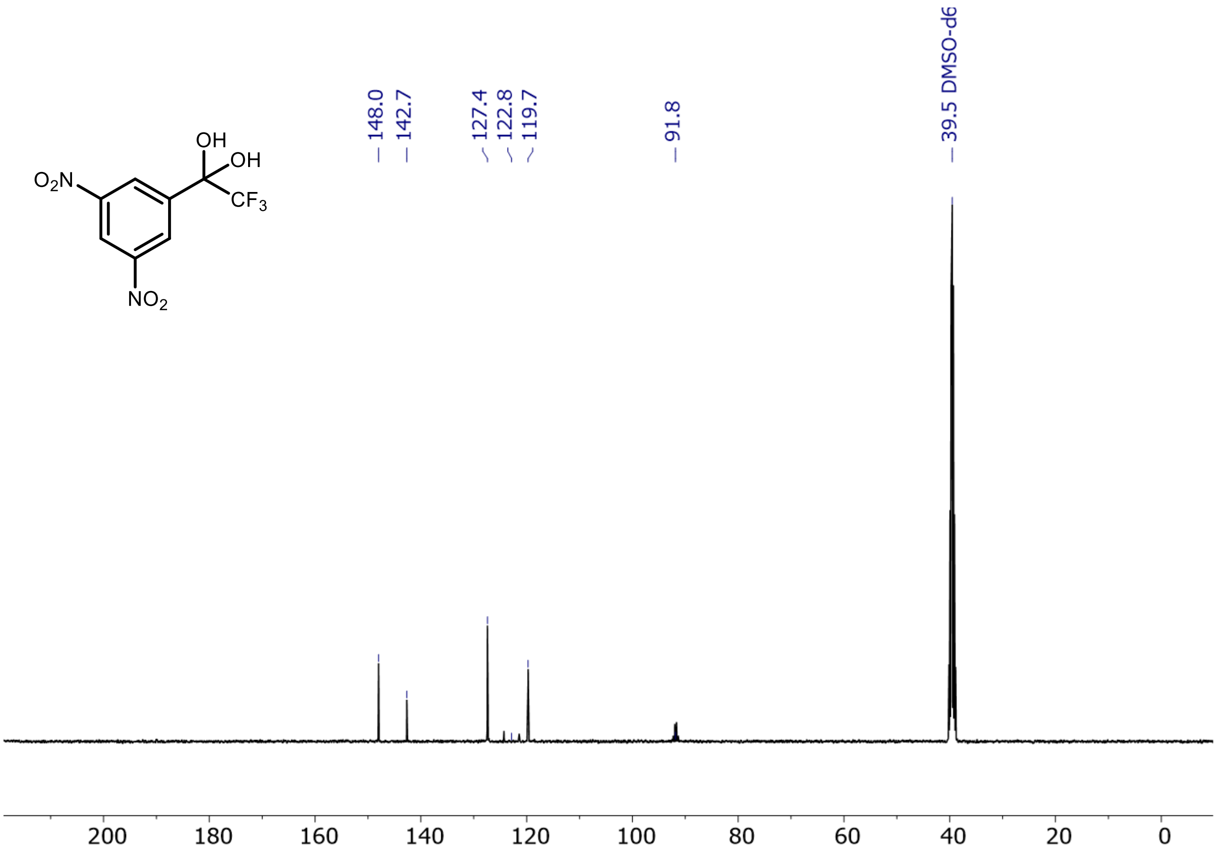

(S8), ^{19}F, DMSO-d$_6$, 376 MHz

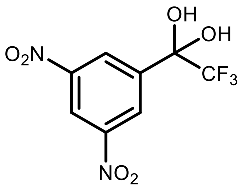

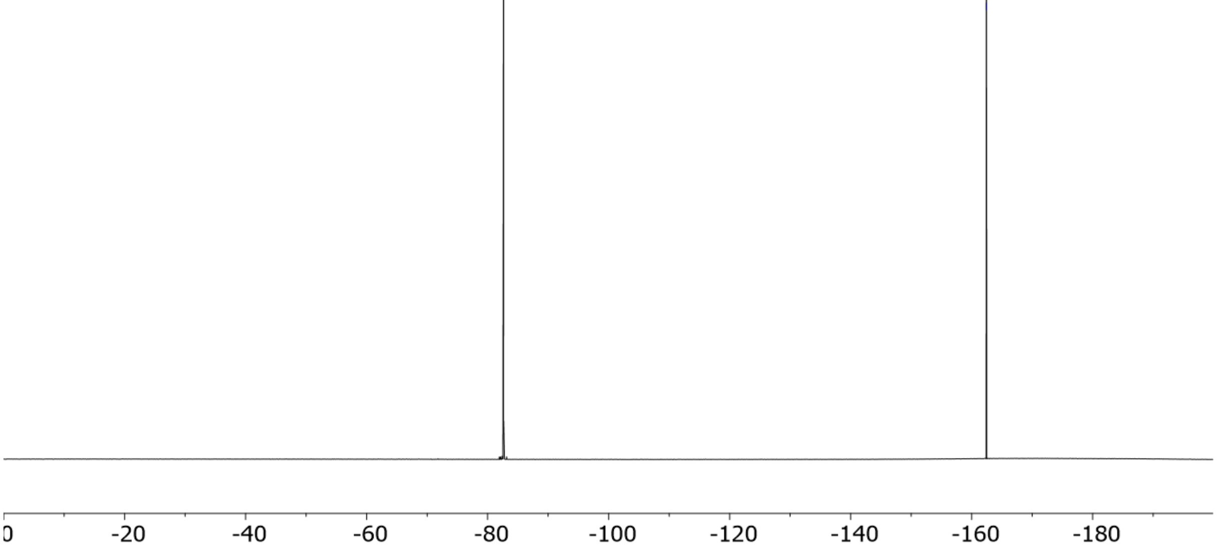

(7), ^{1}H, CDCl$_3$, 400 MHz

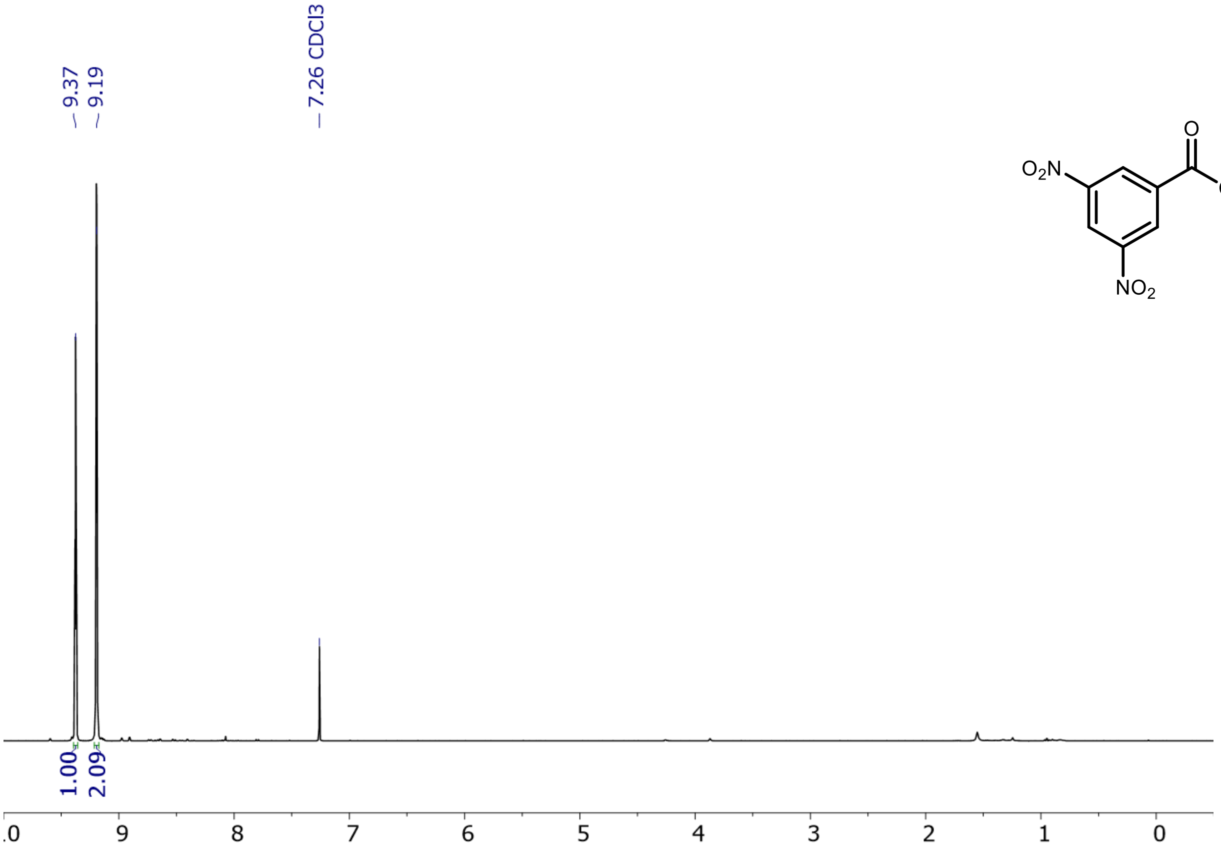

(7), ^{13}C, CDCl$_3$, 101 MHz

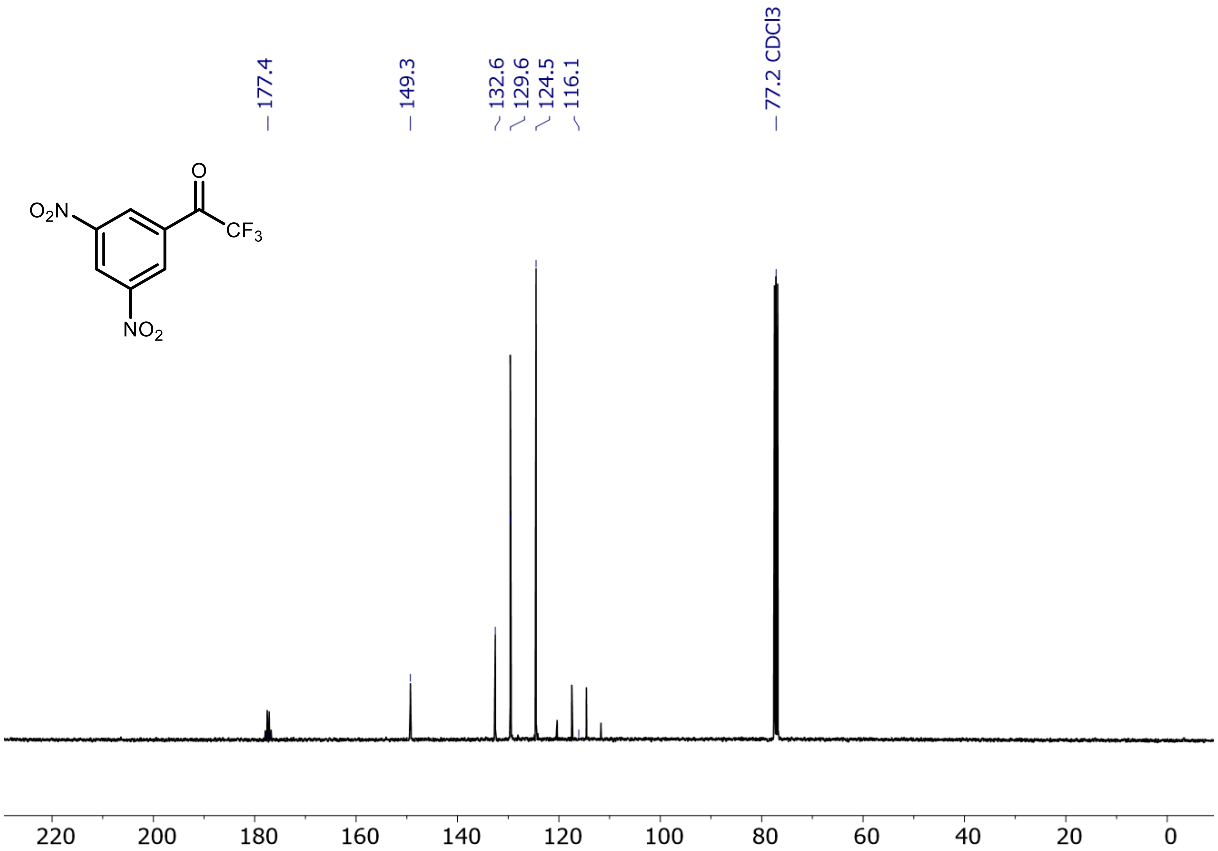

(7), ^{19}F, $CDCl_3$, 377 MHz

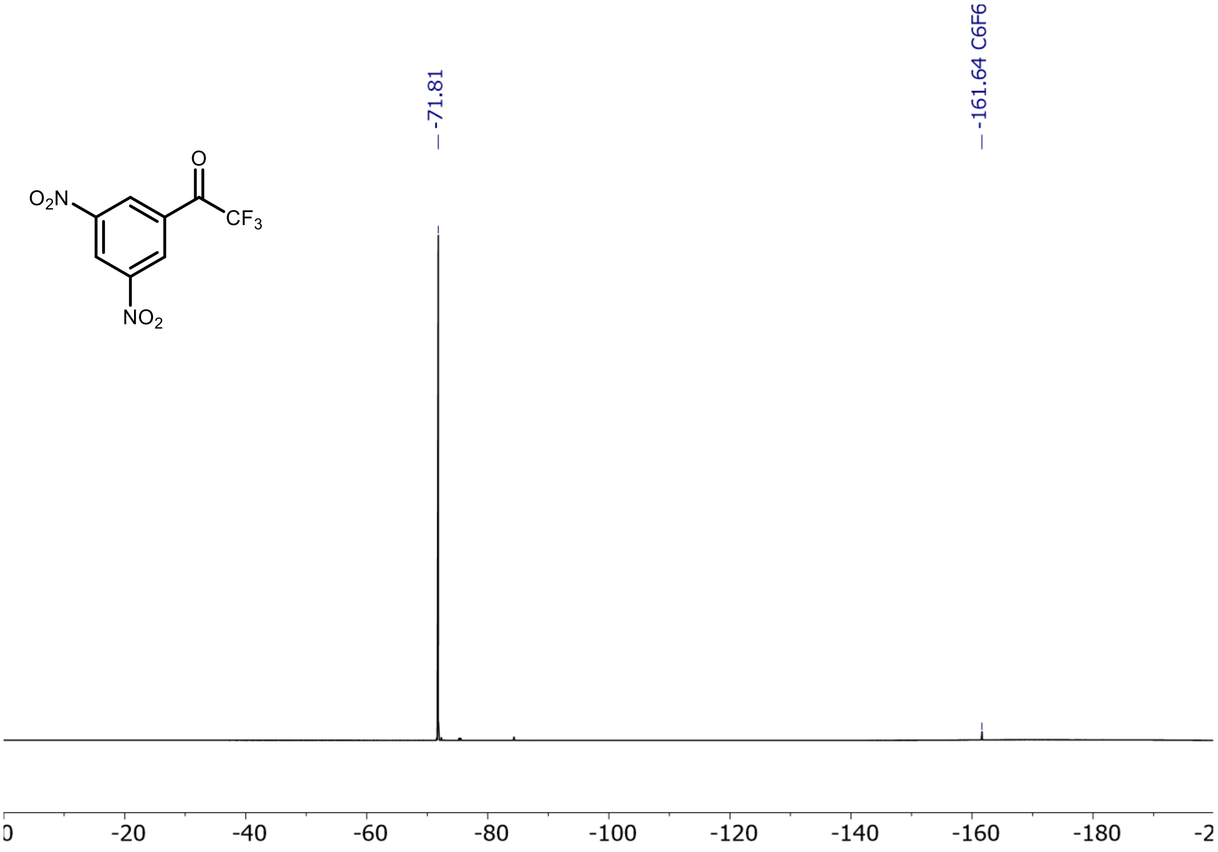

(3a), ^{1}H, DMSO-d_6, 600 MHz

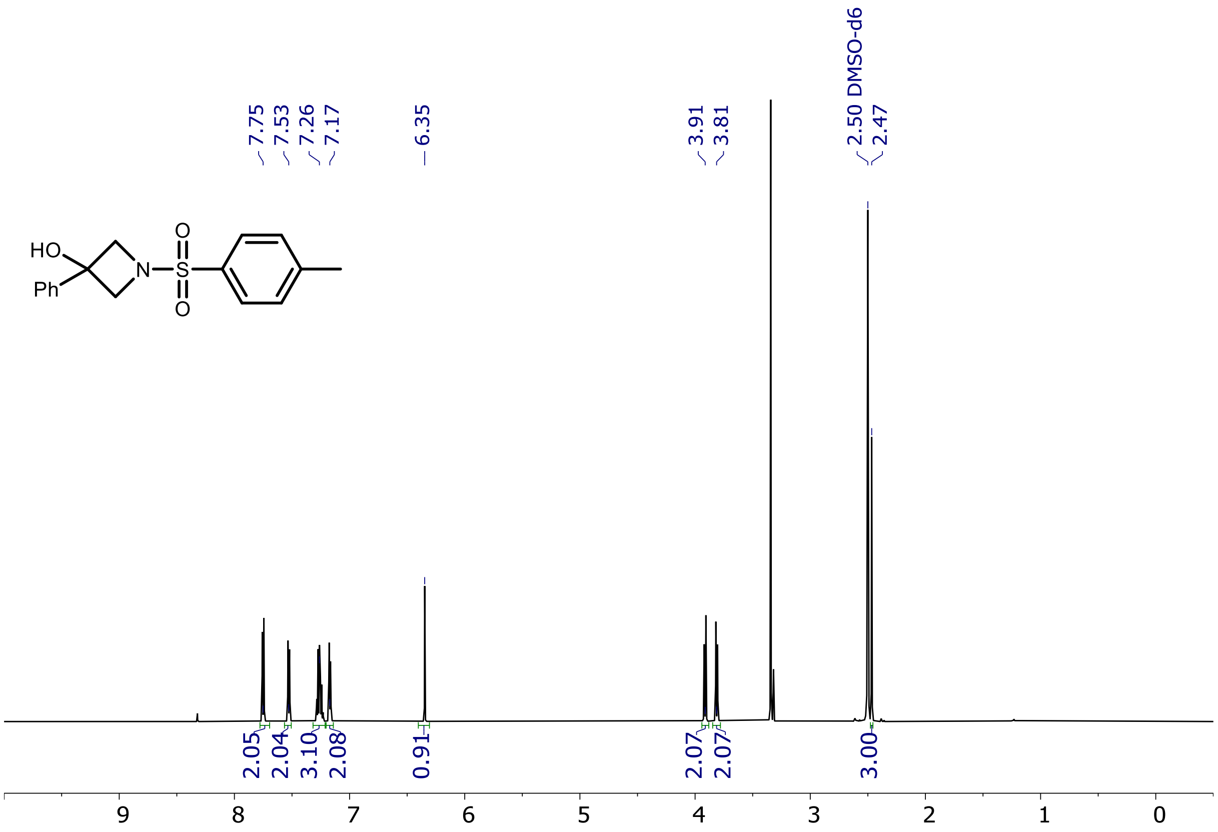

(3a), ^{13}C, DMSO-d_6, 151 MHz

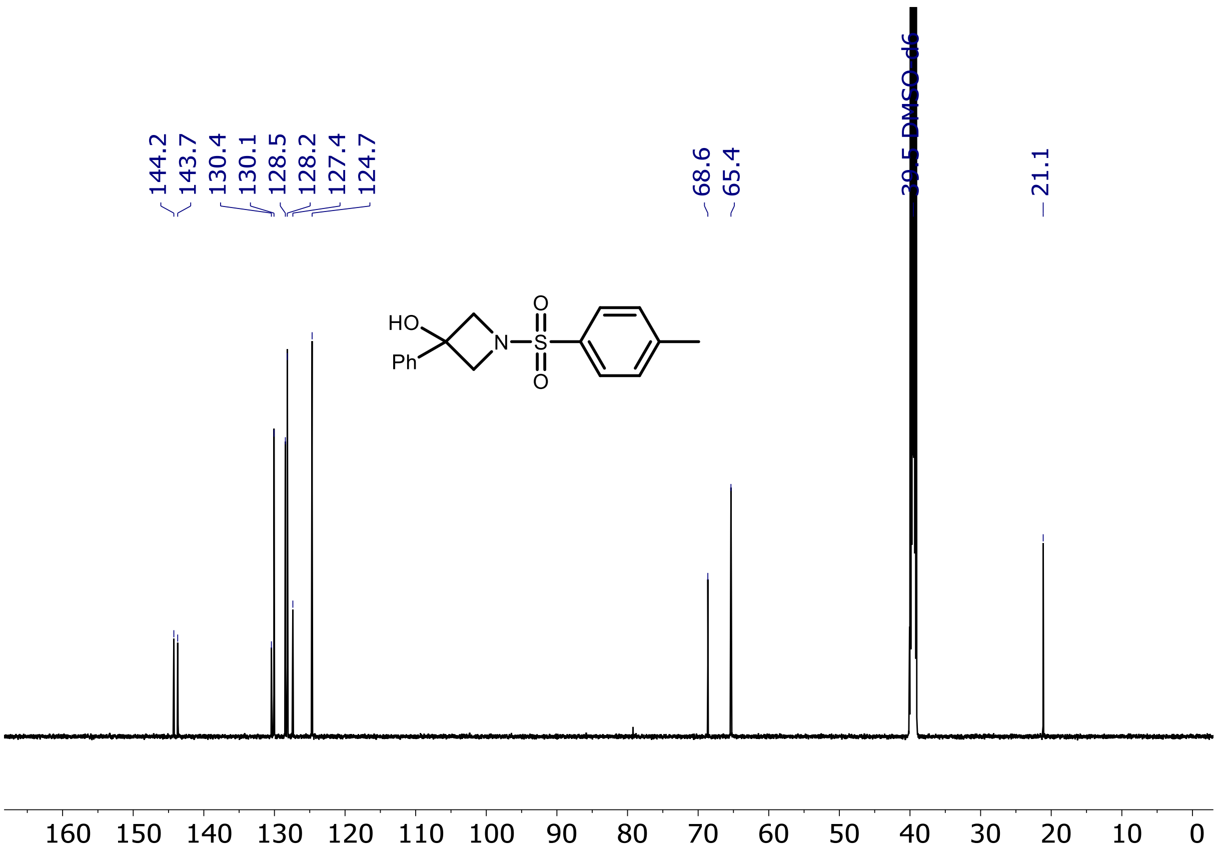

(3b), ^{1}H, DMSO-d_6, 600 MHz

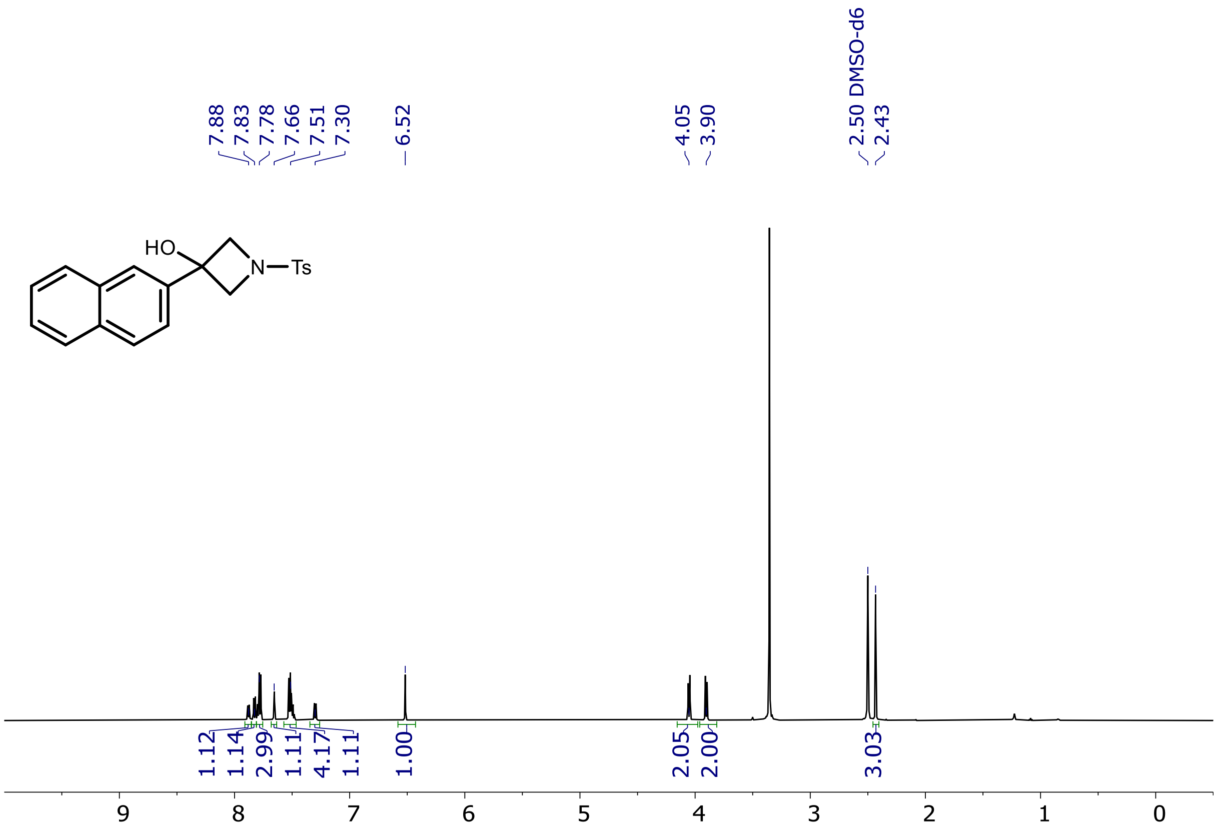

(3b), ^{13}C, DMSO-d_6, 151 MHz

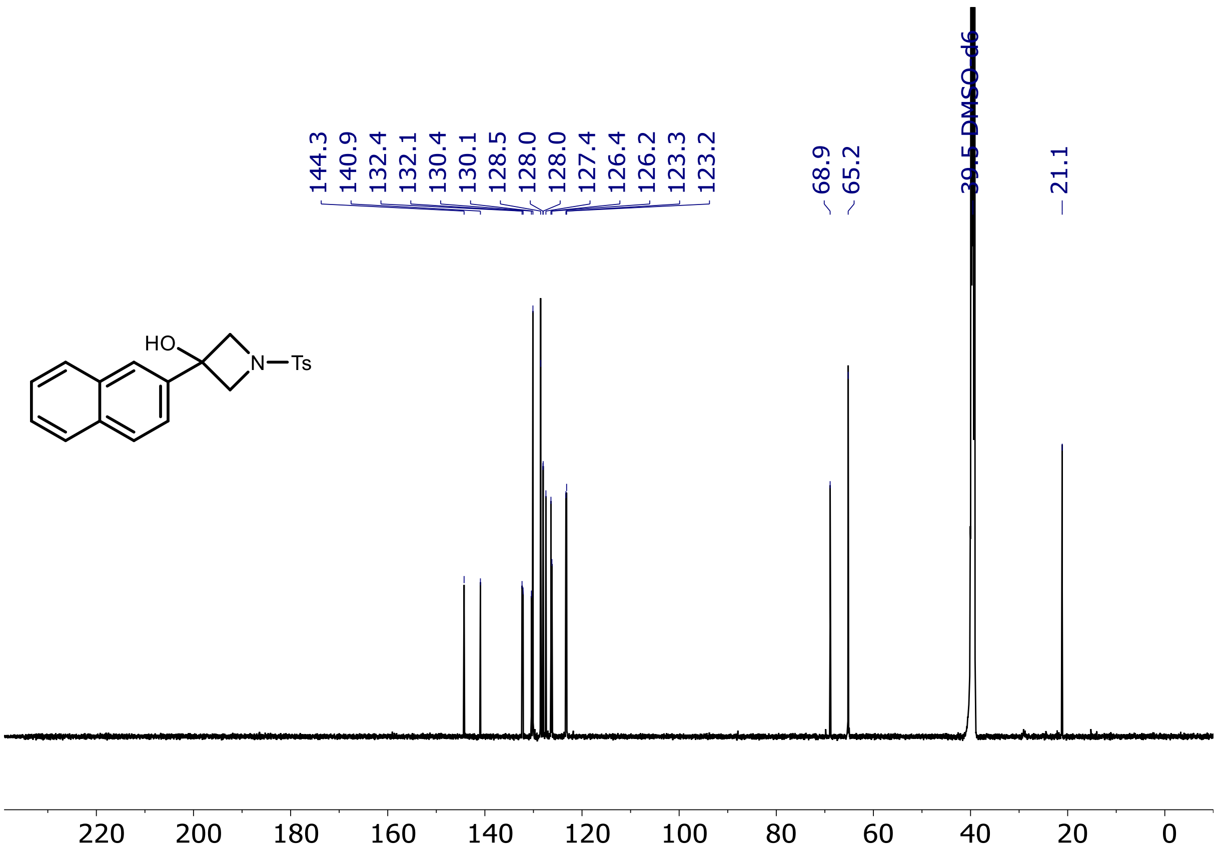

Major-(3c), ^1H, $CDCl_3$, 600 MHz

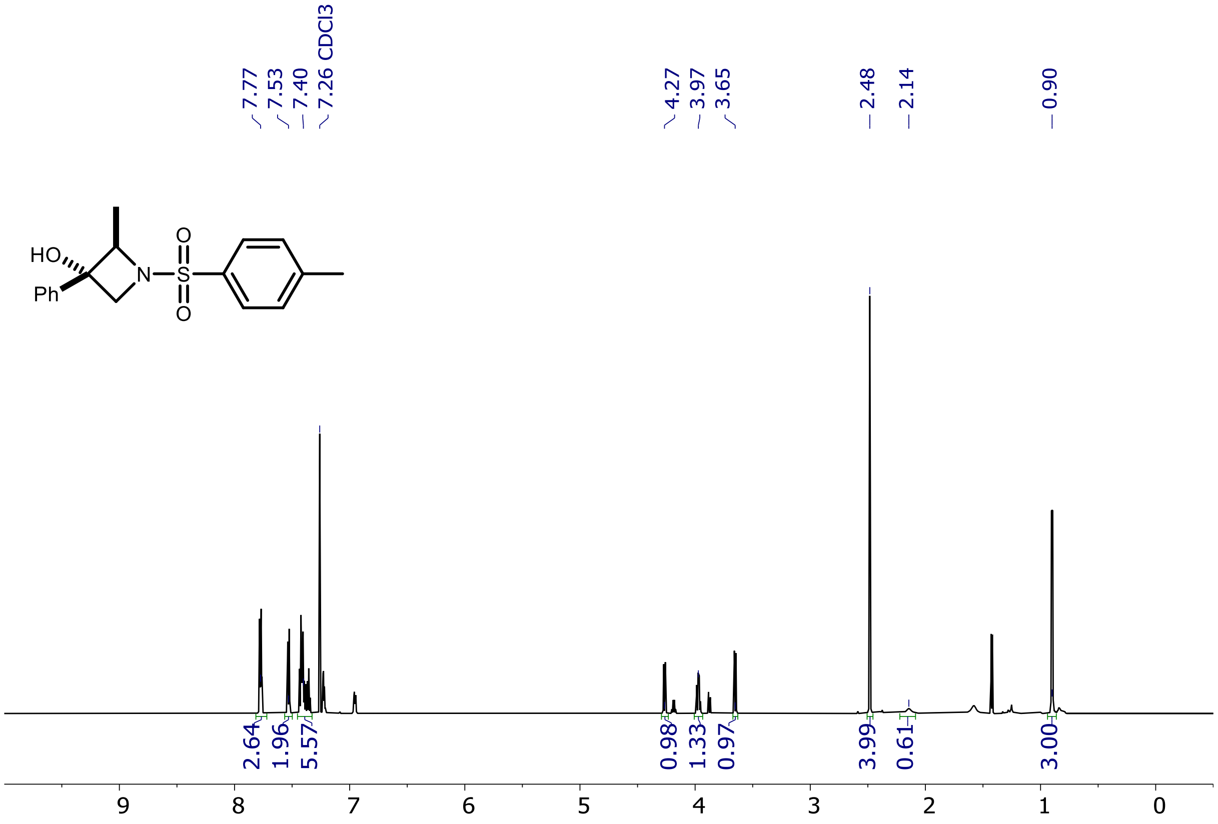

Major-(3c), ^{13}C, $CDCl_3$, 151 MHz

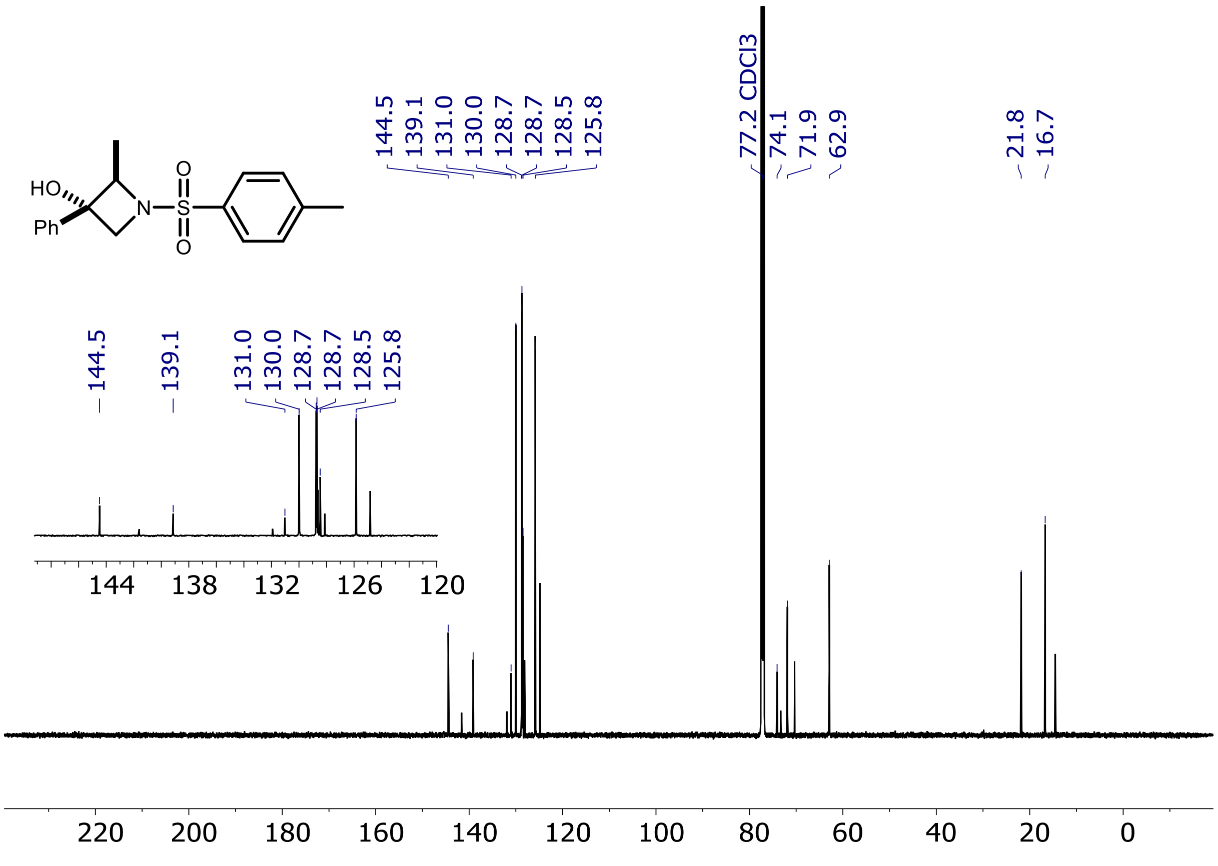

Minor-(3c), ¹H, $CDCl_3$, 600 MHz

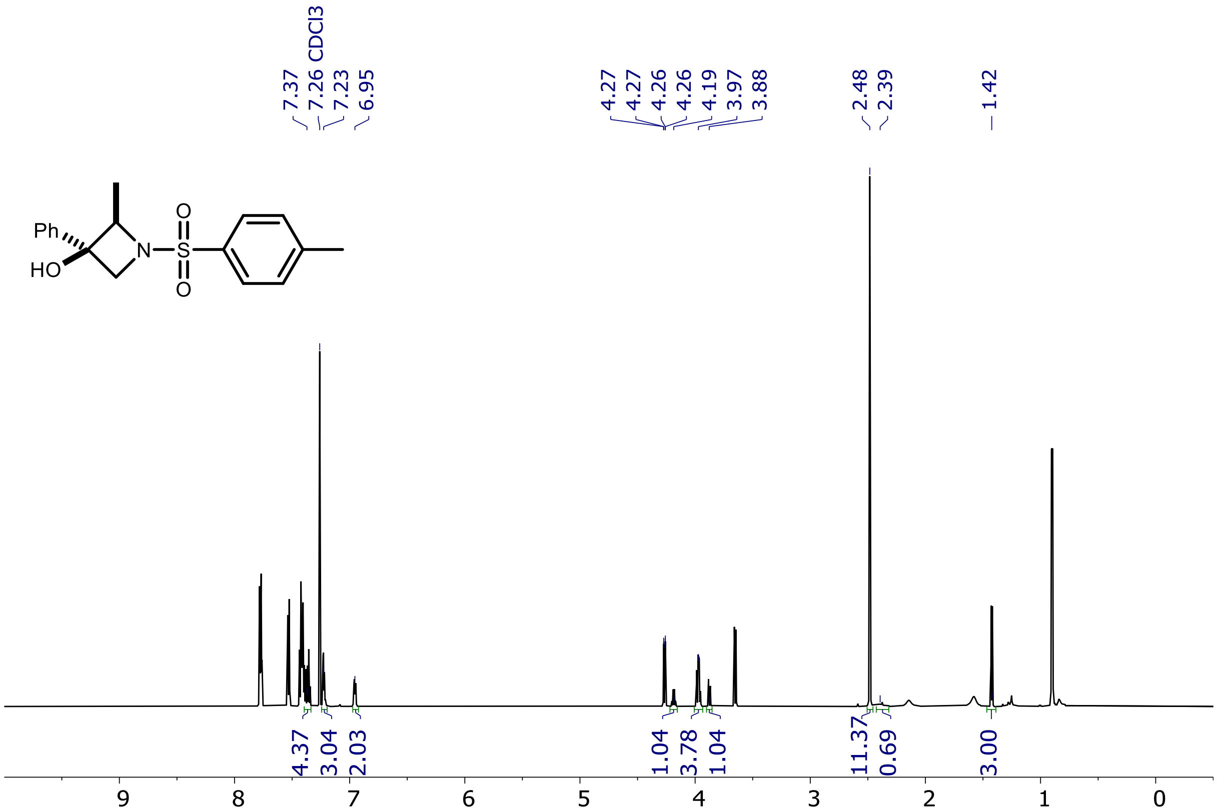

Minor-(3c), ¹³C, $CDCl_3$, 151 MHz

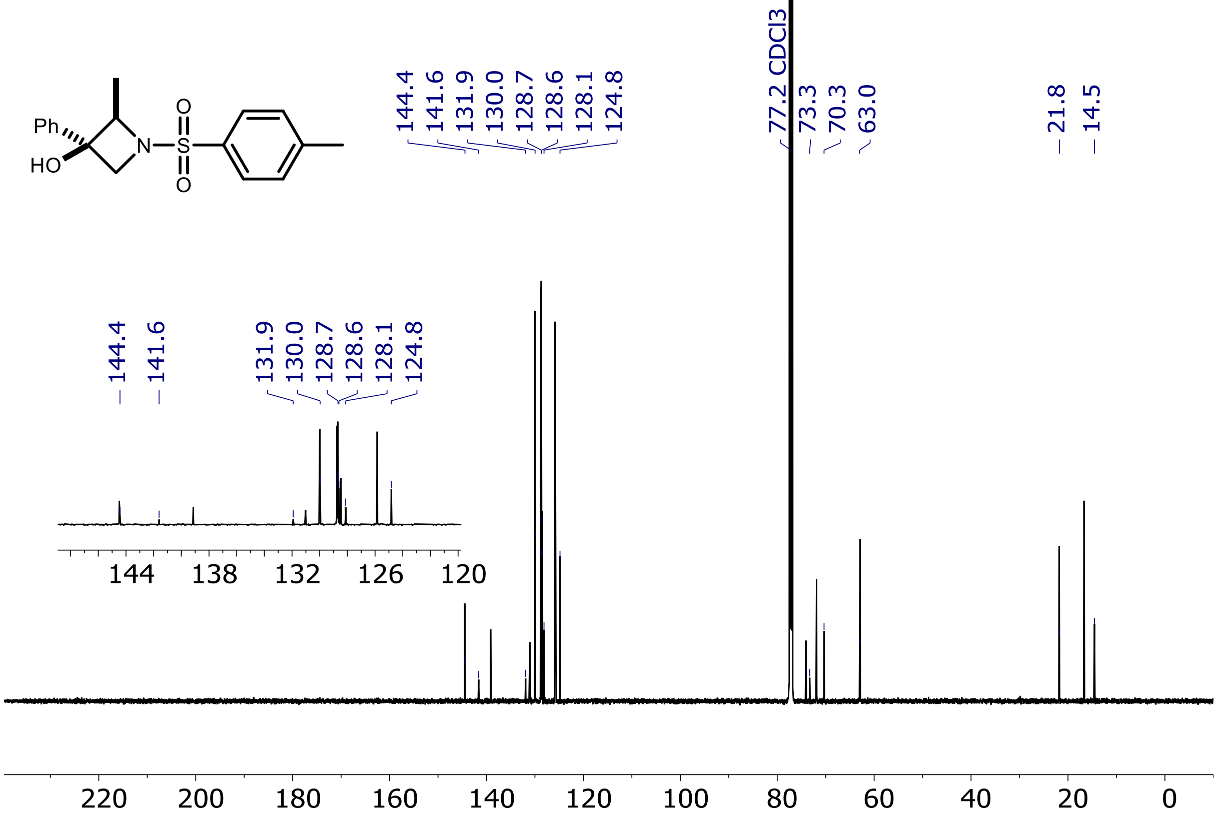

Major-(3d), ^{1}H, DMSO-d_6, 400 MHz

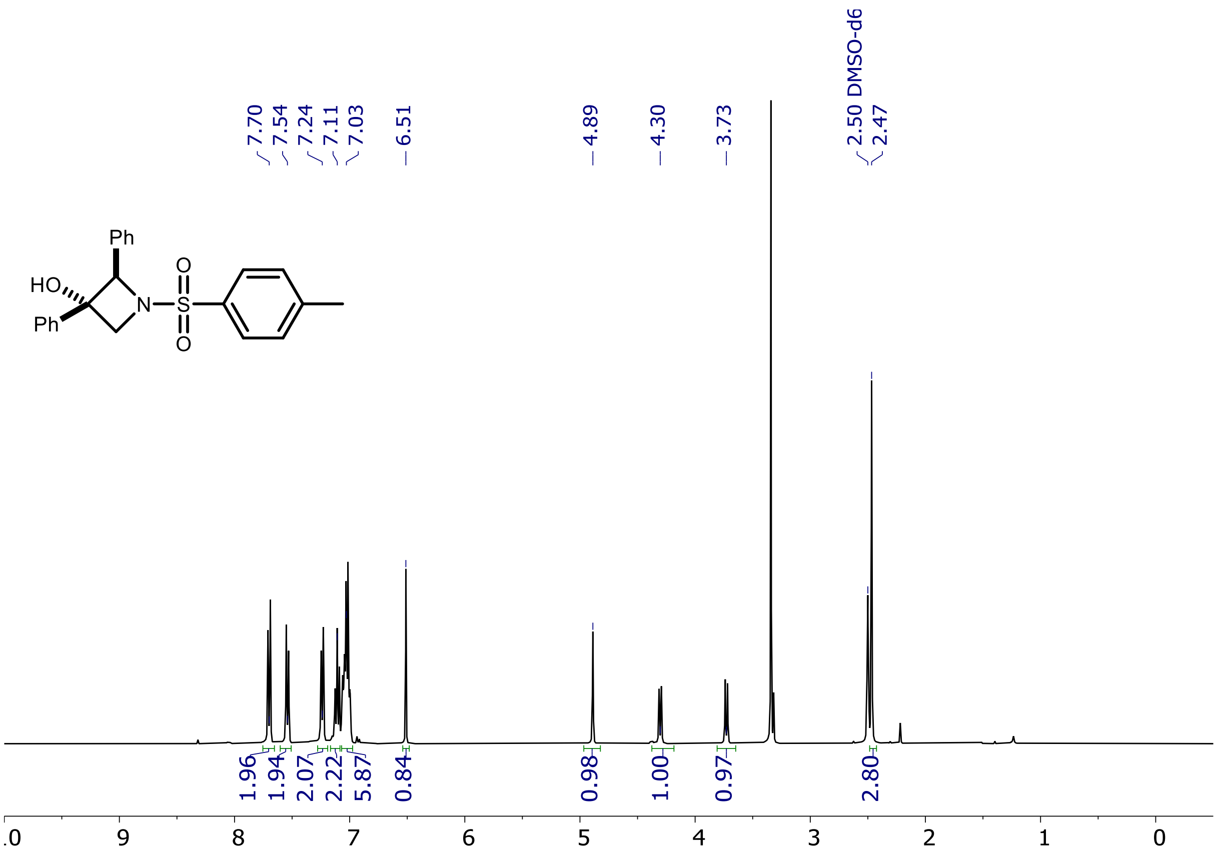

Major-(3d), ^{13}C, DMSO-d_6, 101 MHz

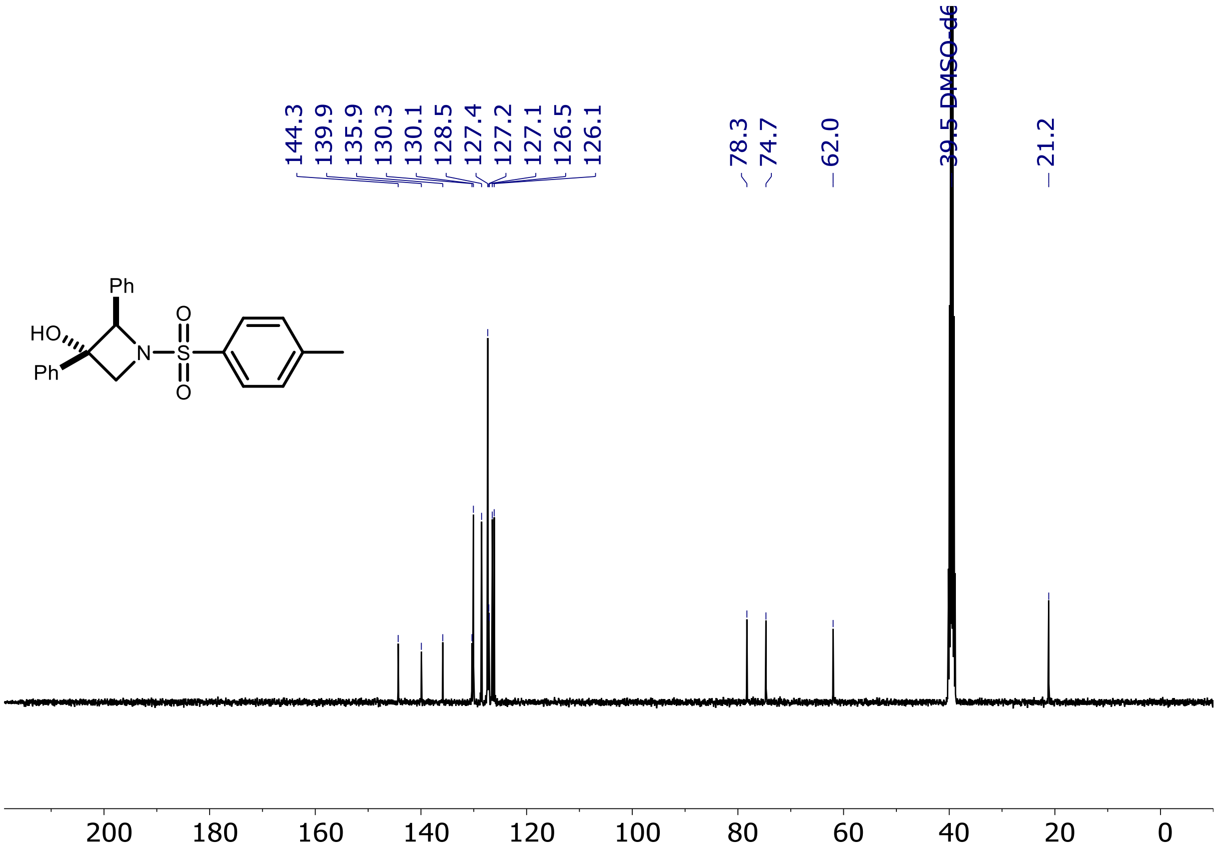

Minor-(3d), ^1H, DMSO-d_6, 400 MHz

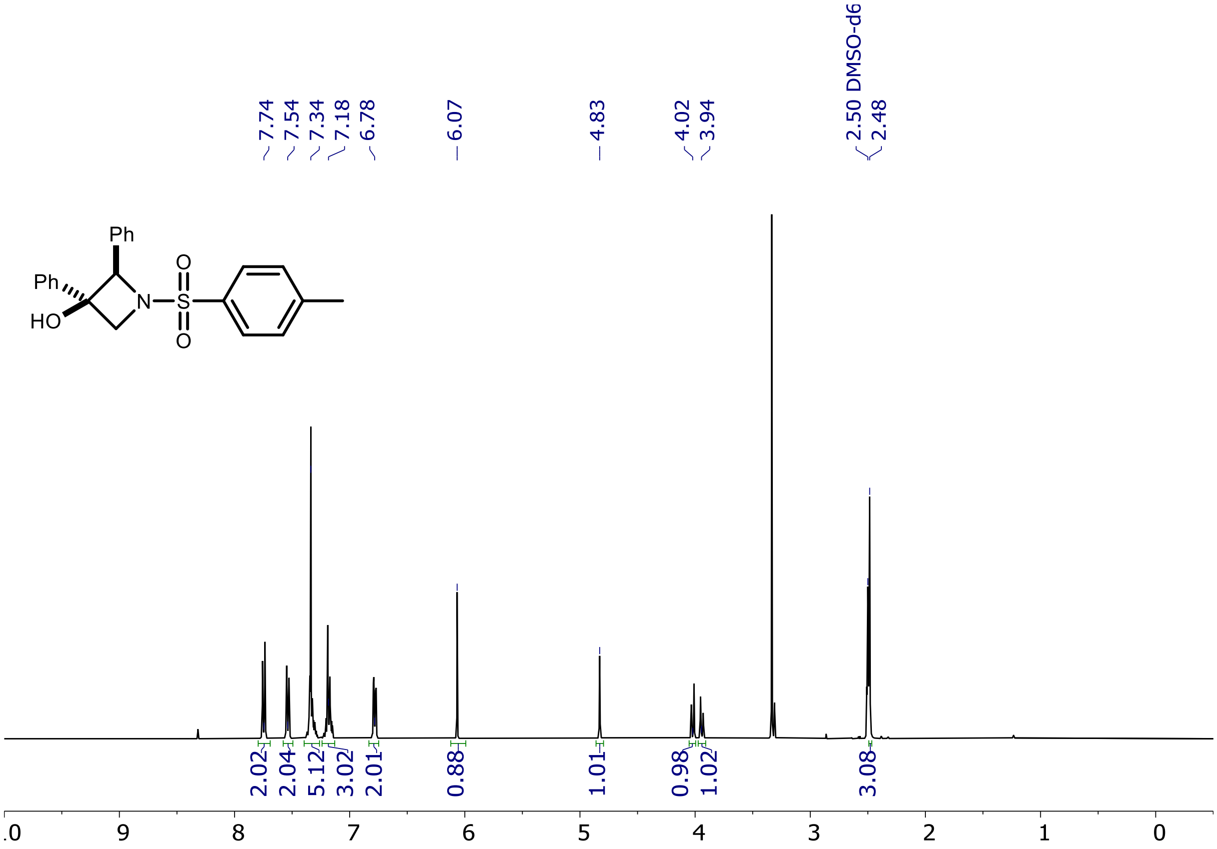

Minor-(3d), ^{13}C, DMSO-d_6, 101 MHz

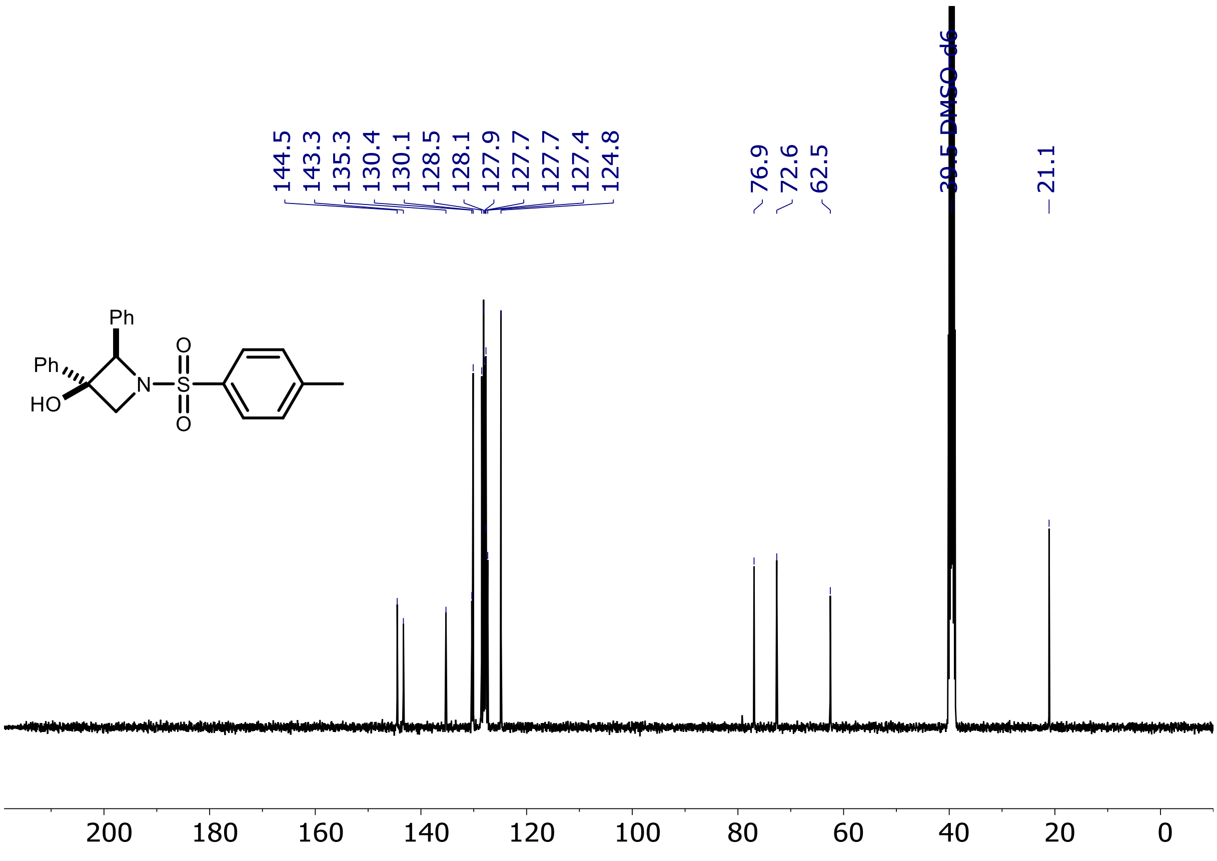

(3e), ^1H, $CDCl_3$, 400 MHz

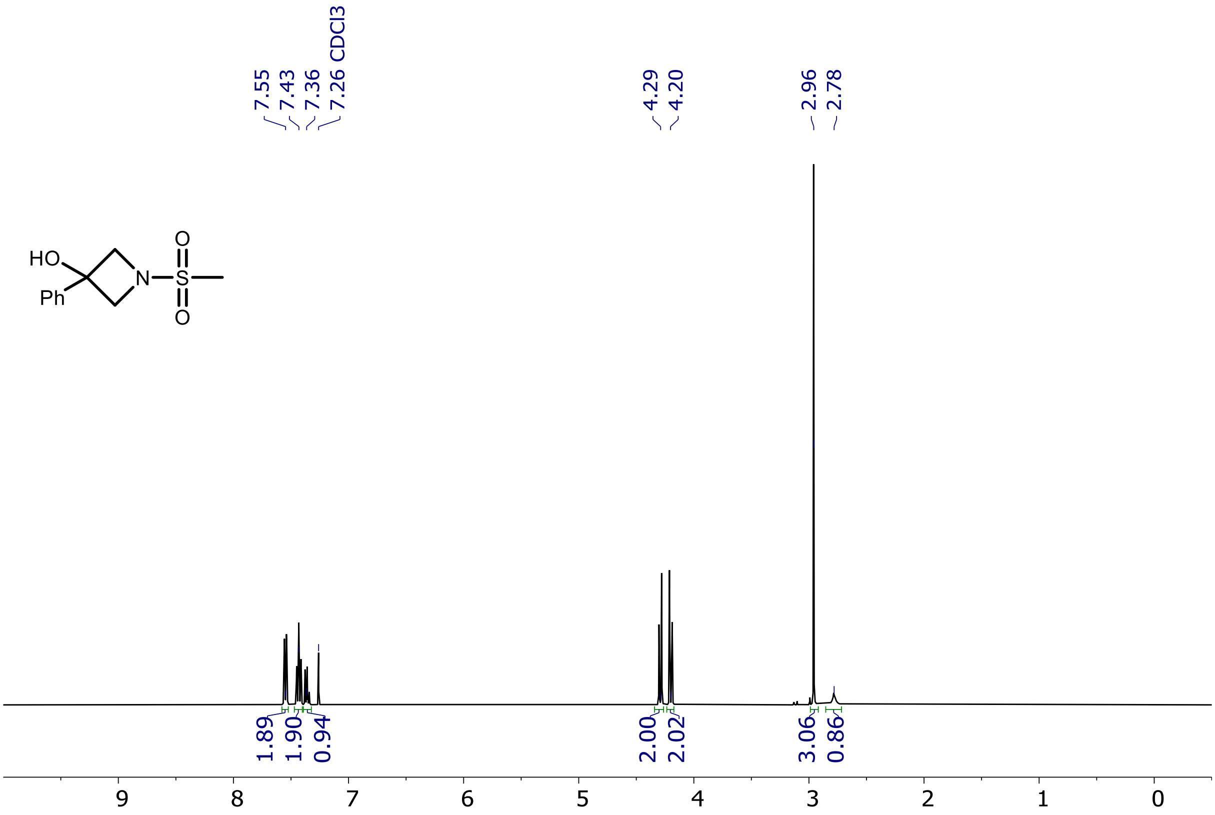

(3e), ^{13}C, $CDCl_3$, 101 MHz

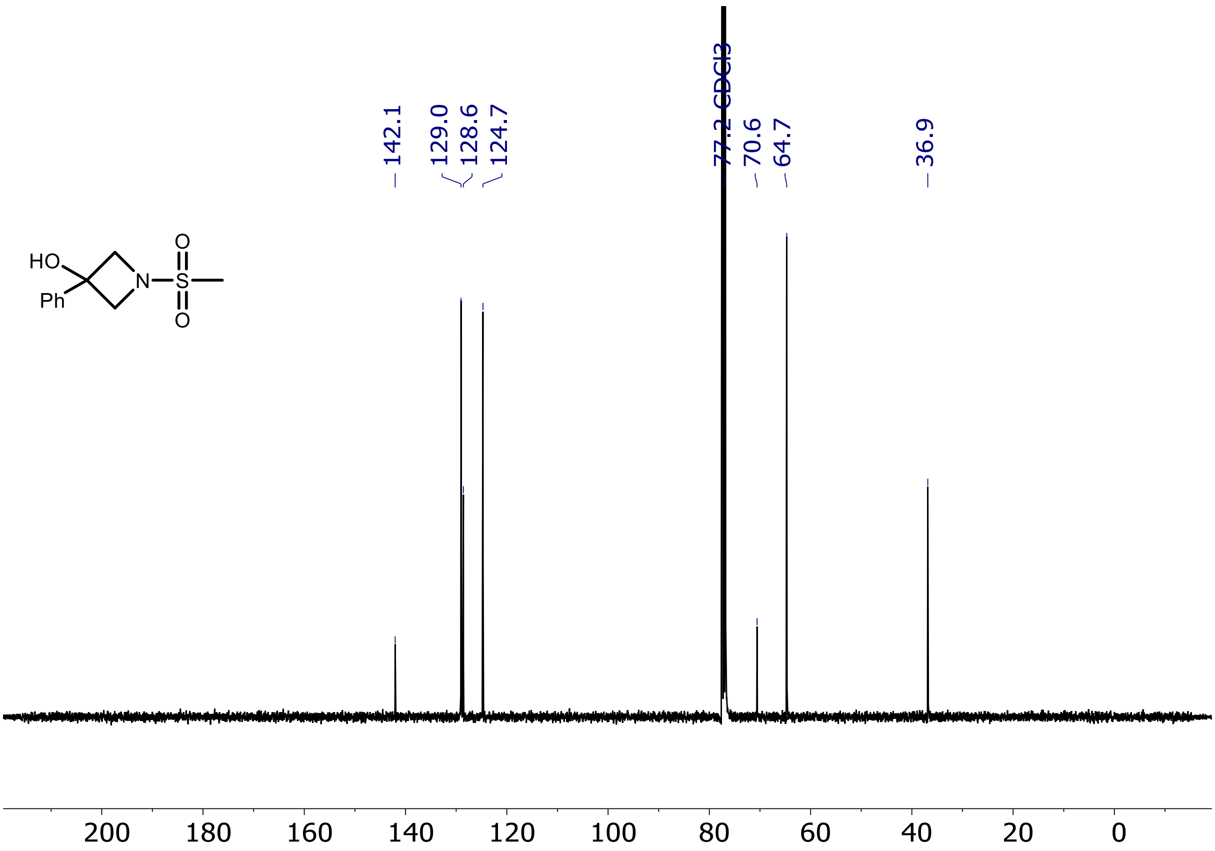

Major-(3f), ^{1}H, CDCl3, 400 MHz

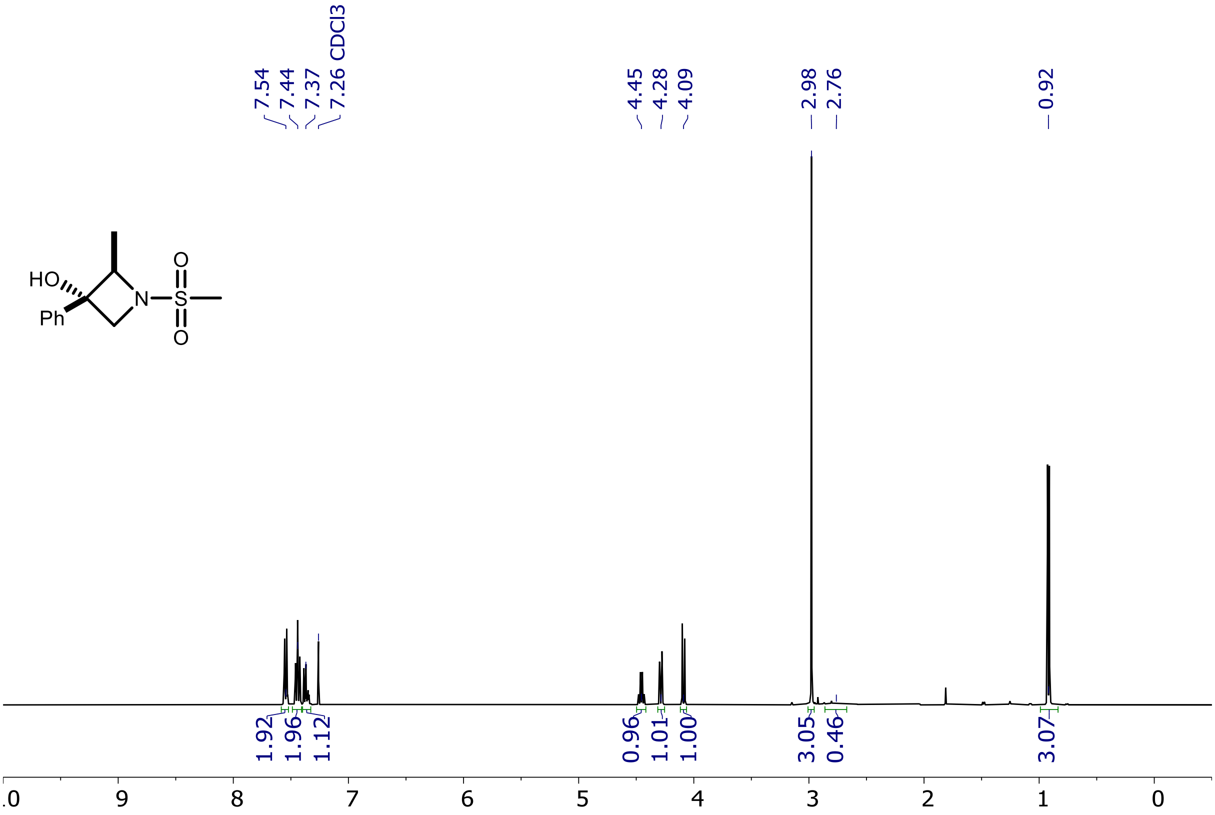

Major-(3f), ^{13}C, CDCl3, 101 MHz

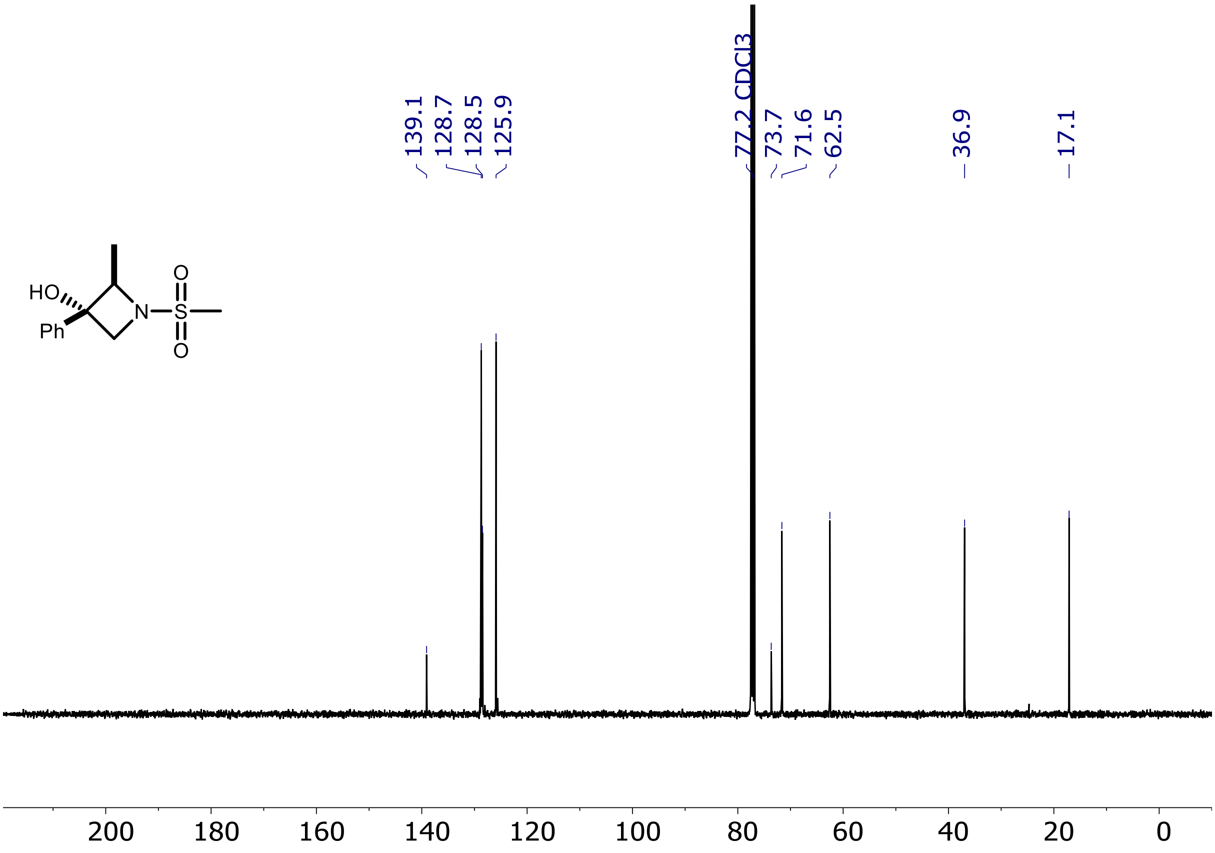

Minor-(3f), ^{1}H, CDCl3, 400 MHz

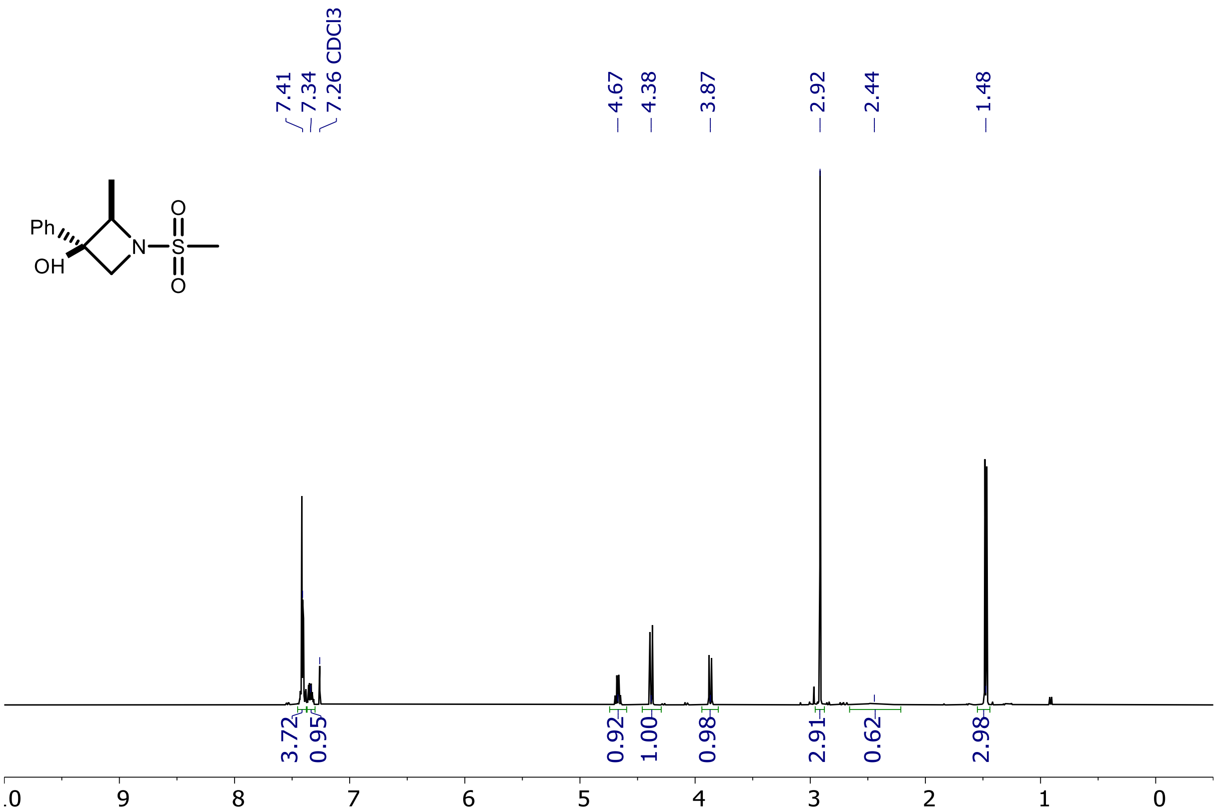

Minor-(3f), ^{13}C, CDCl3, 101 MHz

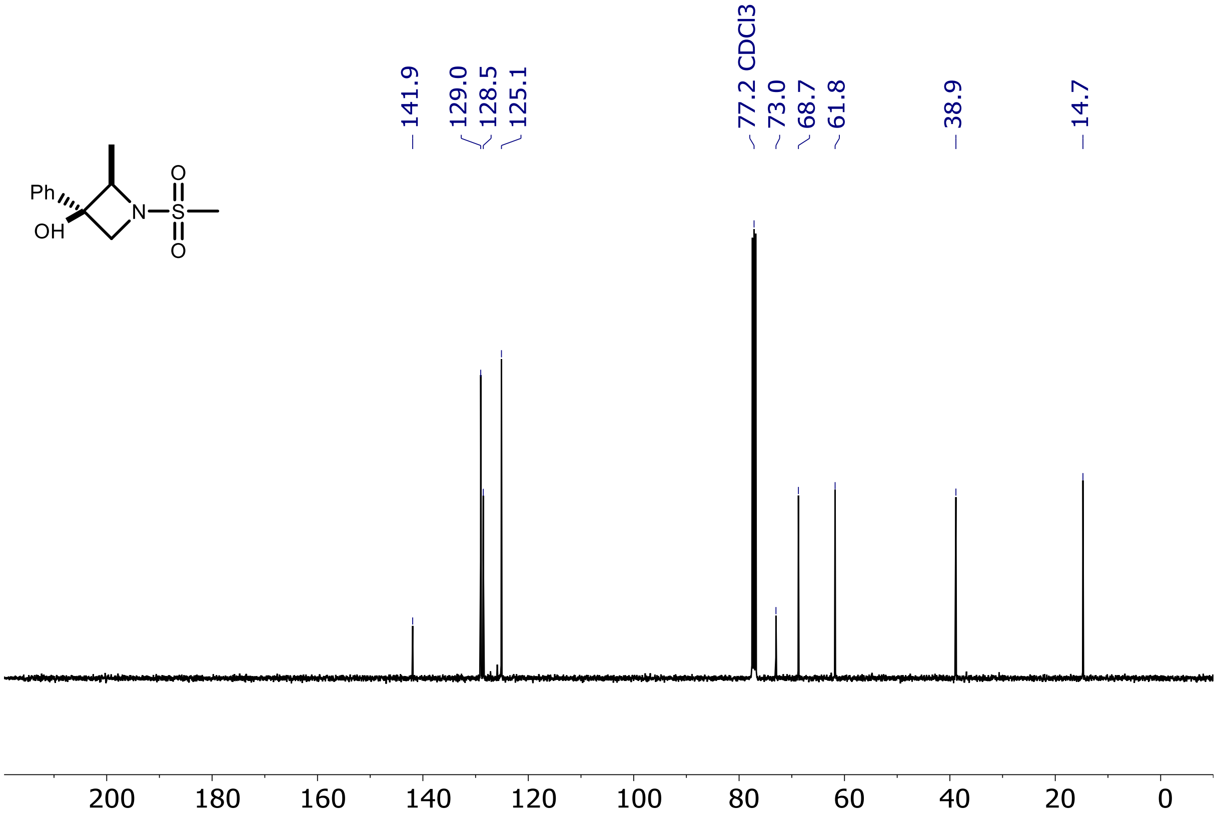

Major-(3g), ^{1}H, $CDCl_3$, 400 MHz

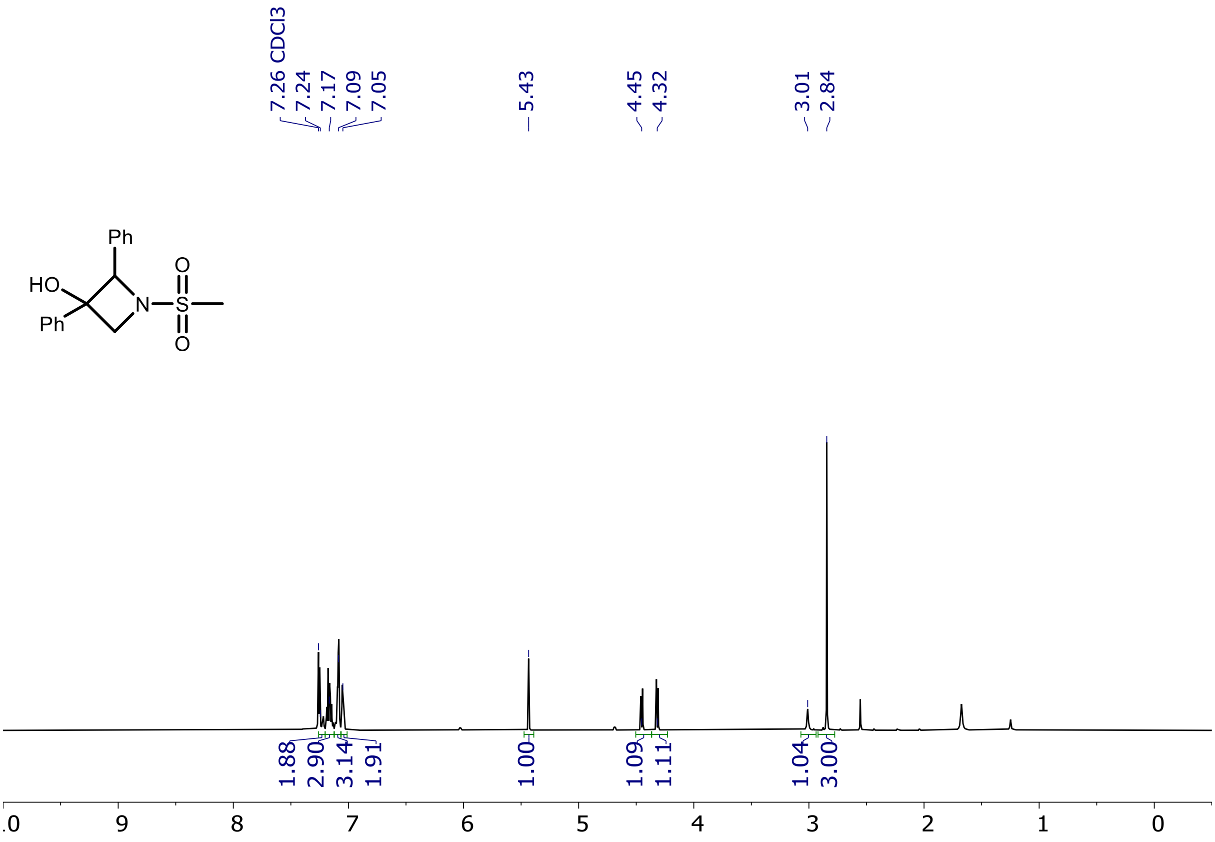

Major-(3g), ^{13}C, $CDCl_3$, 101 MHz

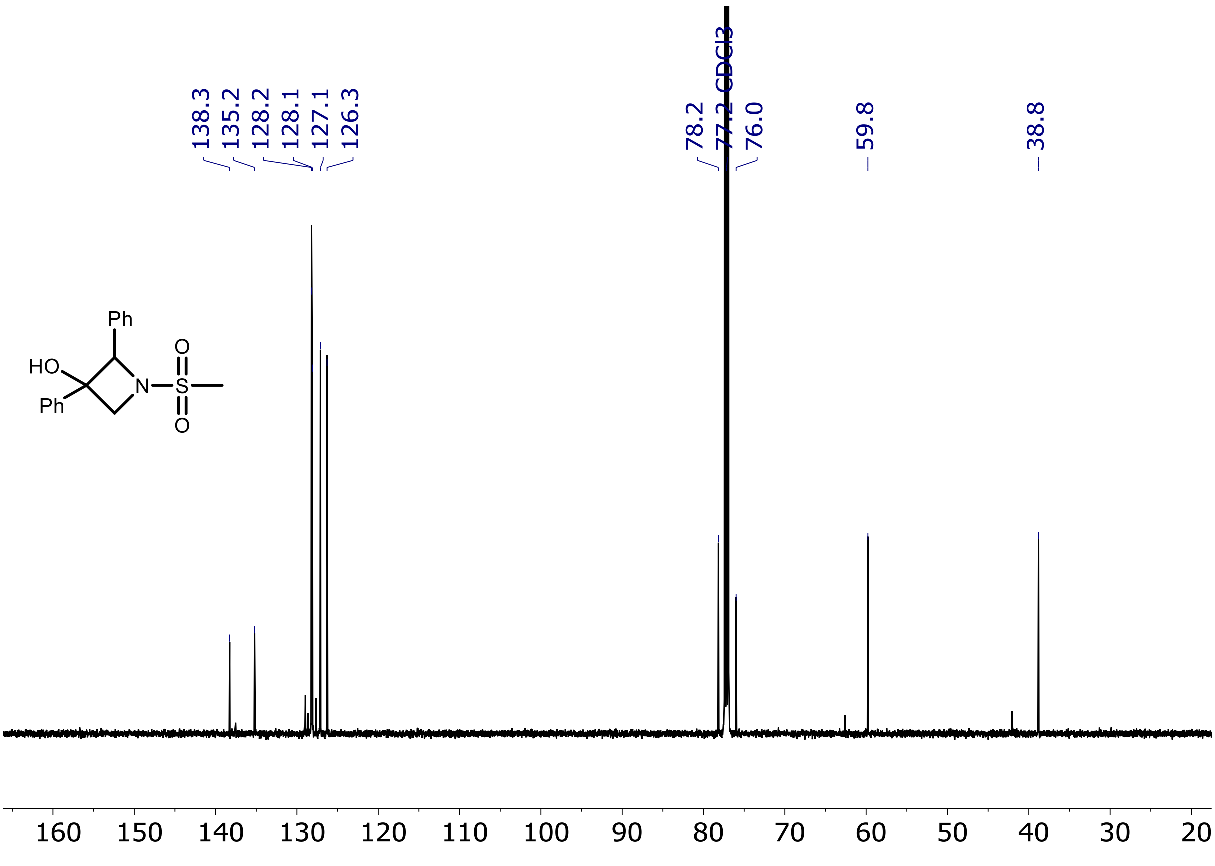

Minor-(3g), ¹H, CDCl₃, 400 MHz

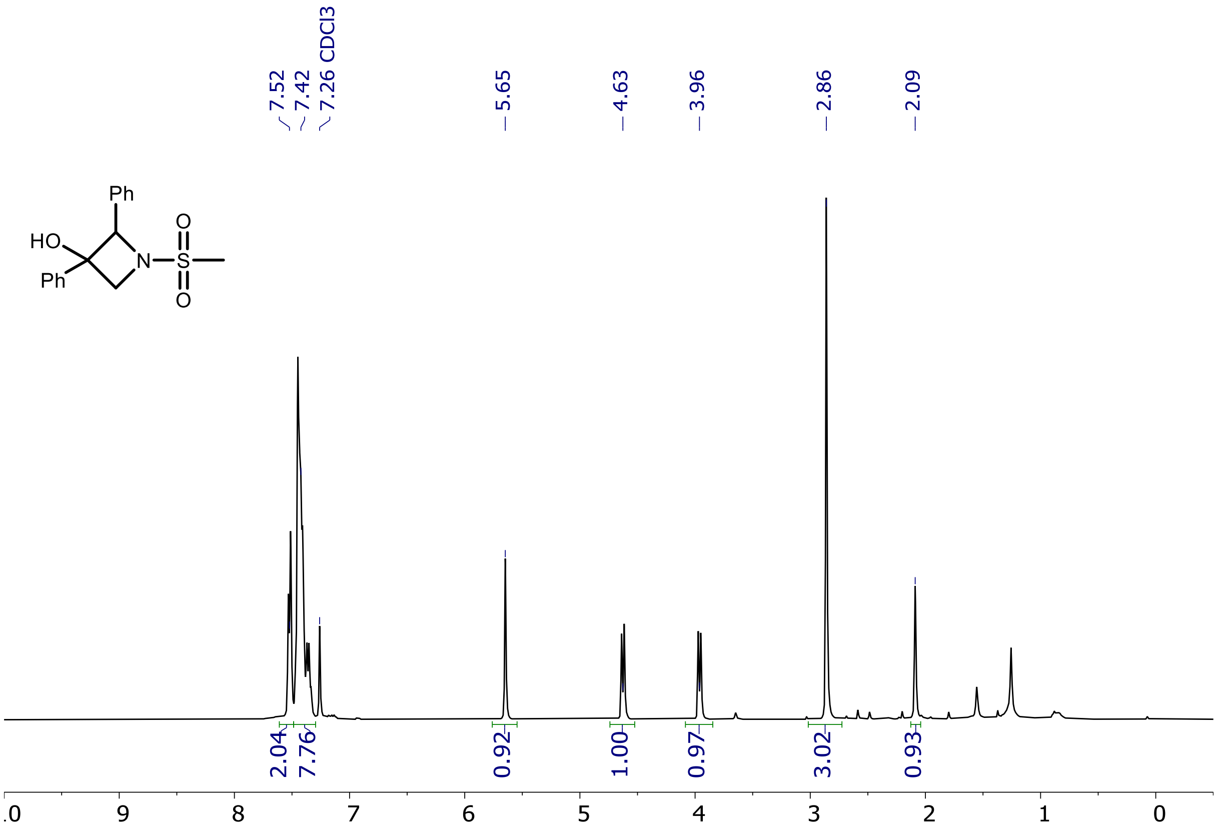

Minor-(3g), ¹³C, CDCl₃, 101 MHz

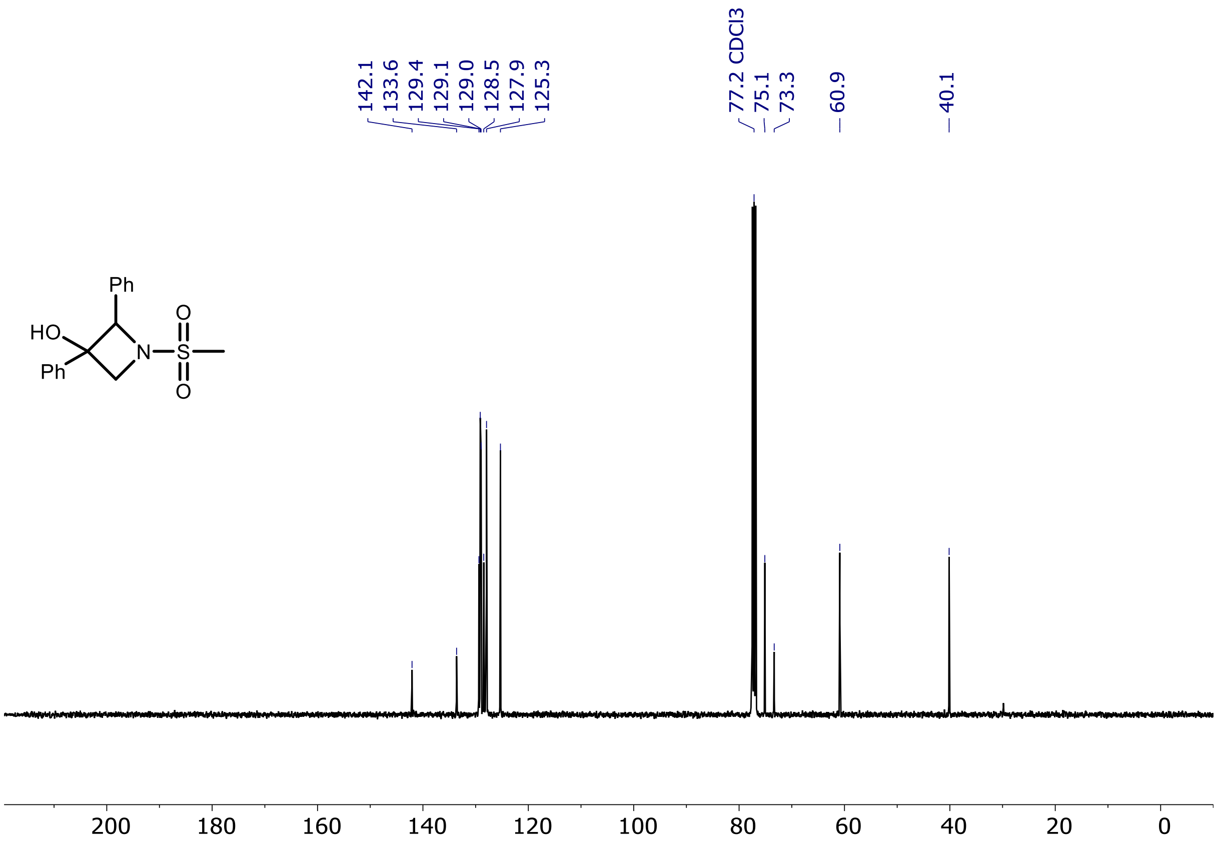

(3h), ^{1}H, CDCl$_3$, 400 MHz

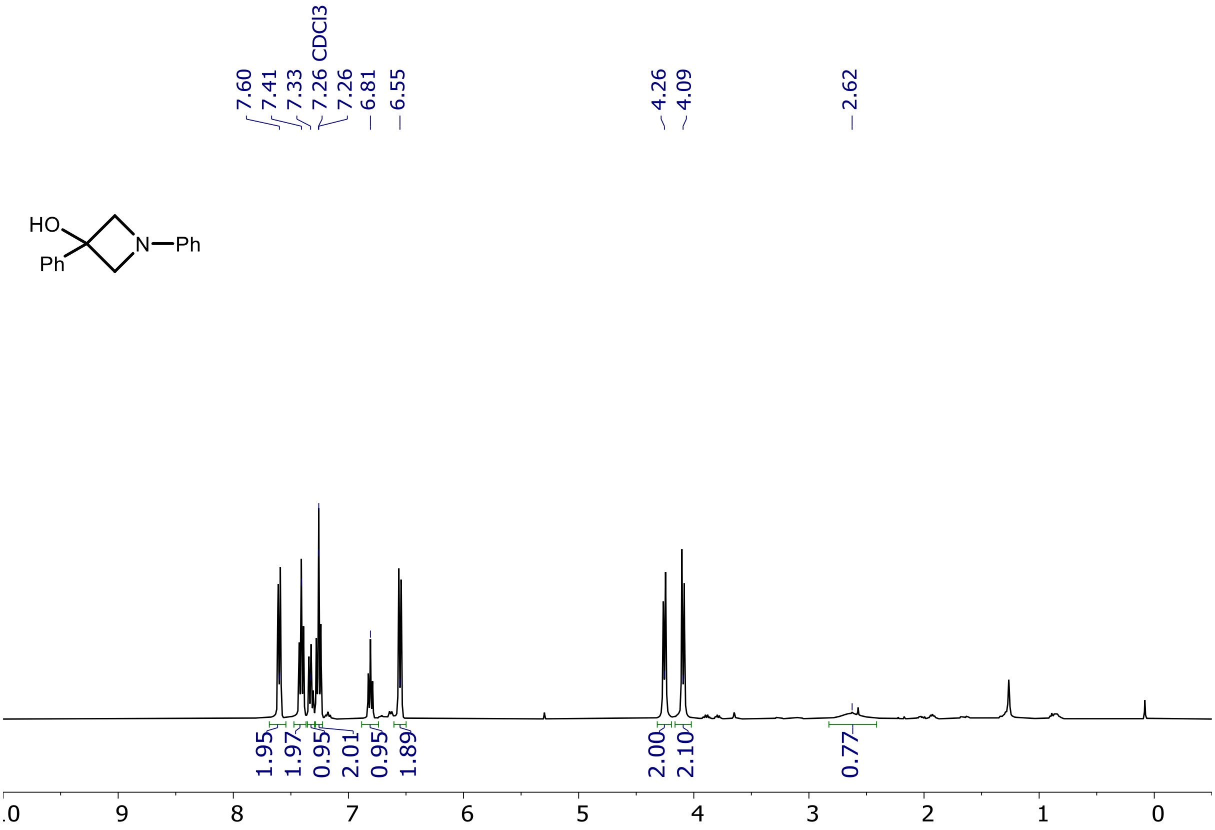

(3h), ^{13}C, CDCl$_3$, 101 MHz

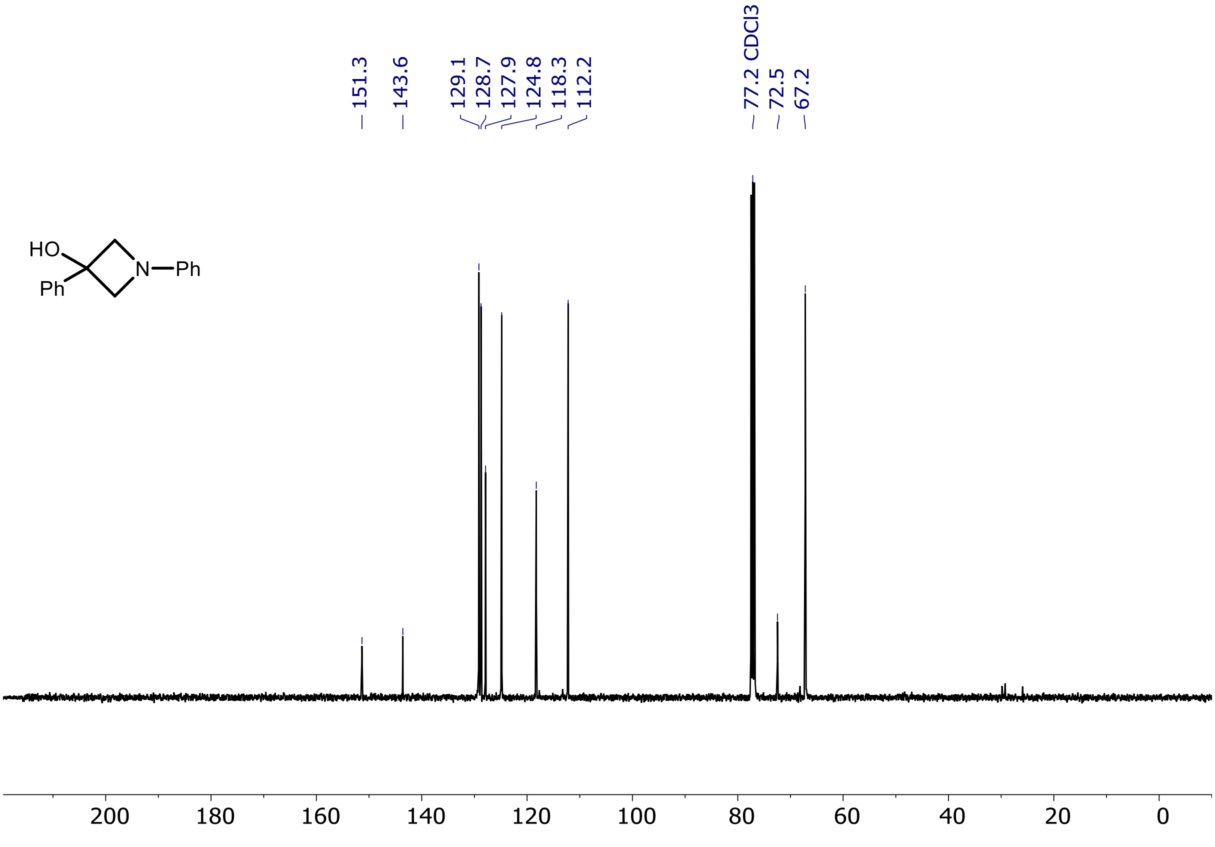

Major-(3i), ¹H, $CDCl_3$, 400 MHz

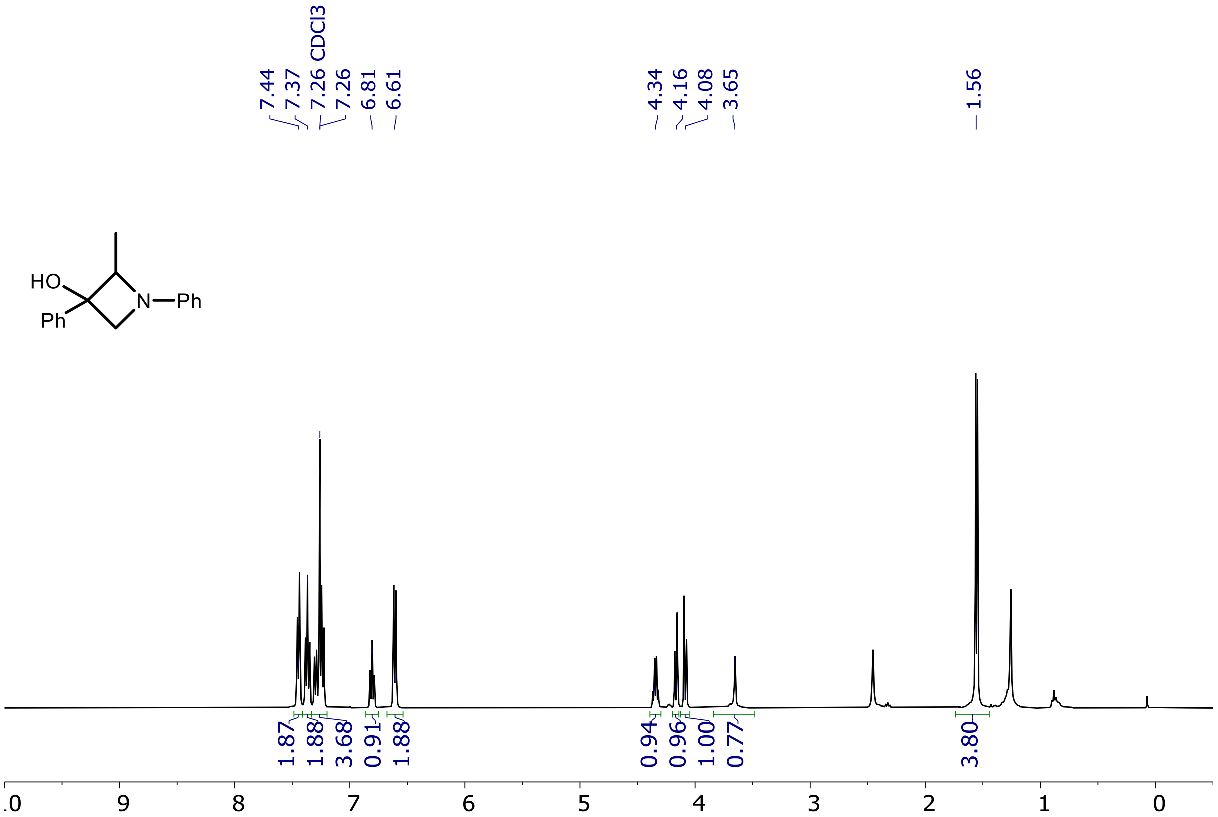

Major-(3i), ¹³C, $CDCl_3$, 101 MHz

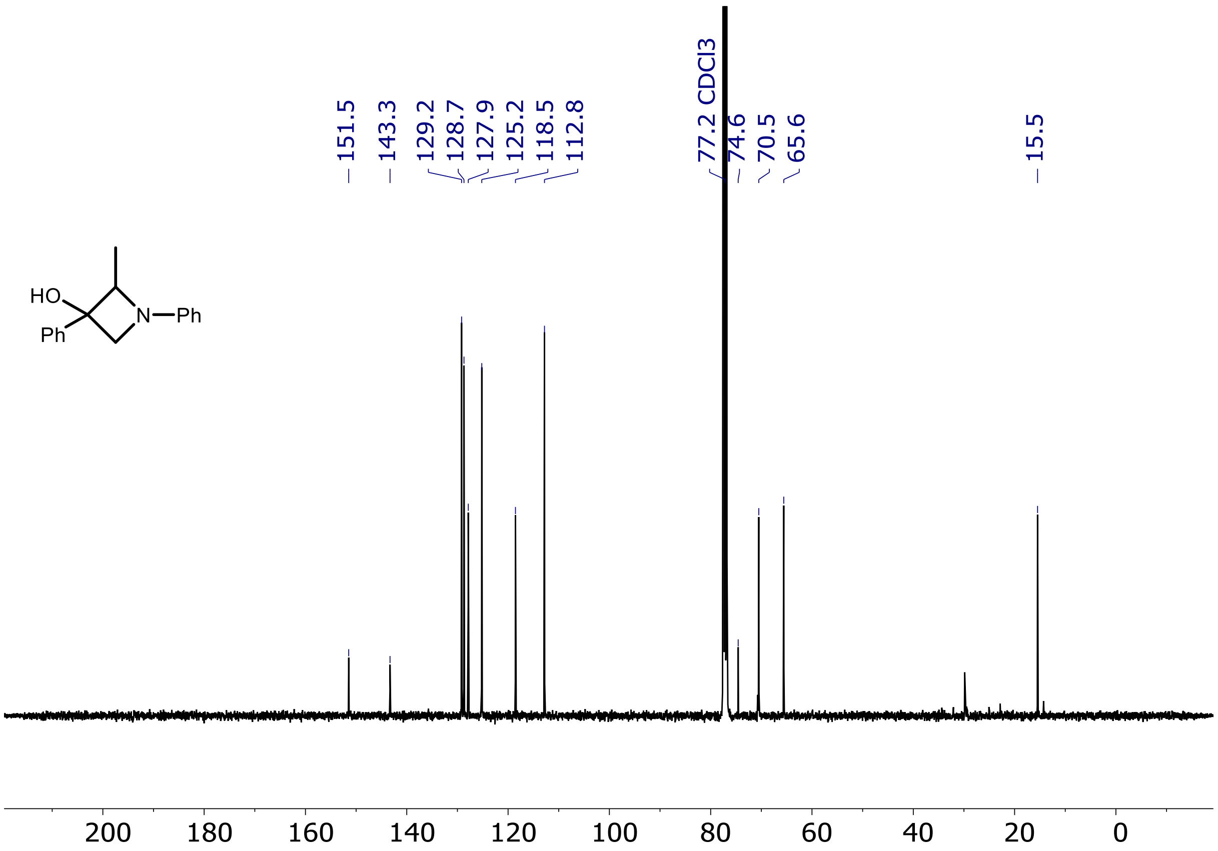

Minor-(3i), ^{1}H, $CDCl_3$, 400 MHz

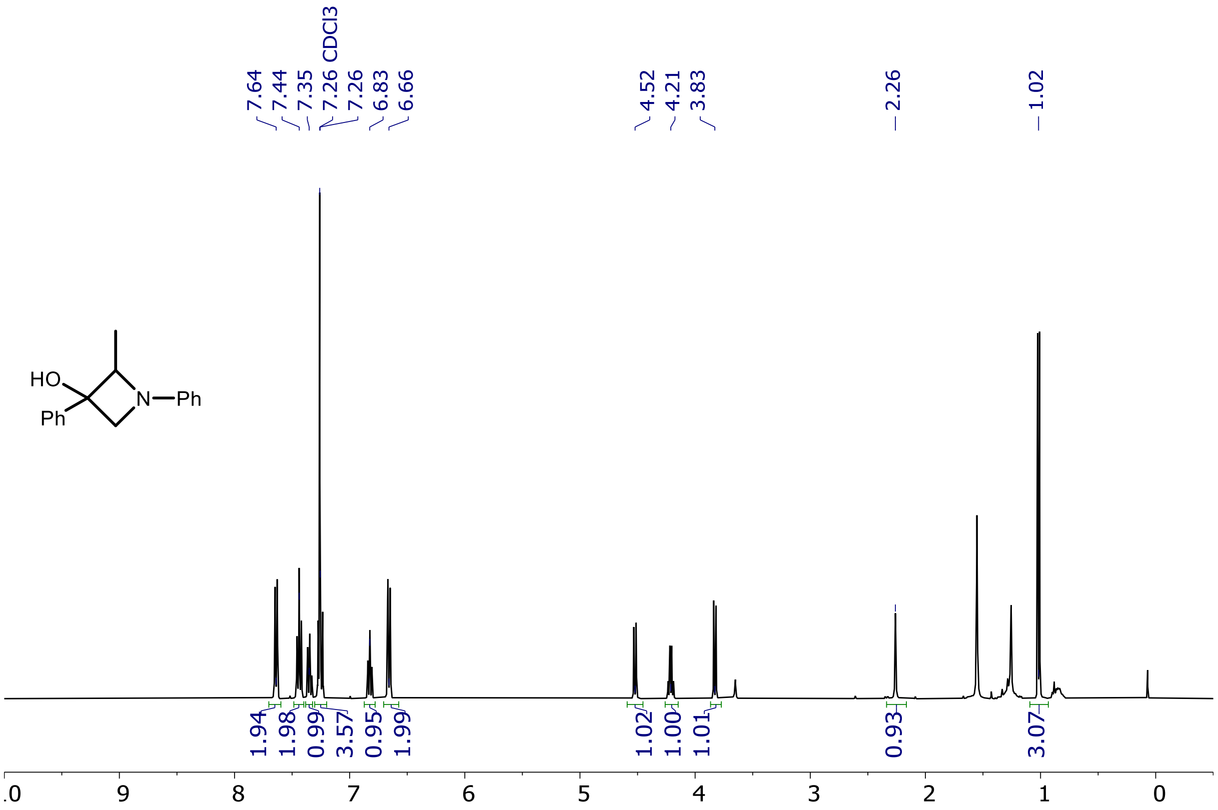

Minor-(3i), ^{13}C, $CDCl_3$, 101 MHz

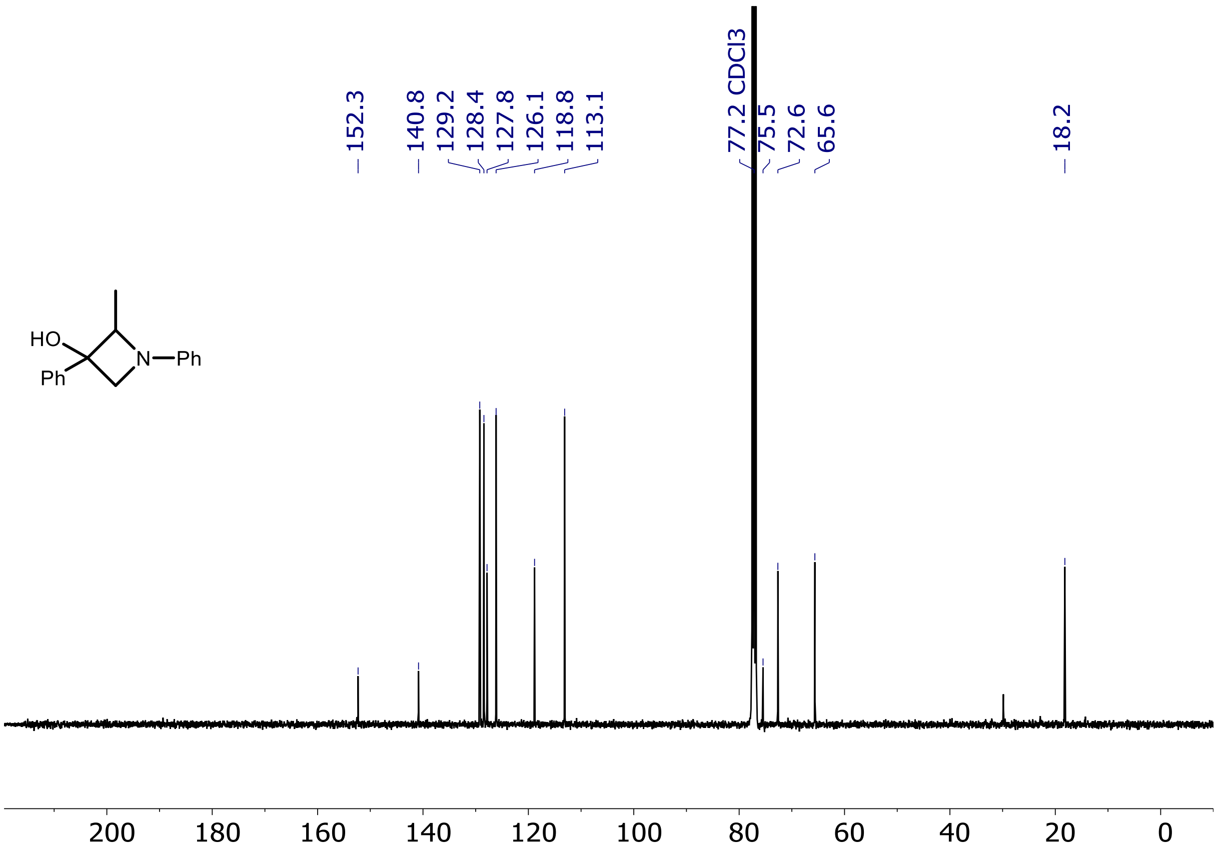

(3l), ^{1}H, $CDCl_3$, 400 MHz

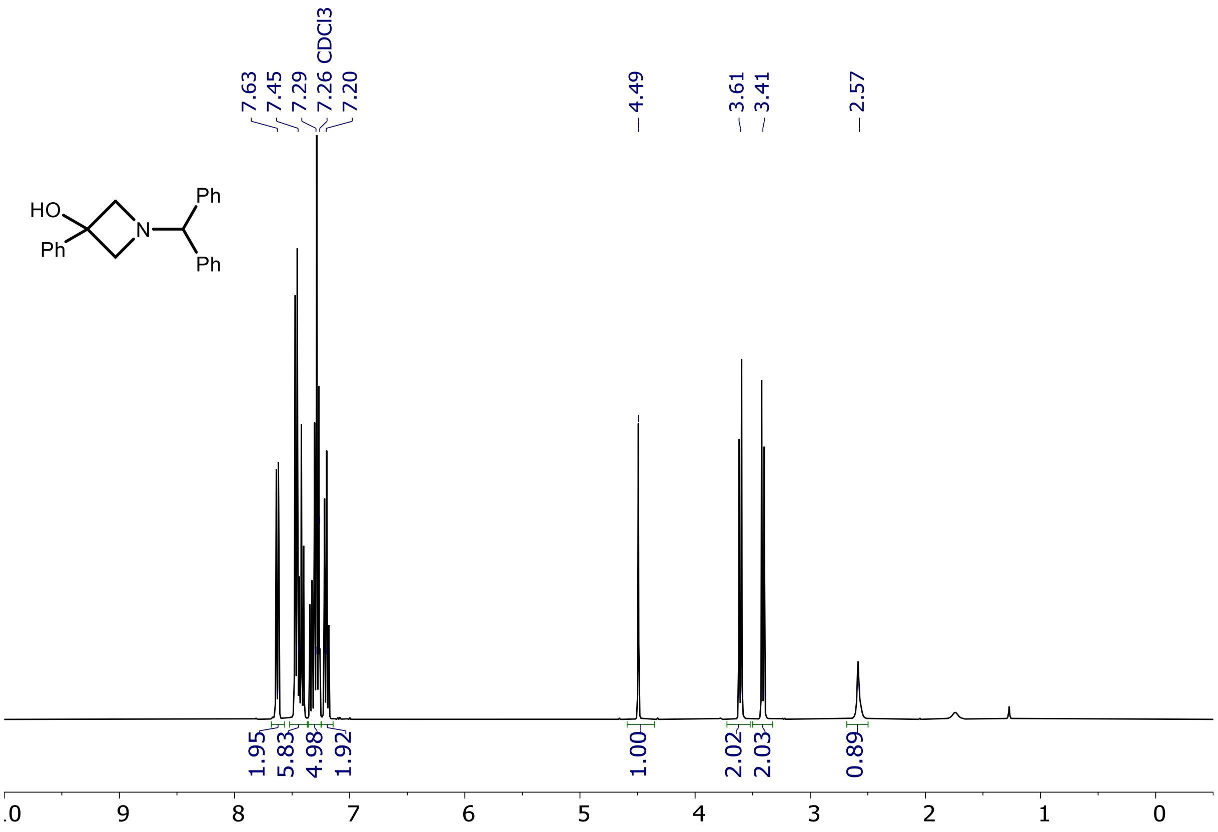

(3l), ^{13}C, $CDCl_3$, 101 MHz

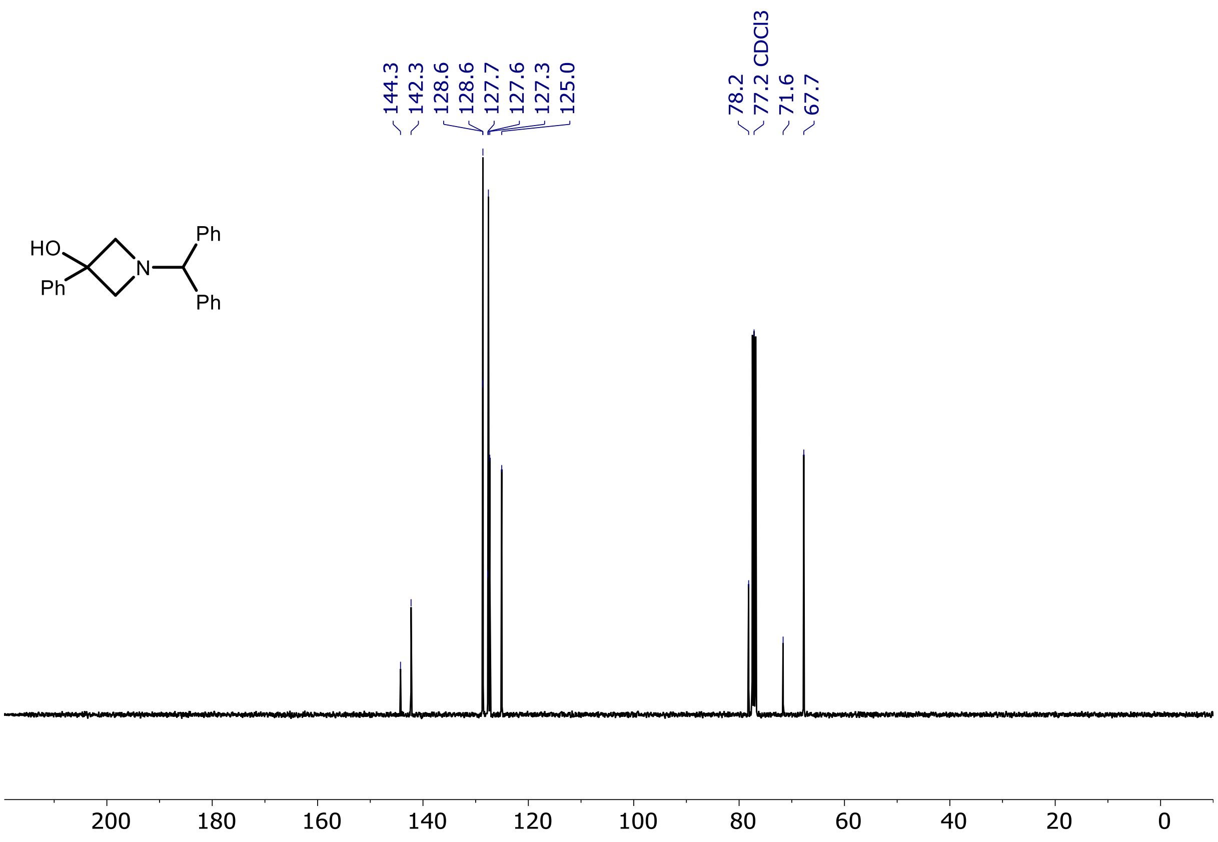

(11), ^{1}H, C_6D_6, 600 MHz

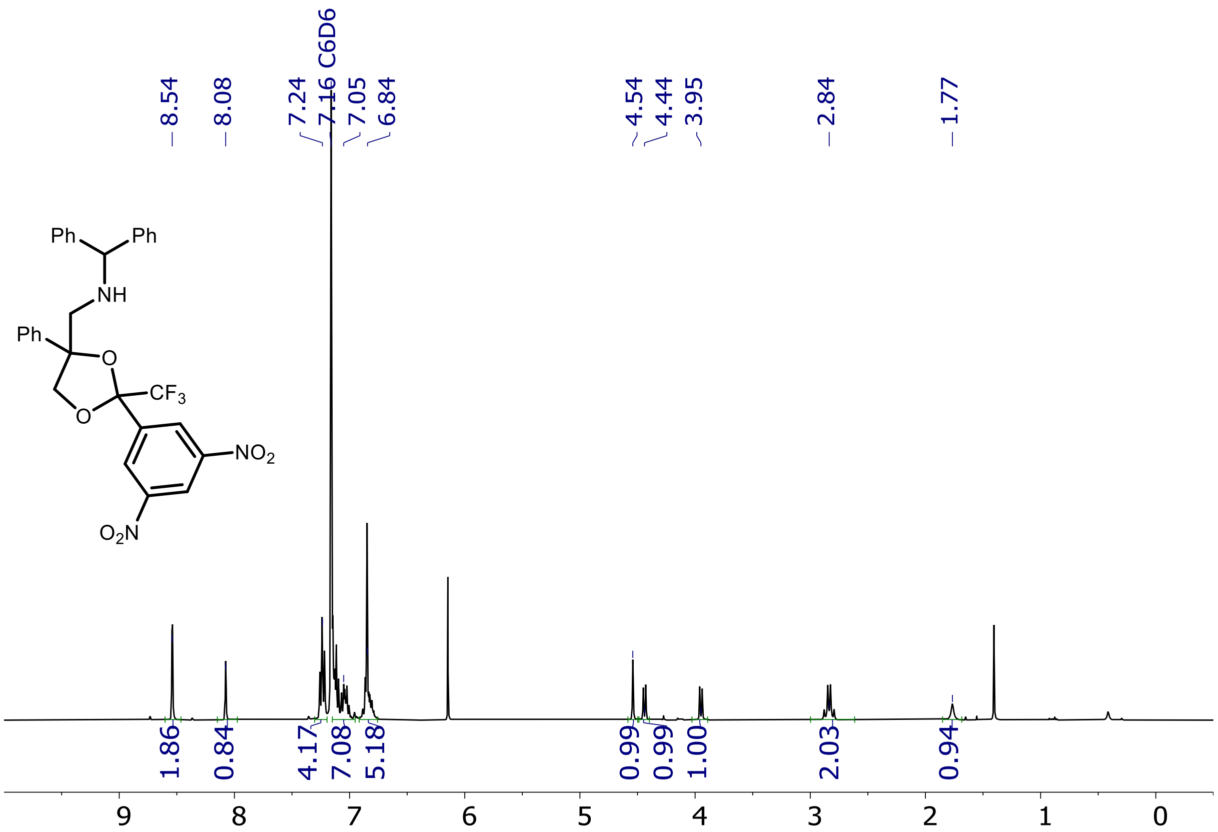

(11), ^{13}C, C_6D_6, 161 MHz

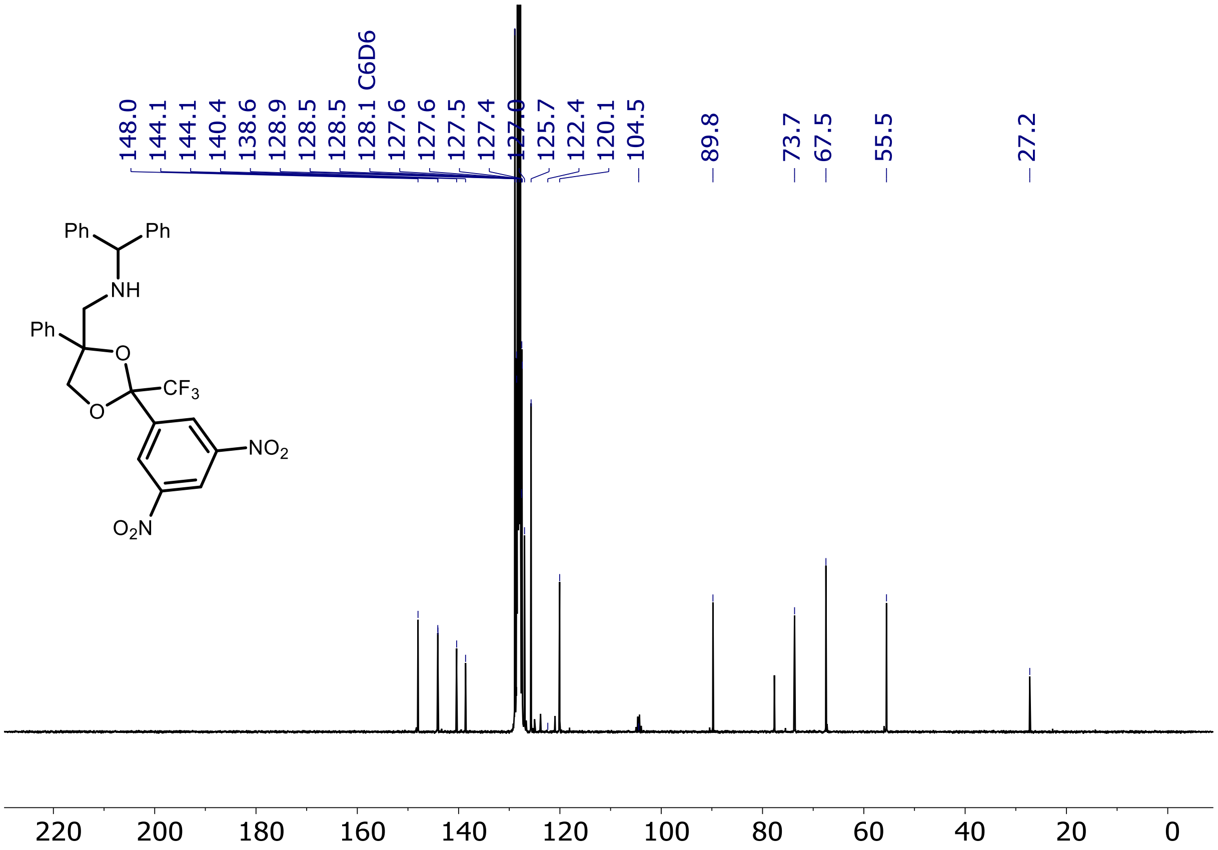

(11), ^{19}F, C_6D_6, 282 MHz

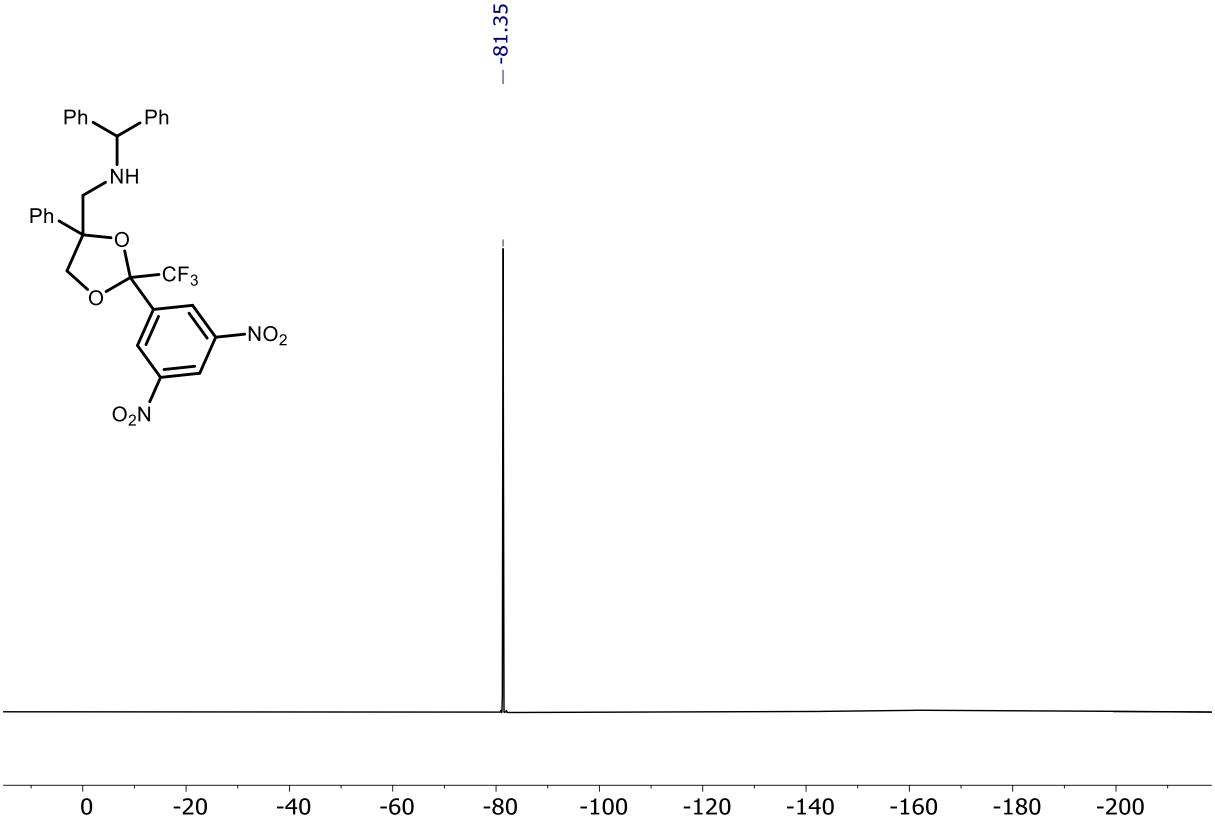

(16), ^{1}H, C_6D_6, 600 MHz

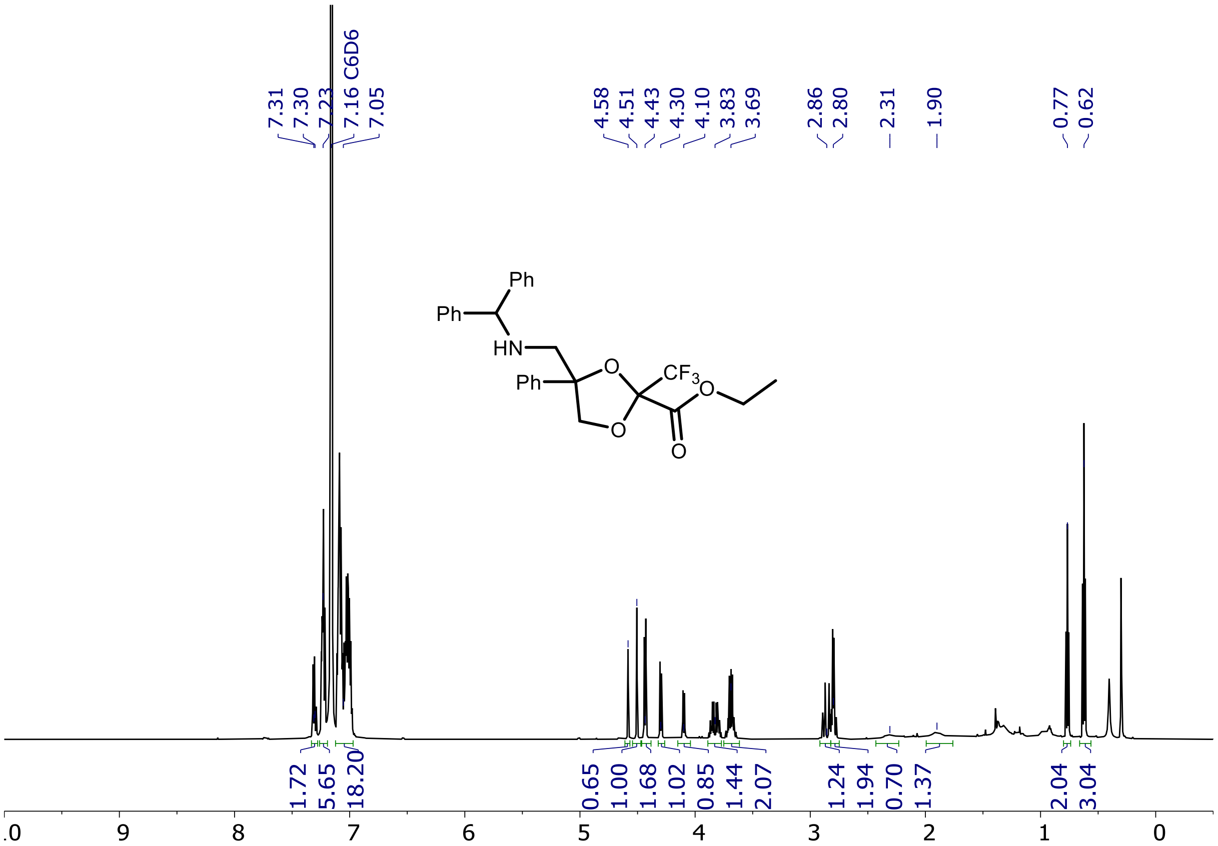

(16), ^{13}C, C_6D_6, 151 MHz

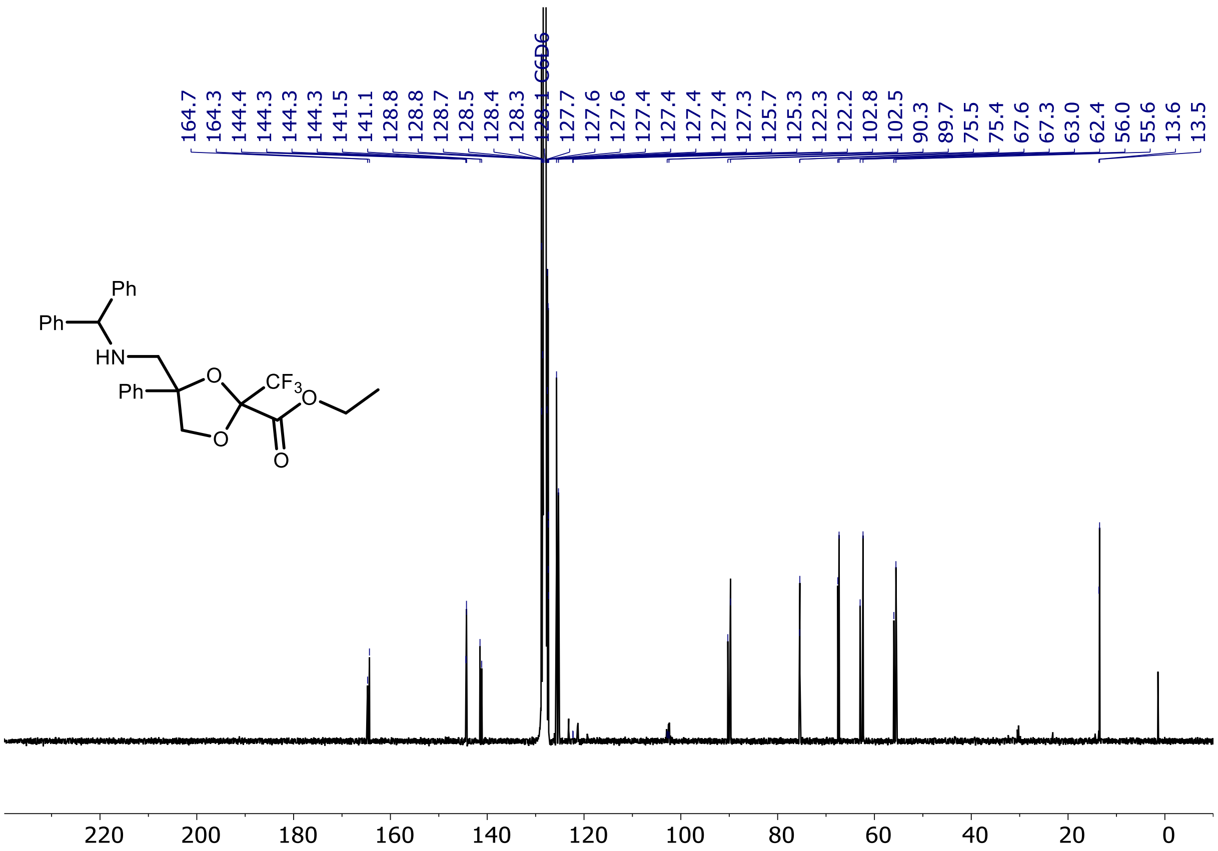

(16), ^{19}F, C_6D_6, 282 MHz

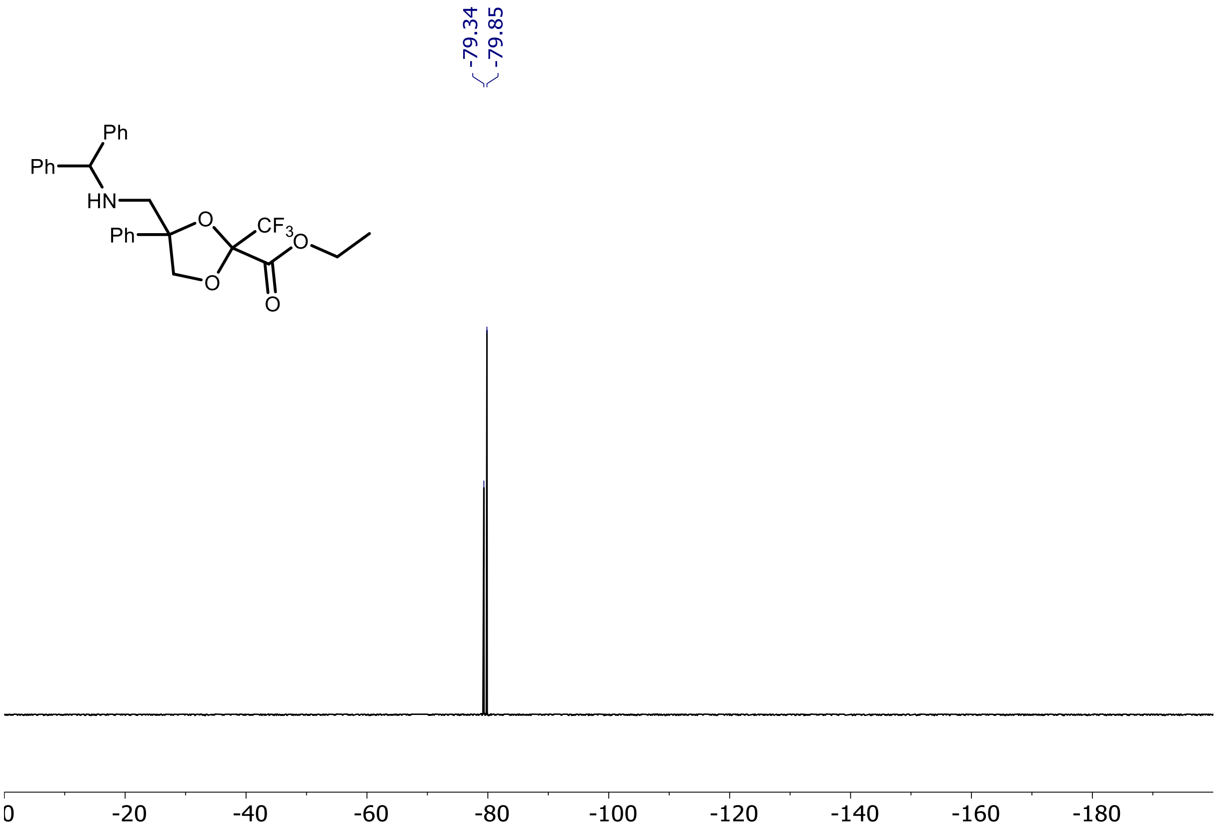

(16), ^{1}H ^{1}H COSY, C_6D_6

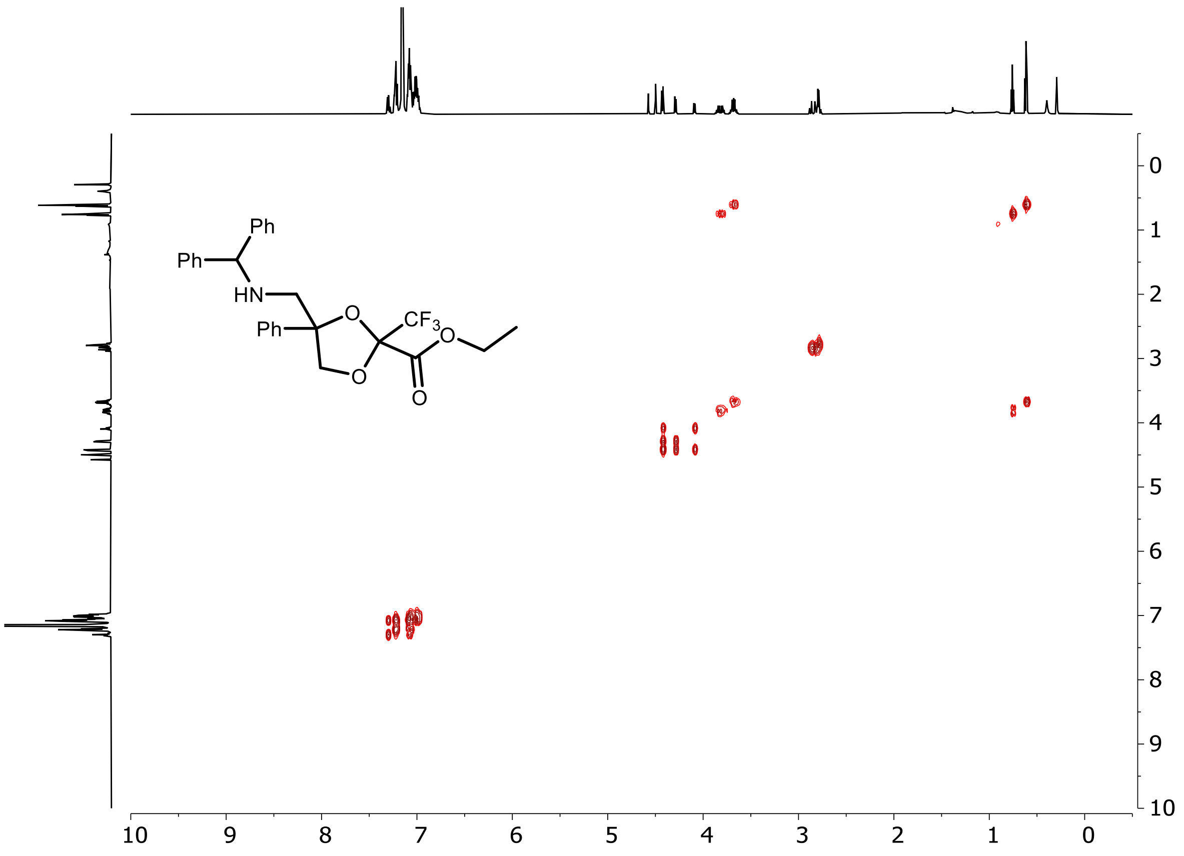

(16), HSQC, C_6D_6

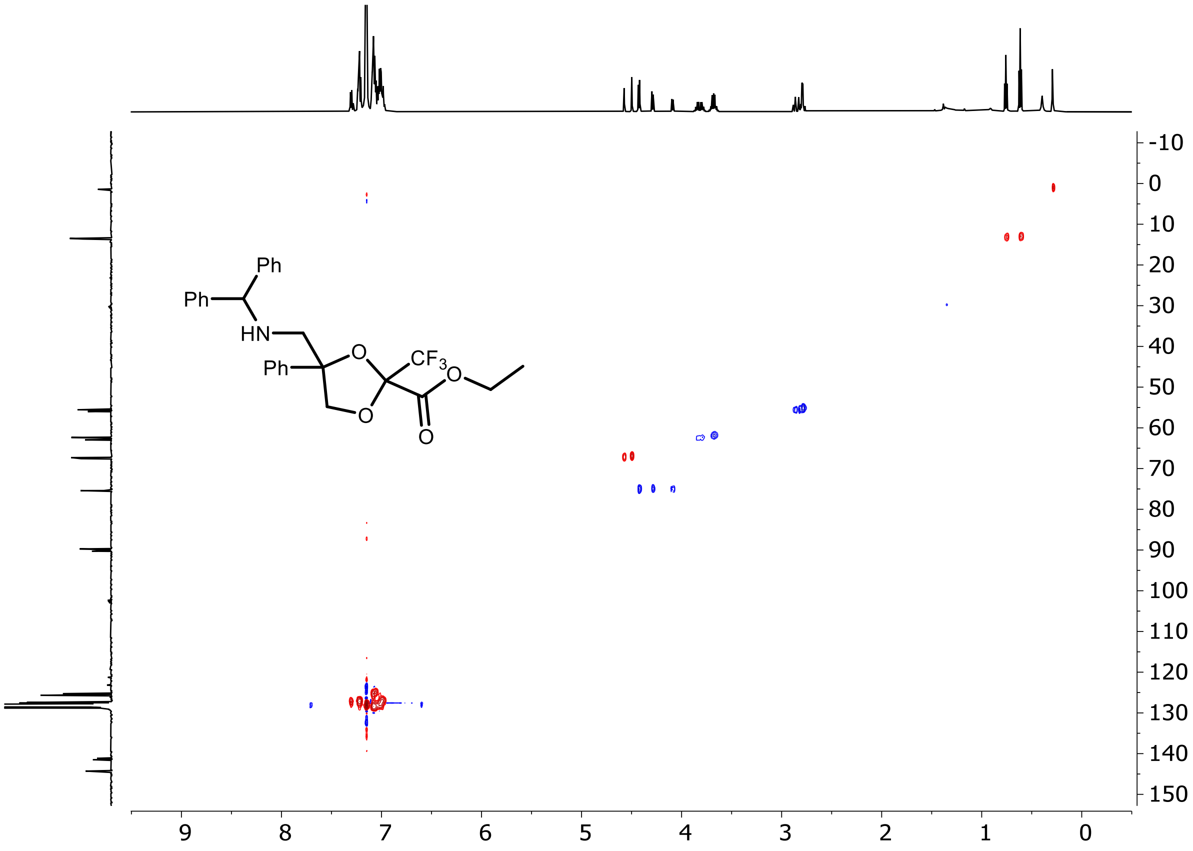

(16), HMBC, C_6D_6

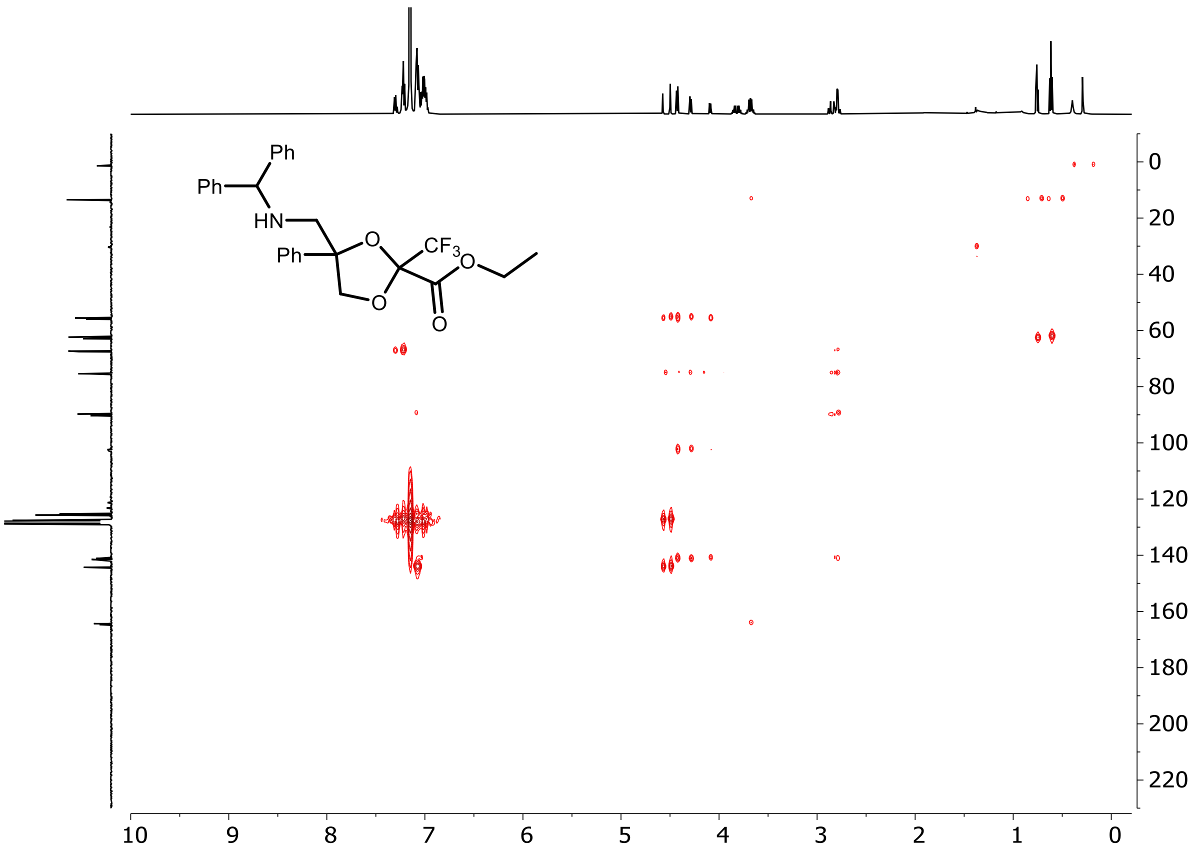

(21), ^{1}H, CDCl3, 400 MHz

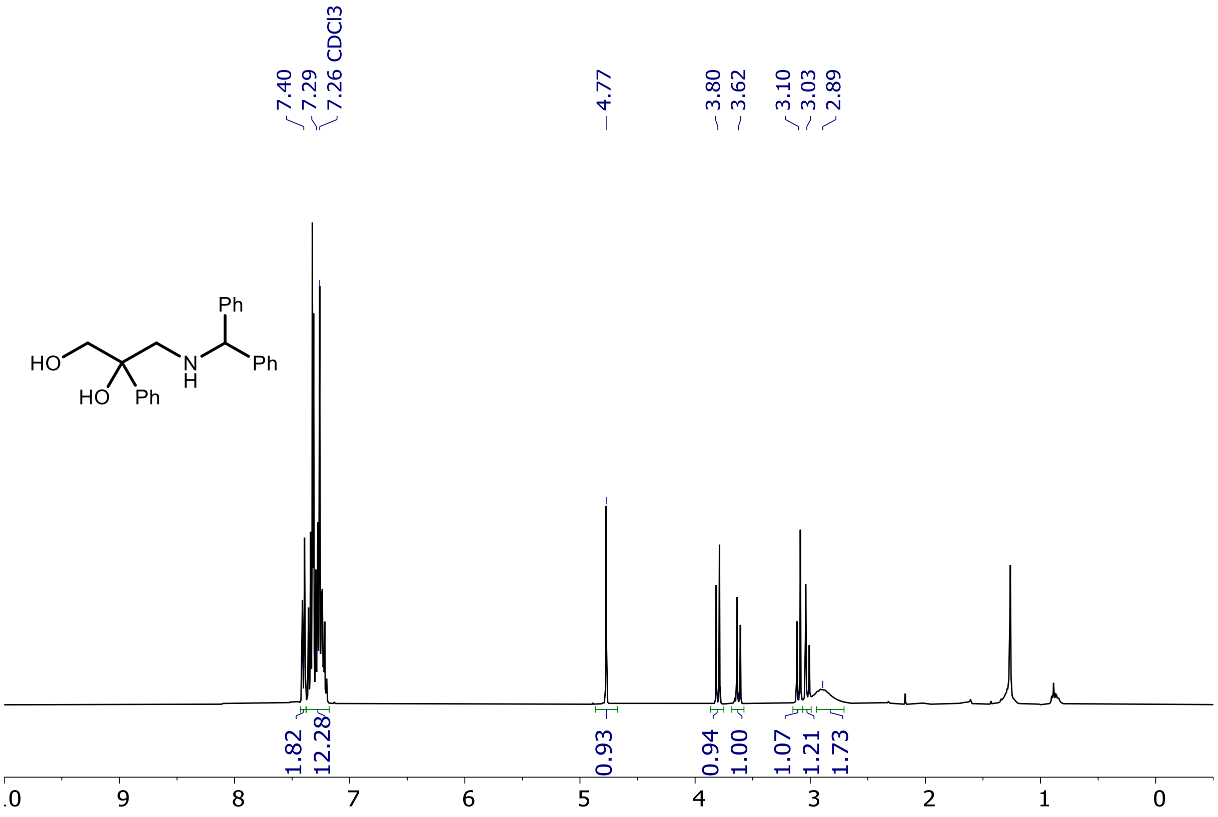

(21), ^{13}C, CDCl3, 101 MHz

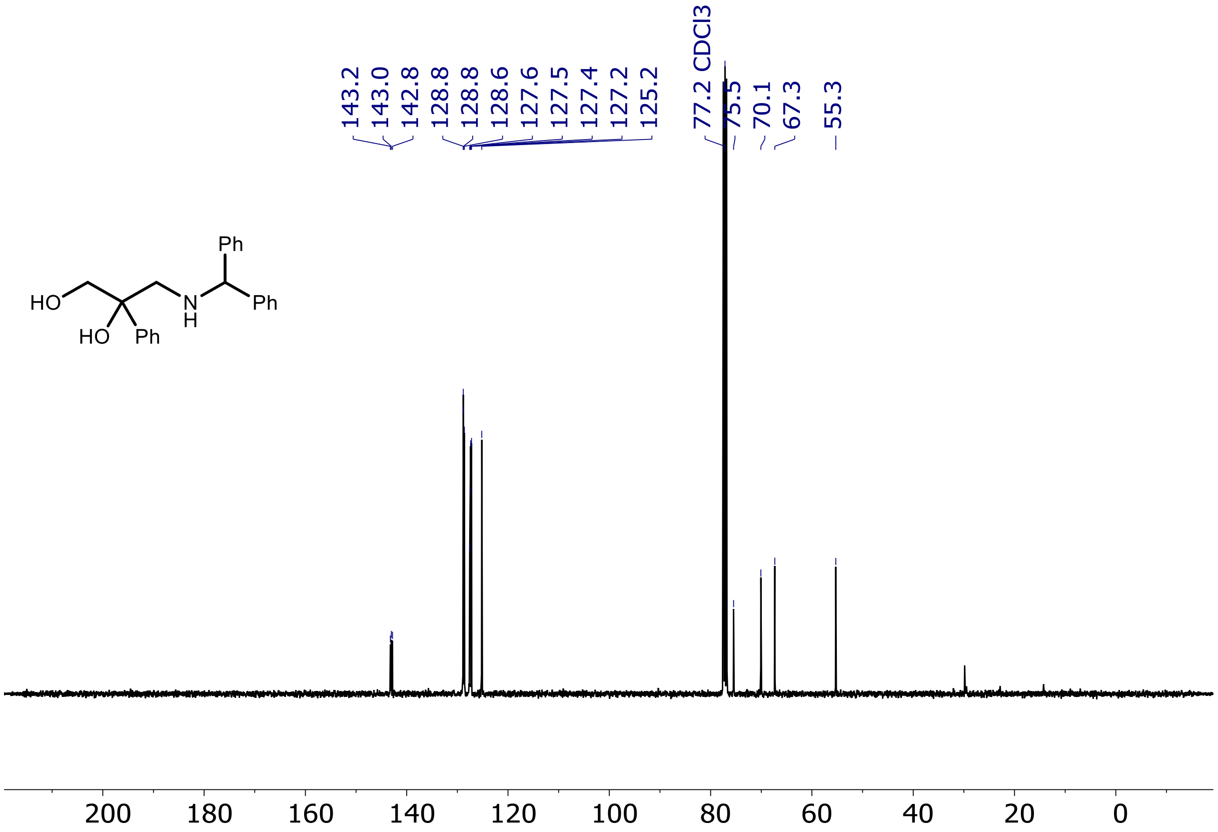

Supplement: File 1 — Experimental procedures, characterization data and copies of NMR spectra. [file Beilstein_J_Org_Chem-20-1671-s001.pdf]
